# Epidemiology beyond its limits

**DOI:** 10.1126/sciadv.abn3328

**Published:** 2022-06-08

**Authors:** Lauren E. McCullough, Maret L. Maliniak, Avnika B. Amin, Julia M. Baker, Davit Baliashvili, Julie Barberio, Chloe M. Barrera, Carolyn A. Brown, Lindsay J. Collin, Alexa A. Freedman, David C. Gibbs, Maryam B. Haddad, Eric W. Hall, Sarah Hamid, Kristin R. V. Harrington, Aaron M. Holleman, John A. Kaufman, Mohammed A. Khan, Katie Labgold, Veronica C. Lee, Amyn A. Malik, Laura M. Mann, Kristin J. Marks, Kristin N. Nelson, Zerleen S. Quader, Katherine Ross-Driscoll, Supriya Sarkar, Monica P. Shah, Iris Y. Shao, Jonathan P. Smith, Kaitlyn K. Stanhope, Marisol Valenzuela-Lara, Miriam E. Van Dyke, Kartavya J. Vyas, Timothy L. Lash

**Affiliations:** 1 Department of Epidemiology, Rollins School of Public Health, Emory University, Atlanta, GA, USA.; 2 Amgen Inc., Thousand Oaks, CA, USA.; 3 Department of Population Health Sciences, Huntsman Cancer Institute, University of Utah, Salt Lake City, UT, USA.; 4 Institute for Policy Research, Northwestern University, Evanston, IL, USA.; 5 School of Public Health, Oregon Health & Science University, Portland, OR, USA.; 6 Yale Institute for Global Health, Yale University, New Haven, CT, USA.; 7 Department of Surgery, Emory University School of Medicine, Atlanta, GA, USA.; 8 ViiV Healthcare, Research Triangle Park, NC, USA.; 9 Department of Health Policy and Management, Yale School of Public Health, New Haven, CT, USA.; 10 Department of Gynecology and Obstetrics, Emory University School of Medicine, Atlanta, GA, USA.

## Abstract

In 1995, journalist Gary Taubes published an article in *Science* titled “Epidemiology faces its limits,” which questioned the utility of nonrandomized epidemiologic research and has since been cited more than 1000 times. He highlighted numerous examples of research topics he viewed as having questionable merit. Studies have since accumulated for these associations. We systematically evaluated current evidence of 53 example associations discussed in the article. Approximately one-quarter of those presented as doubtful are now widely viewed as causal based on current evaluations of the public health consensus. They include associations between alcohol consumption and breast cancer, residential radon exposure and lung cancer, and the use of tanning devices and melanoma. This history should inform current debates about the reproducibility of epidemiologic research results.

## Teaser

Many examples used in Taubes’ influential 1995 article to denigrate epidemiology now have important public health implications.

## INTRODUCTION

In July of 1995, journalist Taubes (*1*) published an influential news article in *Science* titled, “Epidemiology faces its limits.” The main emphasis was that epidemiology was “straying beyond the limits of the possible no matter how carefully the studies are done” because nonrandomized studies are so “plagued with biases, uncertainties, and methodological weaknesses that they may be inherently incapable of accurately discerning… weak associations.” The science of epidemiology, Taubes argued, studied a “mind-numbing array of potential disease-causing agents,” yielding an onslaught of “constitutionally contradictory” results.

The article prompted an immediate defense by the epidemiologic community (*2*–*7*), but together, these defenses have been cited eightfold less often than the original Taubes paper. Therefore, despite these contemporary objections, the Taubes article has exerted a sustained influence on epidemiologists’ self-impression and the impression of epidemiology in the wider scientific community. It has been cited more than 1000 times (fig. S1), with most citations relying on the article to cast doubt on the value of epidemiologic research (*8*). Examples of papers published in 2019–2021 (of 59 citations during this time period) cite the 1995 Taubes article to support the following contentions:

1) “Identifying major risk factors for disease can lead to misleading results when studying large numbers of variables interacting at multiple social, spatial, and temporal scales” (*9*)

2) “In [evidence-based medicine], observational studies are taken to suffer from different biases and confounding” (*10*)

3) “[epidemiology is among the fields] where serious concerns and debates about low reproducibility have been raised” (*11*)

4) “[…] the vast majority of epidemiological literature has important limitations, which are magnified when assessing low-dose effects” (*12*)

In the article by Taubes, he highlighted a substantial number of associations between exposures and outcomes as having questionable merit. In his introduction to a table listing many of these example associations, Taubes wrote, “Are these dangers real? As the saying goes, you be the judge.” To our knowledge, no one has taken up the challenge. Twenty-five years after the publication of this influential article, we systematically evaluated the epidemiologic evidence surrounding each of Taubes’ example associations. For each, we searched for a recent and representative meta-analysis of the posited association and whether there have been public health recommendations by credible authorities made to address it.

## RESULTS

### Updated causal evaluations

We identified 70 example associations in the Taubes (1995) paper. We excluded 14 duplicate associations and 3 associations that were too vaguely worded for causal evaluation (e.g., “a few drugs and cancer”). Thus, 53 associations remained for assessment of causality (table S1). We obtained a recent and representative meta-analysis for 49 of these associations. We did not identify meta-analyses related to the use of phenoxy herbicides on lawns and malignant lymphoma in dogs, having shorter or longer than average menstrual cycles and breast cancer, or the use of the antihypertension medication reserpine and breast cancer. We obtained a credible consensus statement for evaluating causality for 51 associations. No consensus statements from authoritative public health organizations were found for having shorter or longer than average menstrual cycles and breast cancer or the use of the antihypertension medication reserpine and breast cancer. These two associations were automatically deemed indeterminate.

Table 1 presents comparisons of the assumed causal evaluation by Taubes (1995) and our group (2021). Full results with identified meta-analyses and consensus statements by association are included in the Supplementary Materials (table S2, A to WW). Among these, 11 example associations were provided by Taubes as evidence of causal associations, and our group concurs. They include the association between smoking and lung cancer, human papillomavirus and cancer, and ionizing radiation and cancer. One association (saccharine and bladder cancer) was presented as an example of a noncausal association, and our group concurs. The remaining 41 associations were suggested by Taubes as likely false-positive associations. Of these, our group considered 11 (27%) as causal and 5 (12%) as noncausal based on public health statements by authoritative bodies. For example, the associations between alcohol consumption and breast cancer, residential radon exposure and lung cancer, and use of tanning devices and melanoma were suggested by Taubes as false positives and are now considered causal. On the basis of our updated assessment, examples of noncausal associations include those between coffee and pancreatic cancer and induced abortion and breast cancer. The remaining 25 (61%) associations were deemed indeterminate by our group. These included the association between hair dyes and several hematologic malignancies, eating red meat and breast cancer, and drinking chlorinated tap water and bladder cancer.

**Table 1.** Updated causal evaluations (2021) for abstracted associations discussed in Taubes paper (1995). Full results with identified meta-analyses and consensus statements are included in the Supplementary Materials. ACOG, American College of Obstetricians and Gynecologists; AHA, American Heart Association; APHA, American Public Health Association; AUA, American Urological Association; FDA, U.S. Food and Drug Administration; IARC, International Agency for Research on Cancer; NCI, U.S. National Cancer Institute; WCRF/AICR, World Cancer Research Fund/American Institute for Cancer Research.

**Exposure**	**Outcome**	**Taubes causal evaluation** **(1995)**	**Updated causal** **evaluation (2021)**	**Evaluation by authoritative** **bodies**
Human papillomavirus	Cancer	Causal	Causal	IARC: group 1 carcinogen
Ionizing radiation	Cancer	Causal	Causal	IARC: group 1 carcinogen
Hepatitis virus	Cancer	Causal	Causal	IARC: group 1 carcinogen
Smoking	Lung cancer	Causal	Causal	IARC: group 1 carcinogen
Cigarette smoke	Cancer	Causal	Causal	IARC: group 1 carcinogen
Sunlight	Skin cancer	Causal	Causal	IARC: group 1 carcinogen
Alcohol	Cancer	Causal	Causal	IARC: group 1 carcinogen
Asbestos	Cancer	Causal	Causal	IARC: group 1 carcinogen
Occupational steel (coke-oven) exposure	Lung cancer	Causal	Causal	IARC: group 1 carcinogen
Early childbirth (maternal age)	Breast cancer	Causal	Causal	WCRF/AICR: established cause of breast cancer
Human T cell leukemia virus	Cancer	Causal	Causal	IARC: group 1 carcinogen
Obesity	Esophageal cancer	Indeterminate	Causal	IARC: group 1 carcinogen
Cigarette smoke	Pancreatic cancer	Indeterminate	Causal	IARC: group 1 carcinogen
Lengthy occupational exposure to dioxin (2,3,7,8-tetrachlorodibenzodioxin)	All cancers	Indeterminate	Causal	IARC: group 1 carcinogen
Alcohol	Breast cancer	Indeterminate	Causal	IARC: group 1 carcinogen
Residential radon	Lung cancer	Indeterminate	Causal	IARC: group 1 carcinogen
Eating red meat	Colon cancer	Indeterminate	Causal	IARC: group 2A probably carcinogenic to humans
High birthweight	Breast cancer	Indeterminate	Causal	WCRF/AICR: “There is strong evidence that factors that lead to a greater birthweight, or its consequences, increase the risk of premenopausal breast cancer”
Oral contraceptive use	Breast cancer	Indeterminate	Causal	IARC: group 1 carcinogen for estrogen-progestogen oral contraceptives (combined) and breast cancer
Sun lamp use	Melanoma	Indeterminate	Causal	IARC: group 1 carcinogen
Eating processed meats	Colon cancer	Indeterminate	Causal	IARC: group 1 carcinogen
Breastfeeding	Childhood leukemia/brain cancer	Indeterminate	Causal	NCI: “Being breastfed and having been exposed to routine childhood infections are both associated with a lowered risk of developing childhood leukemia”
High-alcohol mouthwash	Mouth cancer	Indeterminate	Indeterminate	FDA: “The available data do not support a causal relationship between the use of alcohol-containing mouthrinses and oral cancer”
Electromagnetic fields	Childhood leukemia/brain cancer	Indeterminate	Indeterminate	IARC: group 2B possibly carcinogenic to humans for limited evidence in relation to childhood leukemia. Inadequate evidence for all other cancers.
Traffic density	Childhood leukemia/brain cancer	Indeterminate	Indeterminate	IARC: “[...] consistent association between exposure to benzene and AML for children, and coherence with findings for adult AML and benzene exposure, but could not rule out chance, bias, and confounding as alternative explanations”
High-cholesterol diet	Rectal cancer in men	Indeterminate	Indeterminate	WCRF/AICR 2018 Colorectal Cancer Report: limited to no conclusion for cholesterol
Douching	Cervical cancer	Indeterminate	Indeterminate	APHA: linked with cervical cancer but difficult to determine causality
Occupational stress	Colorectal cancer	Indeterminate	Indeterminate	NCI: “Although stress can cause a number of physical health problems, the evidence that it can cause cancer is weak”
Smoking	Fatal breast cancer	Indeterminate	Indeterminate	Komen: “Growing evidence suggests smoking lowers the chances of survival for women with breast cancer”
Hair dyes	Myeloma	Indeterminate	Indeterminate	IARC: group 3 not classifiable
Chlorinated tap water	Bladder cancer	Indeterminate	Indeterminate	IARC: group 3 not classifiable as to its carcinogenicity to humans; there was inadequate evidence of carcinogenicity in both humans and animals for chlorinated drinking water
Eating yogurt	Ovarian cancer	Indeterminate	Indeterminate	WCRF/AICR 2014 Ovarian Cancer Report: limited to no conclusion for milk and dairy products
Hair dyes	Lymphoma	Indeterminate	Indeterminate	IARC: group 3 not classifiable
Electromagnetic fields	Brain cancer	Indeterminate	Indeterminate	IARC: group 2B possibly carcinogenic to humans for limited evidence in relation to childhood leukemia. Inadequate evidence for all other cancers.
Hair dyes	Leukemia	Indeterminate	Indeterminate	IARC: group 3 not classifiable
Smoking	Breast cancer	Indeterminate	Indeterminate	IARC evaluation noted a positive association but did not state that tobacco smoking was a cause of breast cancer.
Diet high in saturated fat	Lung cancer	Indeterminate	Indeterminate	WCRF/AICR 2017 Lung Cancer Report: limited to no conclusion for total fat and animal fat
Electromagnetic fields	Leukemia	Indeterminate	Indeterminate	IARC: group 2B possibly carcinogenic to humans for limited evidence in relation to childhood leukemia. Inadequate evidence for all other cancers.
Fat intake	Breast cancer	Indeterminate	Indeterminate	WCRF/AICR 2017 Breast Cancer Report: limited to no conclusion for fats and oils, vegetable fat, fatty acid composition, trans fatty acids, cholesterol
Maternal smoking	Childhood leukemia/brain cancer	Indeterminate	Indeterminate	IARC: Limited evidence in humans for tobacco smoking and childhood leukemia (in smokers’ children); “[...] a fairly consistent association of paternal tobacco smoking with childhood cancers is beginning to emerge, which is stronger in studies with more specific exposure assessments”
Eating red meat	Breast cancer	Indeterminate	Indeterminate	WCRF/AICR 2017 Breast Cancer Report: limited to no conclusion for red and processed meat
Electromagnetic fields	Breast cancer	Indeterminate	Indeterminate	IARC: group 2B possibly carcinogenic to humans for limited evidence in relation to childhood leukemia. Inadequate evidence for all other cancers.
Coffee	Heart disease	Indeterminate	Indeterminate	AHA: “Moderate coffee drinking (1–2 cups/day) does not seem to be harmful”
Consuming olive oil	Breast cancer	Indeterminate	Indeterminate	WCRF/AICR 2017 Breast Cancer Report: limited to no conclusion for fats and oils
Use of phenoxy herbicides on lawns	Malignant lymphoma in dogs	Indeterminate	Indeterminate	IARC: There is limited evidence in experimental animals for the carcinogenicity of 2,4-dichlorophenoxyacetic acid
Having shorter or longer than average menstrual cycles	Breast cancer	Indeterminate	Indeterminate	None identified
Antihypertensive medication reserpine	Breast cancer	Indeterminate	Indeterminate	None identified
Coffee	Pancreatic cancer	Indeterminate	Not causal	IARC: There is evidence suggesting lack of carcinogenicity
Vasectomy	Prostate cancer	Indeterminate	Not causal	AUA: “Clinicians do not need to routinely discuss prostate cancer […] in prevasectomy counseling of patients because vasectomy is not a risk factor for these conditions. Standard (evidence strength: grade B)”
Breast self-examination	Breast cancer mortality	Indeterminate	Not causal	ACOG: “Breast self- examination is not recommended in average- risk women because there is a risk of harm from false-positive test results and a lack of evidence of benefit.”
Abortion	Breast cancer	Indeterminate	Not causal	ACOG: no causal relationship
Dichlorodiphenyltrichloroethane	Breast cancer	Indeterminate	Not causal	IARC: no association overall; however, the potential influence of age at exposure to dichlorodiphenyltrichloroethane in relation to risk of breast cancer remains of interest
Saccharine	Bladder cancer	Not causal	Not causal	IARC: group 3 not classifiable

### Summary-relative risks by causal evaluation status

**Fig. 1.**
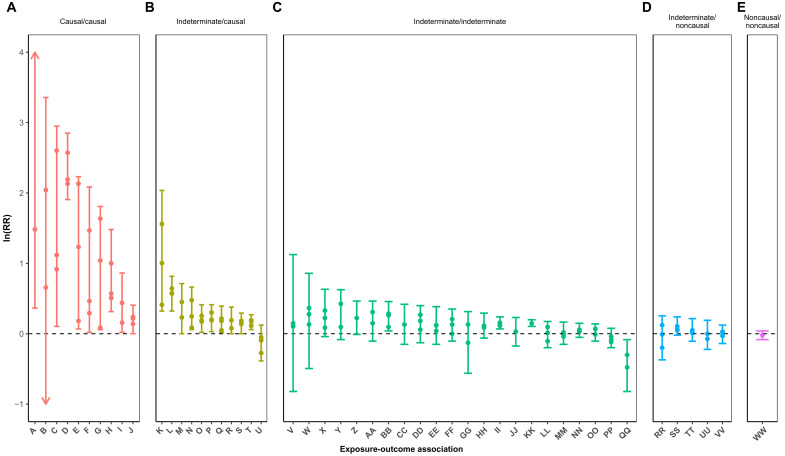
Selected meta-analytic estimates were strongest for associations deemed causal by both Taubes (1995) and our group (2021) and were more modest for associations deemed indeterminate by Taubes (1995) and causal by our group (2021). Meta-analytic estimates by causal evaluation (Taubes’ 1995 Assessment/Updated 2021 Assessment) for (**A**) causal/causal, (**B**) indeterminate/causal, (**C**) indeterminate/indeterminate, (**D**) indeterminate/noncausal, and (**E**) noncausal/noncausal. Arrows indicate that estimate is beyond the *y*-axis range. Points indicate meta-analytic estimates, and segment end caps indicate minimum and maximum values of the 95% confidence intervals (CIs). Key: A, human papillomavirus and cancer; B, ionizing radiation and cancer; C, hepatitis and cancer; D, smoking and lung cancer; E, cigarette smoke and cancer; F, sunlight and skin cancer; G, alcohol and cancer; H, asbestos and cancer; I, occupational steel (coke-oven) exposure and lung cancer; J, early childbirth (maternal age) and breast cancer; K, obesity and esophageal cancer; L, cigarette smoking and pancreatic cancer; M, lengthy occupational dioxin (2,3,7,8-tetrachlorodibenzodioxin) and cancer; N, alcohol and breast cancer; O, residential radon and lung cancer; P, eating red meat and colon cancer; Q, birthweight and breast cancer; R, oral contraceptive use and breast cancer; S, sun lamp use and melanoma; T, eating processed meat and colon cancer; U, breastfeeding and brain cancer/leukemia in children; V, high-alcohol mouthwash and mouth cancer; W, electromagnetic fields and brain cancer/leukemia in children; X, traffic density and brain cancer/leukemia in children; Y, high-cholesterol diet and rectal cancer; Z, douching and cervical cancer; AA, occupational stress and colorectal cancer; BB, smoking and fatal breast cancer; CC, hair dyes and myeloma; DD, drinking chlorinated tap water and bladder cancer; EE, eating yogurt and ovarian cancer; FF, hair dyes and lymphoma; GG, electromagnetic fields and brain cancer; HH, hair dyes and leukemia; II, smoking and breast cancer; JJ, diet high in saturated fat and lung cancer (among nonsmokers); KK, electromagnetic fields and leukemia; LL, fat intake and breast cancer; MM, maternal smoking and brain cancer/leukemia in children; NN, eating red meat and breast cancer; OO, electromagnetic fields and breast cancer; PP, coffee and heart disease; QQ, olive oil and breast cancer; RR, coffee and pancreatic cancer; SS, vasectomy and prostate cancer; TT, breast self-examination and breast cancer mortality; UU, abortion and breast cancer; VV, dichlorodiphenyltrichloroethane and breast cancer; WW, saccharine and bladder cancer.

Figure 1 examines the selected meta-analytic estimates by causal evaluation status. Meta-analytic estimates for the association between human T cell leukemia virus and cancer were not included in the figure as the estimates were not summary-relative risks. Meta-analyses for ionizing radiation and cancer were included, but estimates are summary excess-relative risks. Meta-analytic estimates were most robust for associations deemed causal by both Taubes (1995) and our group (2021). Meta-analytic estimates were more modest and closer to the null for associations Taubes (1995) deemed indeterminate and for those that our group (2021) determined as causal, with the exception of the association between obesity and esophageal cancer.

## DISCUSSION

Twenty-five years ago, Gary Taubes’ widely cited report in *Science* questioned the utility of epidemiologic research. To bolster his case, Taubes selected numerous examples of research topics that he viewed as unlikely to have public health implications, building an argument for the “inherent incapability” of epidemiologic studies to draw causal inferences for weak associations [risk ratios (RRs) < 3]. Research has since accumulated about these example topics. We evaluated the current evidence regarding Taubes’ selected examples, finding that 11 of the 41 associations (~27%) suggested in his initial paper to be false positives are now widely viewed as causal based on current evaluations of the public health consensus.

With the exception of the association between obesity and esophageal cancer, none of the associations deemed indeterminate in 1995 and subsequently classified as causal by our assessment reached Taubes’ cited threshold (RR ≥ 3) for “serious consideration.” However, each association had sufficient scientific data to support one or more consensus statements from reputable national and international agencies, including the U.S. National Cancer Institute (NCI), U.S. Centers for Disease Control and Prevention (CDC), International Agency for Research on Cancer (IARC), and World Health Organization (WHO)—supporting the value of epidemiologic research in identifying even modest associations.

The example of the association between alcohol consumption and breast cancer risk is illustrative. Taubes (*1*) quotes an epidemiologist as saying “nobody is convinced of the breast cancer–alcohol connection ‘except Walt Willett.’” The U.S. NCI and the IARC both now describe alcohol drinking as a known cause of breast cancer. Taubes (*1*) also casts doubt on the association between exposure to radon and lung cancer risk. Homebuyers across the United States routinely have homes tested for radon, and high levels abated because the association is considered causal. We wonder whether those who continue to cite Taubes (1995) as a critique of the science of epidemiology are aware that one-quarter of the associations selected by Taubes to erroneously illustrate the limits reached by epidemiology now have sufficient causal evidence and corresponding public health policies to reduce their risks.

Our group deemed 25 of the original examples indeterminate, reflecting a lack of public health consensus regarding causality (causal or noncausal) for these associations and consistent with Taubes’ previous assessment. Many studies of the proposed associations were too specific for judgment, limiting consensus. For example, IARC concluded that extremely low-frequency magnetic fields are possibly carcinogenic to humans due to evidence of an increased risk of childhood leukemia at high levels of magnetic fields, but associations between electromagnetic fields and other cancers are unclear (*13*). Other associations have sufficient emerging evidence (e.g., automotive benzene, a byproduct of traffic-related air pollution, and childhood cancer) that authoritative bodies have already made recommendations to limit exposure (*14*). Causal associations for many examples are limited by important confounding factors, which may be challenging to disentangle. For instance, the association between high-cholesterol diet and rectal cancer risk may be confounded by certain foods or food groups. Similarly, current smoking and drinking may confound the association between mouthwash and oral cancer (*15*). Inconsistencies observed for douching and cervical cancer may arise because individuals with certain characteristics (i.e., lower education, multiple sexual partners, and poverty) are also at a greater risk of sexually transmitted infections and bacterial vaginosis; they may douche secondary to infection-related symptoms rather than as a part of their normal hygienic practice (*16*). Additional data on these topics would be prudent to further explore causality.

Our efforts to update the example associations cited by Taubes were limited by two factors. In many instances, the 1995 examples were vague: Did Taubes mean “fatal breast” cancer as de novo metastatic disease or breast cancer–specific mortality? Still, others were too specific: drinking >3.3 liters of fluid (particularly chlorinated tap water) per day and bladder cancer. Thus, the 53 identified associations and causal evaluations are based on our own interpretations of Taubes’ 1995 report. For some associations, there was wide variation in meta-analytic estimates, limiting comparability. This was, in part, due to different meta-analyses using different exposure definitions, cut points, or outcomes. Our analysis of the meta-analytic estimates was not intended to be exhaustive. We selected up to three meta-analyses per association to examine patterns in the strength of associations by causal evaluation—not to represent all meta-analyses for these associations or estimate causal effects. Consensus statements by authoritative groups were based on the state of the science and viewpoints of the experts at the time these exposures were reviewed. Thus, they could change as new data become available. Nonetheless, we took a systematic approach to identifying and summarizing the example associations put forth by Taubes, considering both meta-analyses and authoritative consensus statements.

Inherent in the title and a common theme throughout Taubes’ 1995 article is that epidemiology is “stretched to its limits or beyond” and “at the edge of what can be done” (*1*). However, epidemiology has experienced tremendous innovation over the past few decades. The proliferation of large prospective cohorts and cohort consortia has allowed for better study of rare exposures and outcomes with adequate power rather than relying on retrospective case-control studies, which were the target of much of Taubes’ criticisms. These cohorts and consortia also allow for replication of studies across independent populations and in different contexts, which can help inform the impact of confounding (for example) on associations (confounding by health care access would presumably be less concerning in countries with universal health care).

Advances in technology have resulted in an explosion of data and the ability to readily link data across sources such as electronic health records, biobanks, vital records, disease surveillance registries, claims and administrative data, geospatial data, and mobile data. These can be used on their own for conducting research (e.g., studies using nationwide health registries or administrative databases) or linked to enrich cohort or case-control study data.

Technological advances such as wearable devices for health and environmental monitoring and omics data have and will continue to improve exposure assessment—which Taubes (*1*) describes as “the most pernicious” bias that plagues epidemiologic study of risk factors. For example, ongoing studies are examining the use of distinct somatic mutational signatures to identify past exposures (*17*). One of the aims of the Sherlock-Lung study is to examine mutational signatures of over 2000 lung cancers in never smokers with the goal of linking them to known risk factors such as radon, secondhand tobacco smoke, and air pollution (*18*). These technologically advanced exposure assessment tools raise new challenges and are often prohibitive for large-scale epidemiologic studies. However, if proven useful, then methods already exist to integrate these exposure assessment tools by conducting case-control studies nested within cohorts, validation studies, and/or quantitative bias analyses (*19*). With regard to outcome assessment, molecular and omics data have greatly accelerated our understanding of the heterogeneity of diseases. For example, molecular classification and gene expression–based assays are already part of routine clinical care for breast cancer (*20*) and are increasingly incorporated in studies of risk factors to elucidate differential risks by subtype (*21*).

In recent years, causal inference methods have come to the forefront of epidemiology (*22*) with increasing use of causal diagrams [e.g., directed acyclic graphs (*23*)] and study designs [e.g., negative control (*24*–*27*) and Mendelian randomization (MR) studies (*28*, *29*), difference-in-difference methods (*30*–*32*), and regression discontinuity designs (*33*, *34*)] to rule out noncausal associations. For example, MR studies, which use genetic variants as instrumental variables, have provided additional insight into the role of alcohol consumption and the risk of cancer with compelling evidence for head/neck and esophageal cancer (*35*–*37*), although evidence from three recent MR studies for breast cancer is less clear (*38*–*40*). MR studies attempt to address issues of confounding and reverse causation but require certain assumptions be met (as do all statistical and epidemiologic methods), can require large sample sizes for genetic variant(s) that explain only a small proportion of variation in an exposure, and may be subject to other biases (*41*, *42*). Thus, casual evaluation of risk factors, especially for those where randomized controlled trials are infeasible or unethical, will continue to require synthesizing evidence across various sources [see textbox 3 from Krieger and Davey Smith (*22*) for triangulating evidence from eight different epidemiologic study designs for the example of smoking and low birthweight]. This triangulation of evidence is necessary to overcome the shortcomings of all study types from mechanistic studies and cohort studies to randomized controlled trials and meta-analyses.

Many of the associations selected by Taubes as examples to denigrate epidemiologic research have proven to have important public health implications—as evidenced by policy recommendations from reputable national and international agencies to reduce risks arising from the associations. The utility of epidemiologic research in this regard is all the more impressive when one remembers that the associations were selected because Taubes thought they would prove to be false positives. Twenty-five years later, epidemiology has reached beyond its limits. This history should inform current debates about the rigor and reproducibility of epidemiologic research results.

## MATERIALS AND METHODS

The project was led by two doctorally trained epidemiologists with faculty appointments (T.L.L. and L.E.M.), with most of the work completed by a subset of doctoral students in the epidemiology department at Emory University’s Rollins School of Public Health. This team drafted a protocol, reviewed, revised, and registered it with the Center for Open Science (https://osf.io/4sfrb).

### Protocol for identification of meta-analyses and consensus statements by authoritative bodies

Briefly, the Taubes paper was split into pages, and each page was independently evaluated by two reviewers who identified and abstracted each example epidemiologic association mentioned on the page. The work of the two reviewers was evaluated by a third independent reviewer, and any discrepancies were resolved by consensus among the three.

Each association was then assigned, at random, to a pair of reviewers to search for recent meta-analyses and a consensus statement that address the association. Three reviewers were assigned for each association. Two reviewers independently identified relevant search terms and combinations of search terms from National Library of Medicine Medical Subject Headings (NLM MeSH) for use in identifying meta-analyses and consensus statements. A third reviewer submitted terms and noted discrepancies. All three reviewers worked to achieve agreement on final search strings.

#### Meta-analyses

Each reviewer was assigned to search a single database: PubMed [including term “meta-analysis” (publication type)], Cochrane, PROSPERO (meta-analysis filter), or Embase (review filter). Relevant titles and abstracts on the associations of interest—published after 31 July 1995—were entered into Mendeley reference management software (*43*). We included one meta-analysis published before 31 July 1995 for the association between saccharine and bladder cancer because there were no later meta-analyses. Full text review was completed by two independent reviewers, and a third reviewer noted discrepancies. All reviewers worked to achieve consensus on the inclusion of up to three meta-analyses per association—prioritizing the degree the meta-analysis addressed the association of interest, the quality of the meta-analysis, and year of publication, in that order. Because many of the example associations in the paper by Taubes (*1*) were not specific in defining the exposure or outcome, we also prioritized meta-analyses that were aligned with the public health consensus statements. For instance, for associations where the outcome was described by Taubes (*1*) as cancer, we prioritized meta-analyses where the outcome was a cancer site that has been assessed by an expert working group for its relation with an exposure—such as cervical cancer for the association between human papillomavirus and cancer. For selected studies, each reviewer documented the following information: association of interest, first author’s last name and publication year, title of article, quote with study objective (generally abstracted from the body of paper), study designs included, number of studies included, final conclusion, pooled measure of association(s) and confidence interval(s) (CI), and any relevant additional information.

To visualize the strength of associations by causal evaluation, we plotted the log of the summary estimate and the minimum and maximum values of the corresponding 95% CIs from up to three meta-analyses for each exposure-outcome association. In several meta-analyses, separate summary estimates for different exposure measurements, outcomes, and subgroups were provided. We selected one summary estimate (and corresponding 95% CI) per meta-analysis that we deemed representative of the exposure-outcome association of interest. If the exposure-outcome association described by Taubes (*1*) was nonspecific (e.g., alcohol and cancer), then we aimed to select meta-analytic estimates used by authoritative bodies for issuing scientific evaluations/policy recommendations—or were in accordance with them.

#### Consensus statements

Each reviewer was tasked to identify and list disease-specific public health governmental and nongovernmental organizations (national or international) listed within the first 10 pages of the Google search results. In addition, the following general public health governmental and nongovernmental organizations were searched if they were not included in the first 10 pages of Google search results: IARC, U.S. CDC, WHO, American Public Health Association (APHA), U.S. Environmental Protection Agency, U.S. Agency for Toxic Substances and Disease Registry, U.S. Preventive Services Task Force, and the U.S. National Academy of Medicine. For each identified organization, it was determined whether the organization had a webpage dedicated to scientific evaluations, policy statements, guidelines, or recommendations. If confirmed, then that page was searched for statements relevant to the association of interest. If no such page could be located, then the outcome of interest was searched to identify specific evaluations, policy statements, guidelines, or recommendations. At least two independent reviewers compiled statements—published after 31 July 1995—that addressed the associations of interest and cited evidence from meta-analyses or individual studies. A third independent reviewer noted discrepancies, and all reviewers worked to achieve consensus. For each source, reviewers documented the following information: association of interest, organization and link to statement, title of the statement, author (if applicable), year of publication, and final evaluation or recommendation(s)—citing quotations from the statement.

### Protocol for causal determination

The abstracted information was reviewed by two new randomly assigned evaluators to determine whether the association was viewed as causal. Independently, reviewers classified each association as causal, noncausal, or indeterminate based on the criteria below. A third reviewer identified discrepancies, which were resolved by consensus among all reviewers.

Consensus statements were primarily used for determining causality. We prioritized consensus statements that were based on comprehensive reviews conducted by a group of expert scientists for establishing causality (e.g., IARC monographs). Associations where the meta-analysis was not accompanied by a consensus statement, or accompanied by a vague consensus statement, were assessed for causality based on the following guidelines:

1) Consistency of results (number of replicated studies across multiple populations)

2) Dose response (e.g., examine associations in quartiles of exposure)

3) Demonstration of results in a randomized trial

4) Biological plausibility (basic science supportive of causal mechanism)

5) Temporal sequence (association measured using cohort or other study design in which exposure was measured before outcome)

Associations without any published meta-analyses or consensus statements were classified as indeterminate. Final determination of whether the association should be considered causal, noncausal, or indeterminate was made taking all guidelines into account.

## Supplementary Materials

### This PDF file includes:

Supplementary Abbreviations Fig. S1 Tables S1 and S2A–WW References
