# Supplementary material for: Epidemiology beyond its limits
Source: Sci Adv. 2022 Jun 8;8(23):eabn3328. doi: 10.1126/sciadv.abn3328 (PMC9176748; doi:10.1126/sciadv.abn3328)
Supplement: Supplementary file 1 — Supplementary Abbreviations Fig. S1 Tables S1 and S2A–WW References [file sciadv.abn3328_sm.pdf]

Science Advances
AAAS

Supplementary Materials for

Epidemiology beyond its limits

Lauren E. McCullough *et al.*

Corresponding author: Lauren E. McCullough, lauren.mccullough@emory.edu

Sci. Adv. **8**, eabn3328 (2022)
DOI: 10.1126/sciadv.abn3328

This PDF file includes:

Supplementary Abbreviations
Fig. S1
Tables S1 and S2A–WW
References

Supplementary Abbreviations

ALL	Acute lymphoblastic leukemia
AML	Acute myeloid leukemia
BSE	Breast self-examination
CDC	United States Centers for Disease Control and Prevention
CI	Confidence interval
CLL	Chronic lymphocyte leukemia
DDE	Dichlorodiphenyldichloroethylene
DDT	Dichlorodiphenyltrichloroethane
ELF	Extremely low-frequency
EMF	Electromagnetic fields
EPA	United States Environmental Protection Agency
ER	Estrogen receptor
FDA	United States Food and Drug Administration
FL	Follicular lymphoma
Gy	Gray
HBV	Hepatitis B virus
HPV	Human papillomavirus
IARC	International Agency for Research on Cancer
MDM	Meat derived mutagenic activity
NCI	United States National Cancer Institute
PR	Progesterone receptor
PUFAs	Polyunsaturated fatty acids
SLL	Small lymphocytic lymphoma
Sv	Sievert
UV	Ultraviolet
WCRF/AICR	World Cancer Research Fund/American Institute for Cancer Research
WHO	World Health Organization

Fig. S1.

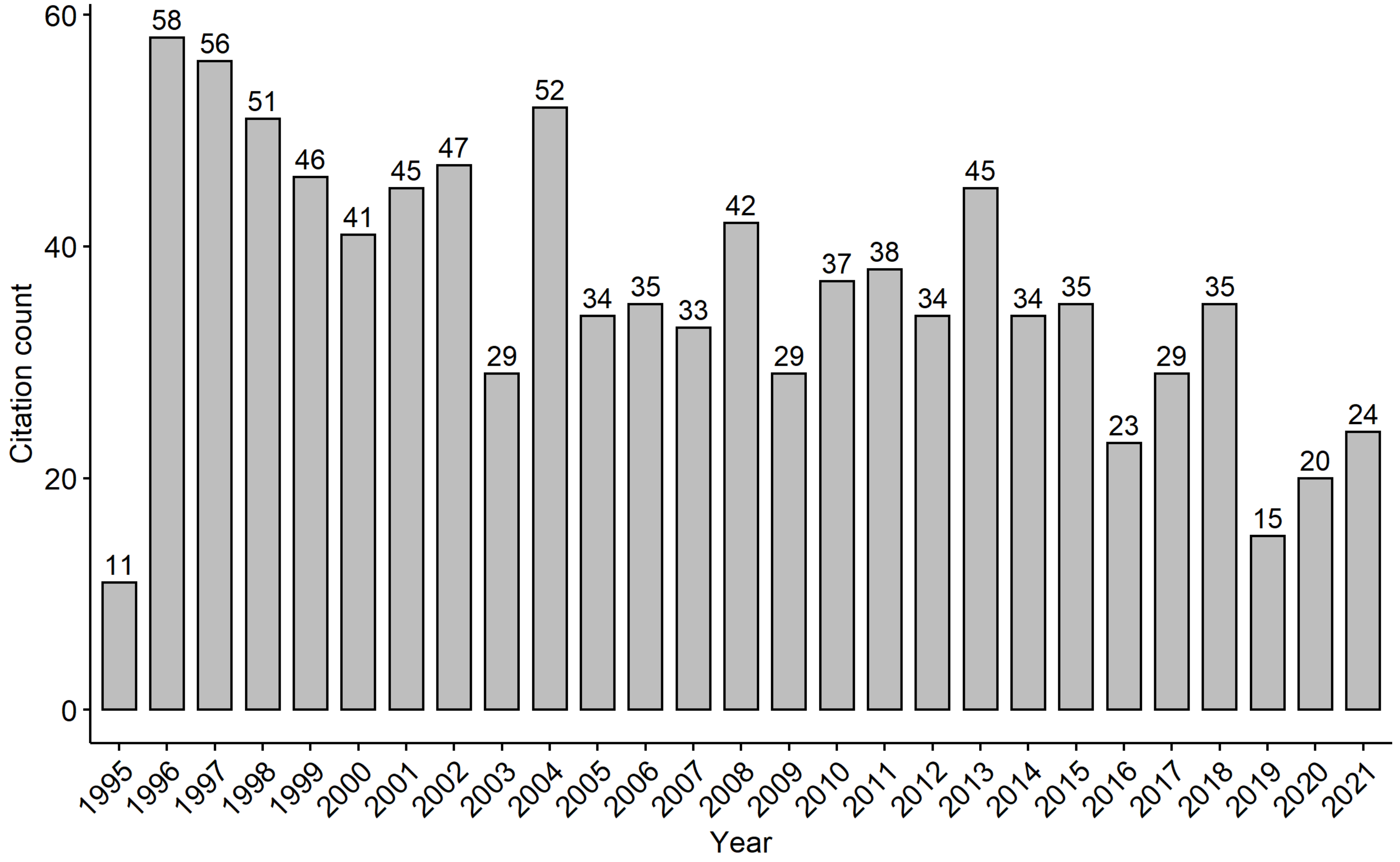

Number of citations per year for the article "Epidemiology faces its limits" by Gary Taubes published in 1995 in *Science*. Citation data were retrieved and analyzed using Google Scholar and Publish or Perish software (available from https://harzing.com/resources/publish-or-perish) (*8*). Citations without a publication year were excluded (n=47). All data and R code are available at https://github.com/mmaliniak/Taubes_project

Table S1.

**Exposure (as worded in the article)**	**Outcome (as worded in the article)**	**Location in article**	**Assumed evaluation of association by Taubes**
Smoking	Lung cancer	Page 164, middle of column 2<br>Page 165, bottom of column 1<br>Page 168, middle of column 3	Causal
Sunlight	Skin cancer	Page 165, bottom of column 1	Causal
Cigarette smoke	Cancer	Page 165, Box titled “Sizing up the Cancer Risks”	Causal
Alcohol	Cancer	Page 165, Box titled “Sizing up the Cancer Risks”<br>Page 167, middle of column 1	Causal
Ionizing radiation	Cancer	Page 165, Box titled “Sizing up the Cancer Risks”	Causal
Asbestos	Cancer	Page 165, Box titled “Sizing up the Cancer Risks”	Causal
Hepatitis-R virus	Cancer	Page 165, Box titled “Sizing up the Cancer Risks”	Causal
Human T cell leukemia virus	Cancer	Page 165, Box titled “Sizing up the Cancer Risks”	Causal
Human papillomavirus	Cancer	Page 165, Box titled “Sizing up the Cancer Risks”	Causal
Early childbirth	Breast cancer	Page 167, top of column 3	Causal
Steel-workers/coke-oven workers	Lung cancer	Page 169, middle of column 2	Causal
Residential radon	Lung cancer	Page 164, top of column 1	Indeterminate
Pesticide residues (DDT)<br>Pesticide exposure, indicated by high residues in blood	Breast cancer	Page 164, top of column 1<br>Page 165, Box titled “Sizing up the Cancer Risks”	Indeterminate
Abortion	Breast cancer	Page 164, middle of column 1<br>Page 165, Box titled “Sizing up the Cancer Risks”	Indeterminate
Electromagnetic fields (EMF)	Brain cancer	Page 164, middle of column 1	Indeterminate
Electromagnetic fields (EMF)	Leukemia	Page 164, middle of column 1<br>Page 169, top of column 2	Indeterminate
Hair dyes	Lymphomas	Page 164, bottom of column 1	Indeterminate
Hair dyes	Myelomas	Page 164, bottom of column 1	Indeterminate

Hair dyes	Leukemia	Page 164, bottom of column 1	Indeterminate
Coffee	Pancreatic cancer	Page 164, bottom of column 1<br>Page 167, middle of column 1<br>Page 168, top of column 3	Indeterminate
Coffee	Heart disease	Page 164, bottom of column 1	Indeterminate
Alcohol	Breast cancer	Page 164, bottom of column 2<br>Page 168, bottom of column 3<br>Page 169, top of column 1	Indeterminate
High cholesterol diet	Rectal cancer in men	Page 165, Box titled “Sizing up the Cancer Risks”	Indeterminate
Eating yogurt at least once a month	Ovarian cancer	Page 165, Box titled “Sizing up the Cancer Risks”	Indeterminate
Smoking more than 100 cigarettes in a lifetime	Breast cancer	Page 165, Box titled “Sizing up the Cancer Risks”	Indeterminate
High-fat diet	Breast cancer	Page 165, Box titled “Sizing up the Cancer Risks”<br>Page 167, middle-bottom of column 3	Indeterminate
Lengthy occupational exposure to dioxin	All cancers	Page 165, Box titled “Sizing up the Cancer Risks”	Indeterminate
Douching once a week	Cervical cancer	Page 165, Box titled “Sizing up the Cancer Risks”	Indeterminate
Regular use of high-alcohol mouthwash	Mouth cancer	Page 165, Box titled “Sizing up the Cancer Risks”	Indeterminate
Use of phenoxy herbicides on lawns	Malignant lymphoma in dogs	Page 165, Box titled “Sizing up the Cancer Risks”	Indeterminate
Weighing 3.6 kilograms or more at birth	Breast cancer	Page 165, Box titled “Sizing up the Cancer Risks”	Indeterminate
Vasectomy	Prostate cancer	Page 165, Box titled “Sizing up the Cancer Risks”	Indeterminate
Drinking more than 3.3 liters of fluid (particularly chlorinated tap water) a day	Bladder cancer	Page 165, Box titled “Sizing up the Cancer Risks”	Indeterminate
Experiencing psychological stress in the workplace	Colorectal cancer	Page 165, Box titled “Sizing up the Cancer Risks”	Indeterminate
Diet high in saturated fat	Lung cancer in nonsmoking women	Page 165, Box titled “Sizing up the Cancer Risks”	Indeterminate
Eating more than 20 grams of processed meats (i.e., bologna) a day	Colon cancer	Page 165, Box titled “Sizing up the Cancer Risks”	Indeterminate
Eating red meat five or more times a week	Colon cancer	Page 165, Box titled “Sizing up the Cancer Risks”	Indeterminate

Occupational exposure to electromagnetic fields (EMF)	Breast cancer	Page 165, Box titled “Sizing up the Cancer Risks”<br>Page 169, top of column 2	Indeterminate
Smoking two packs of cigarettes a day	Fatal breast cancer	Page 165, Box titled “Sizing up the Cancer Risks”	Indeterminate
Eating red meat twice a day	Breast cancer	Page 165, Box titled “Sizing up the Cancer Risks”	Indeterminate
Regular cigarette smoking	Pancreatic cancer	Page 165, Box titled “Sizing up the Cancer Risks”	Indeterminate
Ever having used a sun lamp	Melanoma	Page 165, Box titled “Sizing up the Cancer Risks”	Indeterminate
Having shorter or longer than average menstrual cycles	Breast cancer	Page 165, Box titled “Sizing up the Cancer Risks”	Indeterminate
Obesity in men (the heaviest 25% of those in the study)	Esophageal cancer	Page 165, Box titled “Sizing up the Cancer Risks”	Indeterminate
Consuming olive oil once a day or less	Breast cancer	Page 165, Box titled “Sizing up the Cancer Risks”	Indeterminate
Electromagnetic fields (EMF) from power lines	Leukemia and brain cancer in children	Page 167, bottom of column 1	Indeterminate
Breastfeeding	Leukemia and brain cancer in children	Page 167, top-middle of column 2	Indeterminate
Maternal smoking	Leukemia and brain cancer in children	Page 167, top-middle of column 2	Indeterminate
Traffic density	Leukemia and brain cancer in children	Page 167, top-middle of column 2	Indeterminate
Breast self-examination (BSE)	Breast cancer mortality	Page 167, bottom of column 2	Indeterminate
Oral contraceptive use	Breast cancer	Page 167, bottom of column 3<br>Page 169, top of column 1	Indeterminate
Anti-hypertension medication reserpine	Breast cancer	Page 168, top of column 3	Indeterminate
Saccharine	Bladder cancer	Page 169, bottom of column 2 - top of column 3	Not causal

Abstracted associations from Taubes Paper (*1*) in order of assumed causal evaluation.

Table S2A-WW.

A. Human papillomavirus (HPV) and cancer

Assumed causal evaluation as presented by Taubes in 1995: Causal
Updated causal evaluation after review in 2021: Causal

	First author, year	Number and designs of primary studies	Exposure	Outcome	Summary estimate (95% CI)	Dose-response assessment	Dose-response summary estimate (95% CI)
Meta-analyses	Koshiol et al., 2008 (*44*)	41 studies (3 clinical trials, 38 cohort)	Persistent HPV infection (overall HPV) vs. HPV-negative	Cervical neoplasia	104.2 (11.9, 912.1)	Minimum duration of HPV persistence (months)	HPV-negative: 1.0 (ref)<br>≤12: 63.9 (7.9, 516.7)<br>>12: 223.0 (39.0, 1,273.3)
	Saulle et al., 2015 (*45*)	5 case–control studies	HPV infection	Oral cavity cancer	4.40 (1.75, 11.06)		
	Saulle et al., 2015 (*45*)	2 case–control studies	HPV infection	Tonsil cancer	4.41 (1.44, 13.46)		

	Public health organization	Excerpt from statement	Citation
Consensus statements	IARC, 2007 (*46*)	There is sufficient evidence in humans for the carcinogenicity of HPV 16 in the cervix, vulva (basaloid and warty tumours), vagina, penis (basaloid and warty tumours), anus, oral cavity and oropharynx. There is sufficient evidence in humans for the carcinogenicity of HPV 18 in the cervix. There is sufficient evidence in humans for the carcinogenicity of HPV types 31, 33, 35, 39, 45, 51, 52, 56, 58, 59 and 66 in the cervix. […] There is inadequate evidence in humans for the carcinogenicity of HPV in the oesophagus, lung, colon, ovary, breast, prostate, urinary bladder and nasal and sinonasal cavities. […] HPV types 16, 18, 31, 33, 35, 39, 45, 51, 52, 56, 58, 59 and 66 are carcinogenic to humans (Group 1).	IARC Working Group on the Evaluation of Carcinogenic Risks to Humans. Human Papillomaviruses. Lyon (FR): International Agency for Research on Cancer; 2007. (IARC Monographs on the Evaluation of Carcinogenic Risks to Humans, No. 90.) Available from: https://www.ncbi.nlm.nih.gov/books/NBK321760/
	CDC, 2018 (*47*)	Almost all cervical cancer is caused by HPV. And some cancers of the vulva, vagina, penis, anus, and oropharynx (back of the throat, including the base of the tongue and tonsils) are also caused by HPV.	Division of Cancer Prevention and Control, Centers for Disease Control and Prevention, Page last reviewed: September 3, 2020 https://www.cdc.gov/cancer/hpv/basic_info/cancers.htm

B. Ionizing radiation and cancer

Assumed causal evaluation as presented by Taubes in 1995: Causal
Updated causal evaluation after review in 2021: Causal

Meta-analyses	First author, year	Number and designs of primary studies	Exposure	Outcome	Summary estimate (95% CI)	Dose-response assessment	Dose-response summary estimate (95% CI)
	Ron, 1995 (*48*)	5 cohort studies	Per Gy	Thyroid cancer	7.7 (2.1, 28.7)[a]	Per Gy	7.7 (2.1, 28.7)
	Cardis, 2007 (*49*)	Multinational retrospective cohort study (15-Country Study)	Per Sv	Leukemia (excluding CLL)	1.93 (0, 7.14)[a]	Per Sv	1.93 (0, 7.14)
	Daniels, 2011 (*50*)	18 retrospective cohorts/nested case–control	At 100 mGy	Leukemia (excluding CLL)	0.17 (0.09, 0.26)[a]		

[a]Studies of ionizing radiation reported estimates as excess relative risks, which is the relative risk (RR) minus 1 (e.g., 8.7 – 1 = 7.7)

Consensus statements	Public health organization	Excerpt from statement	Citation
	IARC, 2000 (*51*)	X-radiation and γ-radiation are carcinogenic to humans (Group 1).	IARC Monographs on the Evaluation of Carcinogenic Risks to Humans Volume 75. Available from: https://www.ncbi.nlm.nih.gov/books/NBK401325/

C. Hepatitis and cancer

Assumed causal evaluation as presented by Taubes in 1995: Causal
Updated causal evaluation after review in 2021: Causal

	First author, year	Number and designs of primary studies	Exposure	Outcome	Summary estimate (95% CI)	Dose-response assessment	Dose-response summary estimate (95% CI)
Meta-analyses	Cho, 2011 (*52*)	47 studies (37 case–control, 10 cohort)	HBV infection (HBsAg/HBV DNA)	Hepatocellular carcinoma	13.5 (9.5, 19.1)		
	Chen, 2016 (*53*)	9 studies (5 case–control, 4 cohort)	6.5 log10 copies/ml vs.2 log10 copies/ml HBV DNA level	Hepatocellular carcinoma	3.06 (1.11, 8.44)	log_{10} copies/ml HBV DNA level:<br>2<br>4.5<br>5.5<br>6.5	1.0 (ref)<br>1.65 (0.94, 2.92)<br>2.20 (1.00, 4.85)<br>3.06 (1.11, 8.44)
	Li, 2018 (*54*)	58 studies (49 case–controll, 9 cohort)	HBV infection (seropositive for HBsAg or HBV DNA)	non-Hodgkin lymphoma	2.50 (2.20, 2.83)		

	Public health organization	Excerpt from statement	Citation
Consensus statements	American Cancer Society, CDC, NCI, and the North American Association of Central Cancer Registries, 2016 (*55*)	Viral hepatitis is an important cause of hepatocellular carcinoma, the most common histologic type of liver cancer.	Annual Report to the Nation on the Status of Cancer, 1975-2012, featuring the increasing incidence of liver cancer. Available from: https://pubmed.ncbi.nlm.nih.gov/26959385/

	IARC, 2012 (*56*)	There is sufficient evidence in humans for the carcinogenicity of chronic infection with HBV. Chronic infection with HBV causes hepatocellular carcinoma. Also, positive associations have been observed between chronic infection with HBV and cholangiocarcinoma and non-Hodgkin lymphoma. Chronic infection with HBV is carcinogenic to humans (Group 1).	IARC Monographs on the Evaluation of Carcinogenic Risks to Humans Volume 100 B. Available from: https://www.ncbi.nlm.nih.gov/books/NBK304348/

D. Smoking and lung cancer

Assumed causal evaluation as presented by Taubes in 1995: Causal
Updated causal evaluation after review in 2021: Causal

Meta-analyses	First author, year	Number and designs of primary studies	Exposure	Outcome	Summary estimate (95% CI)	Dose-response assessment	Dose-response summary estimate (95% CI)
	Gandini, 2008 (*57*)	21 cohort/case–control studies	Current smoking vs. never	Lung cancer	8.96 (6.73, 12.11)	1-9 cig/day<br>10-19 cig/day<br>≥20 cig/day	Men:<br>1.39 (1.28, 1.50)<br>2.67 (2.11, 3.37)<br>13.70 (7.40, 25.50)<br>Women:<br>1.49 (1.37, 1.61)<br>3.30 (2.59, 4.20)<br>24.10 (12.70, 45.90)
	Lee, 2012 (*58*)	287 studies (52 prospective, 204 case–control, 6 other)	Current smoking vs. never	Lung cancer	8.43 (7.63, 9.31)	Frequency (cigarettes/day):<br>5<br>20<br>45<br>Duration (years):<br>20<br>35<br>50	Frequency (cigarettes/day):<br>3.25 (3.17, 3.34)<br>5.30 (5.18, 5.43)<br>10.17 (9.89, 10.45)<br>Duration (years):<br>2.46 (2.31, 2.63)<br>6.17 (5.80, 6.55)<br>13.46 (12.61, 14.36)
	Ordóñez-Mena, 2016 (*59*)	19 population-based cohorts	Current smoking vs. never	Lung cancer	13.1 (9.90, 17.3)		

Consensus statements	Public health organization	Excerpt from statement	Citation
	IARC, 2012 (*60*)	Tobacco smoking causes cancers of the lung, oral cavity, naso-, oro- and hypopharynx, nasal cavity and accesory sinuses, larynx, oesophagus, stomach, pancreas, colorectum, liver, kidney (body and pelvis), ureter, urinary bladder, uterine cervix and ovary (mucinous), and myeloid leukaemia. Tobacco smoking is carcinogenic to humans (Group 1).	IARC Monographs on the Evaluation of Carcinogenic Risks to Humans Volume 100 E. Available from: https://www.ncbi.nlm.nih.gov/books/NBK304391/

E. Cigarette smoke and cancer

Assumed causal evaluation as presented by Taubes in 1995: Causal
Updated causal evaluation after review in 2021: Causal

Meta-analyses	First author, year	Number and designs of primary studies	Exposure	Outcome	Summary estimate (95% CI)	Dose-response assessment	Dose-response summary estimate (95% CI)
	Gandini, 2008 (*57*)	12 cohort/case–control studies	Current smoking vs. never	Oral cavity cancer	3.43 (2.37, 4.94)		
	Lee, 2012 (*58*)	267 studies (52 prospective, 209 case–control, 5 nested case–control, 1 case–cohort)	Current smoking vs. never	Lung cancer	8.43 (7.63, 9.31)	Frequency (cigarettes/day):<br>5<br>20<br>45<br>Duration (years):<br>20<br>35<br>50	Frequency (cigarettes/day):<br>3.25 (3.17, 3.34)<br>5.30 (5.18, 5.43)<br>10.17 (9.89, 10.45)<br>Duration (years):<br>2.46 (2.31, 2.63)<br>6.17 (5.80, 6.55)<br>13.46 (12.61, 14.36)
	Ordóñez-Mena, 2016 (*59*)	19 population-based cohorts	Current smoking vs. never	Colorectal cancer	1.20 (1.07, 1.34)		

Consensus statements	Public health organization	Excerpt from statement	Citation
	IARC, 2012 (*60*)	Tobacco smoking causes cancers of the lung, oral cavity, naso-, oro- and hypopharynx, nasal cavity and accesory sinuses, larynx, oesophagus, stomach, pancreas, colorectum, liver, kidney (body and pelvis), ureter, urinary bladder, uterine cervix and ovary (mucinous), and myeloid leukaemia. Tobacco smoking is carcinogenic to humans (Group 1).	IARC Monographs on the Evaluation of Carcinogenic Risks to Humans Volume 100 E. Available from: https://www.ncbi.nlm.nih.gov/books/NBK304391/

F. Sunlight and skin cancer

Assumed causal evaluation as presented by Taubes in 1995: Causal
Updated causal evaluation after review in 2021: Causal

	First author, year	Number and designs of primary studies	Exposure	Outcome	Summary estimate (95% CI)	Dose-response assessment	Dose-response summary estimate (95% CI)
Meta-analyses	Gandini, 2005 (*61*)	5 cohort, 51 case–control, 2 nested case–control	Total sun exposure (highest vs. lowest categories)	Melanoma	1.34 (1.02, 1.77)	Sunburns (highest vs.lowest)	2.03 (1.73, 2.37)
	Dennis, 2008 (*62*)	51 studies (16 of which were population-based)	Ever sunburned in lifetime	Melanoma	1.59 (1.37, 1.83)	5 sunburns per decade over lifetime	2.66 (2.25, 3.13)
	Olsen, 2011 (*63*)	11 case–control studies	Presence of solar keratoses (any vs. none)	Melanoma	4.34 (2.34, 8.04)	Number of "painful" sunburns<br>Never<br>1-5<br>6-25<br>26+	 <br>1.0 (ref)<br>1.12 (0.87, 1.43)<br>1.66 (1.24, 2.24)<br>3.22 (2.04, 5.09)

	Public health organization	Excerpt from statement	Citation
Consensus statements	NCI, 2017 (*64*)	The sun, sunlamps, and tanning booths all give off ultraviolet (UV) radiation. Exposure to UV radiation causes early aging of the skin and damage that can lead to skin cancer.	Sunlight. Last reviewed May 1, 2020. Available from: https://www.cancer.gov/about-cancer/causes-prevention/risk/sunlight
	IARC, 2012 (*65*)	There is sufficient evidence in humans for the carcinogenicity of solar radiation. Solar radiation causes cutaneous malignant melanoma, squamous cell carcinoma of the skin and basal cell carcinoma of the skin. […] Solar radiation is carcinogenic to humans (Group 1).	IARC Monographs on the Evaluation of Carcinogenic Risks to Humans Volume 100 D. Available from: https://www.ncbi.nlm.nih.gov/books/NBK304366/#a006.sec5

G. Alcohol and cancer

Assumed causal evaluation as presented by Taubes in 1995: Causal
Updated causal evaluation after review in 2021: Causal

Meta-analyses	First author, year	Number and designs of primary studies	Exposure	Outcome	Summary estimate (95% CI)	Dose-response assessment	Dose-response summary estimate (95% CI)
	Bagnardi, 2015 (*66*)	52 studies (5 cohort, 47 case–control)	Heavy drinkers vs. non-drinkers/occasional drinkers	Oral/pharyngeal cancer	5.13 (4.31, 6.10)	Non-drinkers<br>Light drinking<br>Moderate drinking<br>Heavy drinking	1.0 (ref)<br>1.13 (1.00, 1.26)<br>1.83 (1.62, 2.07)<br>5.13 (4.31, 6.10)
	Jayasekara, 2016 (*67*)	3 cohort studies	Highest vs. lowest average alcohol intake during lifetime/over time	Upper aero-digestive tract cancer	2.83 (1.73, 4.62)		
	Choi, 2018 (*68*)	60 cohort studies	Light drinking (≤1 drink/day) vs. none	Female breast cancer	1.09 (1.06, 1.12)	≤0.5 drink/day<br>≤1 drink/day<br>1-2 drinks	1.04 (1.01, 1.07)<br>1.09 (1.06, 1.12)<br>1.13 (1.11, 1.15)

Consensus statements	Public health organization	Excerpt from statement	Citation
	CDC, 2019 (*69*)	All types of alcoholic drinks, including red and white wine, beer, cocktails, and liquor, are linked with cancer. The more you drink, the higher your cancer risk.	Alcohol and Cancer. Last reviewed July 8, 2019. Available from: https://www.cdc.gov/cancer/alcohol/index.htm
	IARC, 2012 (*60*)	There is sufficient evidence in humans for the carcinogenicity of alcohol consumption. Alcohol consumption causes cancers of the oral cavity, pharynx, larynx, oesophagus, colorectum, liver (hepatocellular carcinoma) and female breast. […] Alcohol consumption is carcinogenic to humans (Group 1).	IARC Monographs on the Evaluation of Carcinogenic Risks to Humans Volume 100 E. Available from: https://www.ncbi.nlm.nih.gov/books/NBK304391/

H. Asbestos and cancer

Assumed causal evaluation as presented by Taubes in 1995: Causal
Updated causal evaluation after review in 2021: Causal

	First author, year	Number and designs of primary studies	Exposure	Outcome	Summary estimate (95% CI)	Dose-response assessment	Dose-response summary estimate (95% CI)
Meta-analyses	Camargo, 2011 (*70*)	18 cohort studies	Occupational exposure to asbestos	Ovarian cancer	1.77 (1.37, 2.28)	High occupational exposure to asbestos	2.78 (1.36, 5.66)
	Lenters, 2011 (*71*)	19 studies (18 cohorts, 1 population-based case–control study)	Per 100 fiber-years/mL of cumulative exposure to asbestos	Lung cancer	1.66 (1.53, 1.79)		
	Ngamwong, 2015 (*72*)	6 cohort studies	Asbestos-exposed[a] and non-smokers[b] vs. non-exposed to asbestos and non-smokers	Lung cancer	2.72 (1.67, 4.40)		

[a]Asbestos exposure was arbitrarily taken as more than 100 air-borne fiber-yr/ml of environmental air for >5% of work time; subjects having lower and shorter fiber exposures were deemed non-exposed

[b]Subjects were considered smokers if smoked >15 cigarettes/day; subjects with lower cigarette consumption were deemed non-smokers

	Public health organization	Excerpt from statement	Citation
Consensus statements	IARC, 2012 (*73*)	There is sufficient evidence in humans for the carcinogenicity of all forms of asbestos (chrysotile, crocidolite, amosite, tremolite, actinolite, and anthophyllite). Asbestos causes mesothelioma and cancer of the lung, larynx, and ovary. Also, positive associations have been observed between exposure to all forms of asbestos and cancer of the pharynx, stomach, and colorectum. For cancer of the colorectum, the Working Group was evenly divided as to whether the evidence was strong enough to warrant classification as sufficient. […]	IARC Monographs on the Evaluation of Carcinogenic Risks to Humans Volume 100 C. Available from: https://www.ncbi.nlm.nih.gov/books/NBK304375/
	EPA, 2016 (*74*)	A large number of occupational studies have reported that exposure to asbestos via inhalation causes lung cancer and mesothelioma (a rare cancer of the membranes lining the abdominal cavity and surrounding internal organs). […] EPA has classified asbestos as Group A, human carcinogen.	Asbestos. Last reviewed in September 2016. Available from: https://www.epa.gov/sites/production/files/2016-10/documents/asbestos.pdf

I. Occupational steel (coke-oven) exposure and lung cancer

Assumed causal evaluation as presented by Taubes in 1995: Causal
Updated causal evaluation after review in 2021: Causal

	First author, year	Number and designs of primary studies	Exposure	Outcome	Summary estimate (95% CI)	Dose-response assessment	Dose-response summary estimate (95% CI)
Meta-analyses	Armstrong, 2004 (*75*)	10 studies (9 cohort, 1 nested case–control)	at 100 µg/m^3 years cumulative benzo[*a*]pyrene among coke oven workers	Lung cancer	1.17 (1.12, 1.22)		
	Singh, 2018 (*76*)	7 cohort studies	Coal/coke industry workers	Lung cancer	1.55 (1.02, 2.37)		

	Public health organization	Excerpt from statement	Citation
Consensus statements	EPA, 2000 (*77*)	Epidemiologic studies of coke oven workers have reported an increase in cancer of the lung, trachea, bronchus, kidney, prostate, and other sites. […] EPA has classified coke oven emissions as a Group A, known human carcinogen.	Coke Oven Emissions. Last reviewed on January 2000. Available from: https://www.epa.gov/sites/production/files/2016-09/documents/coke-oven-emissions.pdf
	IARC, 2012 (*78*)	There is sufficient evidence in humans for the carcinogenicity of coke production. Coke production causes cancer of the lung.	IARC Monographs on the Evaluation of Carcinogenic Risks to Humans Volume 100 F. Available from: https://www.ncbi.nlm.nih.gov/books/NBK304422/#a018.sec5

J. Early childbirth (maternal age) and breast cancer

Assumed causal evaluation as presented by Taubes in 1995: Causal
Updated causal evaluation after review in 2021: Causal

Meta-analyses	First author, year	Number and designs of primary studies	Exposure	Outcome	Summary estimate (95% CI)	Dose-response assessment	Dose-response summary estimate (95% CI)
	Ma, 2006 (*79*)	1 cohort study, 6 population-based case–control studies, 2 hospital-based case–control studies	Oldest vs. youngest age categories	ER+PR+ Breast Cancer	1.27 (1.07, 1.50)		
	Reeves, 2009 (*80*)	4 cohort studies, 5 case–control studies	Age at 1st birth (30+ vs. <20 years)	Breast cancer (Ductal)	1.24 (1.20, 1.29)		
	Lambertini, 2016 (*81*)	15 studies (3 prospective cohorts, 10 case–control, 2 pooled analyses of cohort studies and population-based case–control studies)	Advanced maternal age (typically defined as age at 1st birth >24 years) vs. young age at 1st birth	Breast cancer (luminal subtype)	1.15 (1.00, 1.32)		

Consensus statements	Public health organization	Excerpt from statement	Citation
	NCI, 2016 (*82*)	Women who have their first full-term pregnancy at an early age have a decreased risk of developing breast cancer later in life. […] This risk reduction is limited to hormone receptor–positive breast cancer; age at first full-term pregnancy does not appear to affect the risk of hormone receptor-negative breast cancer.	Reproductive History and Cancer Risk. Last reviewed on November 9, 2016. Available from: https://www.cancer.gov/about-cancer/causes-prevention/risk/hormones/reproductive-history-fact-sheet
	WCRF/ AICR, 2018 (*83*)	In addition to the findings on diet, nutrition and physical activity […], other established causes of breast cancer include: Early menarche (before the age of 12), late natural menopause (after the age of 55), not bearing children and first pregnancy over the age of 30 all increase lifetime exposure to oestrogen and progesterone and the risk of breast cancer. The reverse also applies: late menarche, early menopause, bearing children and pregnancy before the age of 30 all reduce the risk of breast cancer.	Continuous Update Project Expert Report 2018. Diet, nutrition, physical activity and breast cancer. Last updated in 2018. Available from: https://www.wcrf.org/wp-content/uploads/2021/02/Breast-cancer-report.pdf

K. Obesity and esophageal cancer

Assumed causal evaluation as presented by Taubes in 1995: Indeterminate
Updated causal evaluation after review in 2021: Causal

Meta-analyses	First author, year	Number and designs of primary studies	Exposure	Outcome	Summary estimate (95% CI)	Dose-response assessment	Dose-response summary estimate (95% CI)
	Hoyo, 2012 (*84*)	10 case–control studies, 2 cohort studies	BMI ≥40 vs.<25 kg/m^2	Oesophageal adenocarcinoma	4.76 (2.96, 7.66)	per unit increase in BMI	1.09 (1.06, 1.12)
	Turati, 2013 (*85*)	22 studies (12 case–control, 10 prospective studies)	BMI ≥30 vs.<25 kg/m^2	Oesophageal adenocarcinoma	2.73 (2.16, 3.46)	per 5 kg/m^2	1.13 (1.11, 1.16)
	WCRF report, revised 2018 (*86*)	16 cohort studies	per 5 kg/m^2 BMI	Oesophageal adenocarcinoma	1.51 (1.38, 1.65)	per 5 kg/m^2	1.51 (1.38, 1.65)

Consensus statements	Public health organization	Excerpt from statement	Citation
	WCRF/AICR, 2018 (*86*)	There is strong evidence that being overweight or obese increases the risk of adenocarcinoma of the oesophagus.	Continuous Update Project Expert Report 2018. Diet, nutrition, physical activity and oesophageal cancer. Last updated in 2018. Available from: https://www.wcrf.org/wp-content/uploads/2021/02/oesophageal-cancer-report.pdf
	IARC, 2016 (*87*)	There is sufficient evidence in humans for a cancer-preventive effect of absence of excess body fatness. Absence of excess body fatness prevents cancers of the colon and rectum, oesophagus (adenocarcinoma), stomach (gastric cardia), liver (hepatocellular carcinoma), gall bladder, pancreas, breast in postmenopausal women, endometrium, ovary, kidney (renal cell carcinoma), and thyroid, as well as meningioma and multiple myeloma. […] Absence of excess body fatness prevents cancers in humans (Group A).	IARC Handbooks of Cancer Prevention Volume 16. Absence of Excess Body Fatness. Available from: https://publications.iarc.fr/Book-And-Report-Series/Iarc-Handbooks-Of-Cancer-Prevention/Absence-Of-Excess-Body-Fatness-2018

L. Cigarette smoke and pancreatic cancer

Assumed causal evaluation as presented by Taubes in 1995: Indeterminate
Updated causal evaluation after review in 2021: Causal

Meta-analyses	First author, year	Number and designs of primary studies	Exposure	Outcome	Summary estimate (95% CI)	Dose-response assessment	Dose-response summary estimate (95% CI)
	Lynch, 2009 (*88*)	12 prospective cohorts, 1 case–control study	Current vs. never smokers	Pancreatic cancer	1.77 (1.38-2.26)	Pack-years:<br>≤10<br>>10-20<br>>20-30<br>>30-40<br>>40	 1.11 (0.85, 1.43)<br>1.32 (0.98, 1.77)<br>1.30 (0.97, 1.75)<br>1.49 (1.08, 2.03)<br>1.78 (1.35, 2.34)
	La Torre, 2009 (*89*)	6 cohort studies	Ever vs. never smokers	Pancreatic cancer	1.78 (1.64-1.92)		
	Zou, 2014 (*90*)	42 studies (30 retrospective, 12 prospective)	40 pack-years vs. non-smokers	Pancreatic cancer	1.9 (1.7-2.1)	Pack-years:<br>5<br>10<br>15<br>20<br>25<br>30<br>35<br>40	 1.2 (1.1, 1.2)<br>1.3 (1.2, 1.4)<br>1.5 (1.3, 1.6)<br>1.6 (1.4, 1.7)<br>1.7 (1.5, 1.9)<br>1.8 (1.6, 2.0)<br>1.8 (1.7, 2.0)<br>1.9 (1.7, 2.1)

Consensus statements	Public health organization	Excerpt from statement	Citation
	IARC, 2012 (*60*)	Tobacco smoking causes cancers of the lung, oral cavity, naso-, oro- and hypopharynx, nasal cavity and accesory sinuses, larynx, oesophagus, stomach, pancreas, colorectum, liver, kidney (body and pelvis), ureter, urinary bladder, uterine cervix and ovary (mucinous), and myeloid leukaemia. Tobacco smoking is carcinogenic to humans (Group 1).	IARC Monographs on the Evaluation of Carcinogenic Risks to Humans Volume 100 E. Available from: https://www.ncbi.nlm.nih.gov/books/NBK304391/

M. Lengthy occupational dioxin (TCDD) exposure and cancer

Assumed causal evaluation as presented by Taubes in 1995: Indeterminate
Updated causal evaluation after review in 2021: Causal

Meta-analyses	First author, year	Number and designs of primary studies	Exposure	Outcome	Summary estimate (95% CI)	Dose-response assessment	Dose-response summary estimate (95% CI)
	Leng, 2014 (*91*)	13 cohort studies	Exposure to TCDD	Prostate cancer mortality	1.26 (1.00, 1.57)[a]		
	Xu, 2016 (*92*)	3 cohort studies, 4 case–control studies	Highest vs. lowest categories of TCDD blood level	Incidence of all cancers	1.57 (1.21, 2.04)	Serum TEQ (toxicity equivalence factors) dose of 1000, 10000, 100000 ppt-years	SMRs: 110.67 (99.09, 122.26) 119.82 (105.79, 133.23) 167.68 (141.77, 194.21)

[a]pooled RR for TCDD exposure and prostate cancer incidence was 1.04 (95% CI: 0.85, 1.28)

Consensus statements	Public health organization	Excerpt from statement	Citation
	IARC, 2012 (*78*)	There is sufficient evidence in humans for the carcinogenicity of 2,3,7,8-tetrachlorodibenzo-para-dioxin. The strongest evidence in humans for the carcinogenicity of 2,3,7,8-tetrachlorodibenzo-para-dioxin is for all cancers combined. […] 2,3,7,8-Tetrachlorodibenzo-para-dioxin is carcinogenic to humans (Group 1).	IARC Monographs on the Evaluation of Carcinogenic Risks to Humans Volume 100 F. Available from: https://www.ncbi.nlm.nih.gov/books/NBK304391/

N. Alcohol and breast cancer

Assumed causal evaluation as presented by Taubes in 1995: Indeterminate
Updated causal evaluation after review in 2021: Causal

Meta-analyses	First author, year	Number and designs of primary studies	Exposure	Outcome	Summary estimate (95% CI)	Dose-response assessment	Dose-response summary estimate (95% CI)
	Bagnardi, 2015 (*66*)	118 studies (43 cohort, 75 case–control)	Heavy drinkers vs. non-drinkers	Breast cancer	1.61 (1.33, 1.94)	Non-drinkers<br>Light<br>Moderate<br>Heavy	1.0 (ref)<br>1.04 (1.01, 1.07)<br>1.23 (1.19, 1.28)<br>1.61 (1.33, 1.94)
	Jayesekara, 2016 (*67*)	16 articles (3 cohort, 13 case–control)	Highest vs. lowest average alcohol intake during lifetime/over time	Breast cancer	1.28 (1.07, 1.52)		
	Choi, 2017 (*68*)	25 cohort studies	Light drinking (≤1 drink/day) vs. none	Breast cancer	1.09 (1.06, 1.12)	None<br>≤0.5 drink/day<br>≤1 drink/day<br>1-2 drinks/day	1.0 (ref)<br>1.04 (1.01, 1.07)<br>1.09 (1.06, 1.12)<br>1.13 (1.11, 1.15)

Consensus statements	Public health organization	Excerpt from statement	Citation
	IARC, 2012 (*60*)	There is sufficient evidence in humans for the carcinogenicity of alcohol consumption. Alcohol consumption causes cancers of the oral cavity, pharynx, larynx, oesophagus, colorectum, liver (hepatocellular carcinoma) and female breast. […] Alcohol consumption is carcinogenic to humans (Group 1).	IARC Monographs on the Evaluation of Carcinogenic Risks to Humans Volume 100 E. Available from: https://www.ncbi.nlm.nih.gov/books/NBK304391/
	American Cancer Society, 2019 (*93*)	Drinking alcohol is clearly linked to an increased risk of breast cancer.	Breast Cancer Risk and Prevention. Last reviewed September 10, 2019. Available from: https://www.cancer.org/content/dam/CRC/PDF/Public/8578.00.pdf

O. Residential radon and lung cancer

Assumed causal evaluation as presented by Taubes in 1995: Indeterminate
Updated causal evaluation after review in 2021: Causal

Meta-analyses	First author, year	Number and designs of primary studies	Exposure	Outcome	Summary estimate (95% CI)	Dose-response assessment	Dose-response summary estimate (95% CI)
	Zhang, 2012 (*94*)	22 case–control (majority population-based, 3 hospital-based, 1 combined population and hospital subjects as controls)	Highest vs. lowest residential radon exposure	Lung cancer	1.29 (1.10, 1.51)	per 100 Bq/m^3	1.07 (1.04, 1.10)
	Duan, 2015 (*95*)	26 retrospective studies	200 Bq/m^3 vs. lowest radon exposure	Lung cancer	1.21 (1.14, 1.29)	non-linear dose response (threshold effect at 200 Bq/m^3)	
	Garzillo, 2017 (*96*)	25 case–control studies (18 population-based, 6 hospital-based, 1 both)	High vs. low indoor radon exposure	Lung cancer	1.19 (1.02, 1.39)		

Consensus statements	Public health organization	Excerpt from statement	Citation
	EPA, 2012 (*97*)	Radon is a cancer-causing, radioactive gas. Radon is estimated to cause many thousands of deaths each year. That's because when you breathe air containing radon, you can get lung cancer.	A Citizen's Guide to Radon: The Guide to Protecting Yourself and Your Family from Radon. Available from: https://www.epa.gov/sites/production/files/2016-02/documents/2012_a_citizens_guide_to_radon.pdf
	IARC, 2012 (*65*)	There is sufficient evidence in humans for the carcinogenicity of radon-222 and its decay products. Radon-222 and its decay products cause cancer of the lung. […] Radon-222 with its decay products are carcinogenic to humans (Group 1).	IARC Monographs on the Evaluation of Carcinogenic Risks to Humans Volume 100 D. Available from: https://www.ncbi.nlm.nih.gov/books/NBK304363/#a009.sec5

P. Eating red meat and colon cancer

Assumed causal evaluation as presented by Taubes in 1995: Indeterminate
Updated causal evaluation after review in 2021: Causal

	First author, year	Number and designs of primary studies	Exposure	Outcome	Summary estimate (95% CI)	Dose-response assessment	Dose-response summary estimate (95% CI)
Meta-analyses	Norat, 2002 (*98*)	15 case–control, 9 cohort studies	Highest quantile of red meat intake vs. lowest	Colorectal cancer	1.35 (1.21, 1.51)	Increase of 120 g/day of red meat	1.24 (1.08, 1.41)
	Pham, 2014 (*99*)	4 cohort studies, 4 case–control studies	Highest vs. lowest categories of red meat consumption	Colon cancer	1.21 (1.03, 1.43)		
	Vieira, 2017 (*100*)	11 prospective studies	100 g of red meat/day increment	Colon cancer	1.22 (1.06, 1.39)		

	Public health organization	Excerpt from statement	Citation
Consensus statements	IARC, 2018 (*101*)	There is limited evidence in humans for the carcinogenicity of consumption of red meat. Positive associations have been observed between consumption of red meat and cancers of the colorectum, pancreas, and prostate. […] Consumption of red meat is probably carcinogenic to humans (Group 2A).	IARC Monographs on the Evaluation of Carcinogenic Risks to Humans Volume 114. Available from: https://www.ncbi.nlm.nih.gov/books/NBK507971/
	WCRF/AICR, 2018 (*102*)	There is strong evidence that consuming red meat increases the risk of colorectal cancer. […] Consumption of red meat is probably a cause of colorectal cancer.	Continuous Update Project Expert Report 2018. Diet, nutrition, physical activity and colorectal cancer. Last updated in 2018. Available from: https://www.wcrf.org/wp-content/uploads/2021/02/Colorectal-cancer-report.pdf

Q. High birthweight and breast cancer

Assumed causal evaluation as presented by Taubes in 1995: Indeterminate
Updated causal evaluation after review in 2021: Causal

	First author, year	Number and designs of primary studies	Exposure	Outcome	Summary estimate (95% CI)	Dose-response assessment	Dose-response summary estimate (95% CI)
Meta-analyses	Park, 2008 (*103*)	8 case–control studies	Birthweight ≥4000 g vs. 2500 g -3000 g	Breast cancer	1.24 (1.04, 1.48)	<2500 g<br>2500 to 2999 g<br>3000 to 3499 g<br>3500 to 3999 g<br>≥4000 g	1.11 (0.90, 1.33)<br>1.0 (ref)<br>1.11 (0.99, 1.25)<br>1.15 (1.04, 1.26)<br>1.24 (1.04, 1.48)
	Xu, 2009 (*104*)	11 case–control, 7 cohort	Highest birthweight (>4000 g) vs. Lowest (<2500 g or 3000 g)	Breast cancer	1.20 (1.08, 1.34)	per 1 kg increase in birthweight	1.07 (1.02, 1.12)
	WCRF report, revised 2018 (*83*)	16 studies (including one pooled analysis of 8 cohort and 5 case–control studies)	Birthweight per 500 grams	Premenopausal breast cancer	1.05 (1.02, 1.09)	per 500 grams	1.05 (1.02, 1.09)

	Public health organization	Excerpt from statement	Citation
Consensus statements	WCRF/AICR, 2018 (*83*)	There is strong evidence that factors that lead to birthweight, or its consequences, increase the risk of premenopausal breast cancer. […] The factors that lead to greater birthweight, or its consequences, are probably a cause of premenopausal breast cancer.	Continuous Update Project Expert Report 2018. Diet, nutrition, physical activity and breast cancer. Last updated in 2018. Available from: https://www.wcrf.org/wp-content/uploads/2021/02/Breast-cancer-report.pdf
	Susan G. Komen, 2020 (*105*)	Women who had a higher birthweight have an increased risk of breast cancer compared to women who had a lower birthweight. This is most clearly seen in premenopausal (before menopause) women.	Birthweight and breast cancer risk. Last reviewed December 28, 2020. Available from: https://www.komen.org/breast-cancer/facts-statistics/research-studies/topics/birthweight-and-breast-cancer-risk/

R. Oral contraceptive use and breast cancer

Assumed causal evaluation as presented by Taubes in 1995: Indeterminate
Updated causal evaluation after review in 2021: Causal

	First author, year	Number and designs of primary studies	Exposure	Outcome	Summary estimate (95% CI)	Dose-response assessment	Dose-response summary estimate (95% CI)
Meta-analyses	Gierisch, 2013 (*106*)	44 studies (14 cohort, 29 case–control, 1 pooled analysis)	Users vs. non-users	Breast cancer	1.08 (1.00, 1.17)	Duration of use (months):<br>1-12<br>13-60<br>61-120<br>>120<br>Time since last oral contraceptive use (years):<br>0-5<br>5-10<br>10-20<br>>20 years	 <br>0.95 (0.83, 1.09)<br>1.03 (0.92, 1.15)<br>1.01 (0.90, 1.13)<br>1.04 (0.93, 1.17)<br><br><br><br>1.21 (1.04, 1.4)<br>1.17 (0.98, 1.38)<br>1.13 (0.97, 1.31)<br>1.02 (0.88, 1.18)
	Li, 2017 (*107*)	7 case–control studies	Ever >1 year vs. Never <1 year	Triple-negative Breast Cancer	1.21 (1.01, 1.46)		

	Public health organization	Excerpt from statement	Citation
Consensus statements	IARC, 2012 (*108*)	There is sufficient evidence in humans for the carcinogenicity of combined estrogen–progestogen oral contraceptives. Combined estrogen–progestogen oral contraceptives cause cancer of the breast, in-situ and invasive cancer of the uterine cervix, and cancer of the liver. […] Combined estrogen–progestogen oral contraceptives are carcinogenic to humans (Group 1).	IARC Monographs on the Evaluation of Carcinogenic Risks to Humans Volume 100A. Available from: https://www.ncbi.nlm.nih.gov/books/NBK304334/
	NCI, 2018 (*109*)	Data from observational studies cannot definitively establish that an exposure—in this case, oral contraceptives—causes (or prevents) cancer. […] Overall, however, these studies have provided consistent evidence that the risks of breast and cervical cancers are increased in women who use oral contraceptives, whereas the risks of endometrial, ovarian, and colorectal cancers are reduced.	Oral contraceptives and Cancer Risk. Last reviewed on February 22, 2018. Available from: https://www.cancer.gov/about-cancer/causes-prevention/risk/hormones/oral-contraceptives-fact-sheet#r4

S. Sun lamp use and skin melanoma

Assumed causal evaluation as presented by Taubes in 1995: Indeterminate
Updated causal evaluation after review in 2021: Causal

Meta-analyses	First author, year	Number and designs of primary studies	Exposure	Outcome	Summary estimate (95% CI)	Dose-response assessment	Dose-response summary estimate (95% CI)
	IARC, 2007 (*110*)	19 studies (1 cohort, 9 population-based case–control studies, 9 other case–control studies)	Ever vs. never use of sunbeds	Melanoma	1.15 (1.00, 1.31)	Sunbed use before 35 years old	1.75 (1.35, 2.26)
	Boniol, 2012 (*111*)	27 studies (18 cohort/population-based case–control, 9 other case–control studies)	Ever use vs. never use of sunbeds	Melanoma	1.20 (1.08, 1.34)	# of sunbed sessions/year<br>High use<br>1st use in youth	1.018 (0.998, 1.038)<br><br>1.42 (1.15, 1.74)<br>1.87 (1.41, 2.48)
	Colantonio, 2014 (*112*)	31 studies (12 population-based case–control, 15 hospital-based case–control, 2 prospective cohort, 1 nested case–control, 1 cross-sectional)	Ever vs. never used indoor tanning	Melanoma	1.16 (1.05, 1.28)	Duration of use (years):<br>Never<br>≤1<br>>1<br>Lifetime # of sessions:<br>Never<br>1-10<br>≥10	 1.0 (ref)<br>1.37 (1.06, 1.77)<br>1.61 (0.98, 2.67)<br><br>1.0 (ref)<br>1.07 (0.90, 1.26)<br>1.34 (1.05, 1.71)

Consensus statements	Public health organization	Excerpt from statement	Citation
	NCI, 2015 (*64*)	The sun, sunlamps, and tanning booths all give off ultraviolet (UV) radiation. Exposure to UV radiation causes early aging of the skin and damage that can lead to skin cancer.	Sunlight. Last reviewed May 1, 2020. Available from: https://www.cancer.gov/about-cancer/causes-prevention/risk/sunlight
	IARC, 2012 (*113*)	There is sufficient evidence in humans for the carcinogenicity of the use of UV-emitting tanning devices. UV-emitting tanning devices cause cutaneous malignant melanoma and ocular melanoma (observed in the choroid and the ciliary body of the eye). […] Use of UV-emitting tanning devices is carcinogenic to humans (Group 1).	IARC Monographs on the Evaluation of Carcinogenic Risks to Humans Volume 100 D. Available from: https://www.ncbi.nlm.nih.gov/books/NBK304366/#a006.sec5

T. Eating processed meat and colon cancer

Assumed causal evaluation as presented by Taubes in 1995: Indeterminate
Updated causal evaluation after review in 2021: Causal

Meta-analyses	First author, year	Number and designs of primary studies	Exposure	Outcome	Summary estimate (95% CI)	Dose-response assessment	Dose-response summary estimate (95% CI)
	Chan, 2011 (*114*)	11 studies	Highest vs. lowest processed meat intake	Colon cancer	1.19 (1.11, 1.29)	Per 50 grams of processed meat/day	1.24 (1.13, 1.35)
	Chiavarini, 2017 (*115*)	11 studies (7 case–control, 4 cohort)	Highest vs. lowest intake of MDM	Colorectal cancer	1.12 (1.06, 1.19)	No dose-response observed	
	Zhao, 2017 (*116*)	23 studies (11 case–control, 12 cohort)	Highest vs. lowest processed meat intake	Colon cancer	1.21 (1.13, 1.31)	Per 50 grams of processed meat/day	1.23 (1.11, 1.37)

MDM=”meat derived mutagenic activity”

Consensus statements	Public health organization	Excerpt from statement	Citation
	IARC, 2018 (*101*)	There is sufficient evidence in humans for the carcinogenicity of consumption of processed meat. Consumption of processed meat causes cancer of the colorectum. […] Consumption of processed meat is carcinogenic to humans (Group 1).	IARC Monographs on the Evaluation of Carcinogenic Risks to Humans Volume 114. Available from: https://www.ncbi.nlm.nih.gov/books/NBK507971/
	WCRF/AICR, 2018 (*102*)	There is strong evidence that consuming processed meat increases the risk of colorectal cancer. […] Consumption of processed meat is a convincing cause of colorectal cancer.	Continuous Update Project Expert Report 2018. Diet, nutrition, physical activity and colorectal cancer. Last updated in 2018. Available from: https://www.wcrf.org/wp-content/uploads/2021/02/Colorectal-cancer-report.pdf

U. Breastfeeding and brain cancer/leukemia in children

Assumed causal evaluation as presented by Taubes in 1995: Indeterminate
Updated causal evaluation after review in 2021: Causal

	First author, year	Number and designs of primary studies	Exposure	Outcome	Summary estimate (95% CI)	Dose-response assessment	Dose-response summary estimate (95% CI)
Meta-analyses	Kwan, 2004 (*117*)	14 case–control studies	Long-term breastfeeding (>6 months) vs. none/never	Acute lymphoblastic leukemia (ALL)	0.76 (0.68, 0.84)	None/never<br>Short-term breastfeeding (≤6 months)<br>Long-term breastfeeding (>6 months)	<u>ALL:</u><br>1.0 (ref)<br>0.88 (0.80, 0.96)<br>0.76 (0.68, 0.84)<br><u>AML:</u><br>1.0 (ref)<br>0.90 (0.80, 1.02)<br>0.85 (0.73, 0.98)
	Martin, 2005 (*118*)	26 studies (24 case–control, 2 cohort/nested case–control studies)	Ever vs. never breastfed	Childhood central nervous system (CNS) cancers	0.95 (0.80, 1.13)	<u>Breastfeeding duration:</u><br>Never breastfed<br><6 months<br>>6 months	<u>Leukemia:</u><br>1.0 (ref)<br>0.89 (0.76, 1.03)<br>0.72 (0.58, 0.90)<br><u>CNS cancers</u>:<br>1.0 (ref)<br>0.94 (0.77, 1.16)<br>0.81 (0.63, 1.02)
	Amitay, 2015 (*119*)	15 case–control studies	Ever vs. never breastfed	Childhood leukemia	0.91 (0.80, 1.03)	Any breastfeeding for 6 months or longer vs. no or shorter breastfeeding	0.80 (0.72, 0.90)

	Public health organization	Excerpt from statement	Citation
Consensus statements	NCI, 2021 (*120*)	[…] being breastfed and having been exposed to routine childhood infections are both associated with a lowered risk of developing childhood leukemia.	Cancer in Children and Adolescents. Last updated on April 20, 2021. Available from: https://www.cancer.gov/types/childhood-cancers/child-adolescent-cancers-fact-sheet#r25
	U.S. Department of Health & Human Services,	Research shows that breastfed babies have lower risk of: Asthma, leukemia (during childhood), obesity (during childhood), ear infections, eczema (atopic	Making the decision to breastfeed. Last updated on March 14, 2019. Available from:

	Office on Women's Health (*121*)	dermatitis), diarrhea and vomiting, lower respiratory infections, necrotizing enterocolitis, sudden infant death syndrome (SIDS), type 2 diabetes	https://www.womenshealth.gov/breastfeeding/making-decision-breastfeed

V. High-alcohol mouthwash and mouth cancer

Assumed causal evaluation as presented by Taubes in 1995: Indeterminate
Updated causal evaluation after review in 2021: Indeterminate

	First author, year	Number and designs of primary studies	Exposure	Outcome	Summary estimate (95% CI)	Dose-response assessment	Dose-response summary estimate (95% CI)
Meta-analyses	Gandini, 2012 (*122*)	3 case–control studies	Use of alcohol-containing mouthwash (25% alcohol content)	Oral cancer	1.16 (0.44, 3.08)		
	Boffetta, 2016 (*123*)	Pooled analysis of 12 case–control studies	Ever-use of mouthwash vs. never use	Oral cavity cancer	1.11 (1.00, 1.23)	Duration of use (years)<br>0 (non-users)<br>1-15<br>16-35<br>36+	1.0 (ref)<br>0.95 (0.78, 1.16)<br>1.15 (0.96, 1.39)<br>1.28 (1.06, 1.56)

	Public health organization	Excerpt from statement	Citation
Consensus statements	FDA, 2003 (*15*)	Based on the studies reviewed, the Subcommittee concludes that the available data do not support a causal relationship between the use of alcohol-containing mouthrinses and oral cancer. […] However, because some studies did report a relationship between the use of high alcohol-content mouthrinses and pharyngeal cancer, the Subcommittee agrees that further studies should be conducted to determine the relationship between high alcohol-content mouthrinses and oral/pharyngeal cancers.	Oral Health Care Drug Products for Over-the-Counter Human Use; Antigingivitis/Antiplaque Drug Products; Establishment of a Monograph. Publication date: May 29, 2003. Available from: https://www.federalregister.gov/documents/2003/05/29/03-12783/oral-health-care-drug-products-for-over-the-counter-human-use-antigingivitisantiplaque-drug-products
	American Cancer Society, 2021 (*124*)	Some studies have suggested that mouthwash with a high alcohol content might be linked to a higher risk of oral and oropharyngeal cancers. But recent research has questioned these results. Studying this possible link is complicated by the fact that smokers and frequent drinkers (who already have an increased risk of these cancers) are more likely to use mouthwash than people who neither smoke nor drink.	Risk Factors for Oral Cavity and Oropharyngeal Cancers. Last reviewed on March 23, 2021. Available from: https://www.cancer.org/cancer/oral-cavity-and-oropharyngeal-cancer/causes-risks-prevention/risk-factors.html

W. Electromagnetic fields (EMF) and brain cancer/leukemia in children

Assumed causal evaluation as presented by Taubes in 1995: Indeterminate
Updated causal evaluation after review in 2021: Indeterminate

	First author, year	Number and designs of primary studies	Exposure	Outcome	Summary estimate (95% CI)	Dose-response assessment	Dose-response summary estimate (95% CI)
Meta-analyses	Wartenberg, 2001 (*125*)	14 studies (11 case–control, 3 cohort/nested case–control)	Calculated and measured magnetic fields (cut-point as close to 0.2 μT used)	Childhood leukemia	1.32 (1.09, 1.59)		
	Kheifets, 2010 (*126*)	Pooled analysis of 7 matched case–control studies	≥0.3 μT vs.<0.1 μT	Childhood leukemia	1.44 (0.88, 2.36)	per 0.2 μT increase<br><br>Distance from nearest power line:<br>>200 m<br>>100-200 m<br>>50-100 m<br>≤50 m	1.11 (0.98, 1.26)<br><br><br><br>1.0 (ref)<br>1.20 (0.90, 1.59)<br>1.30 (0.89, 1.91)<br>1.59 (1.02, 2.50)
	Kheifets, 2010 (*127*)	Pooled analysis of 10 case–control studies	≥0.4 μT vs.<0.1 μT	Childhood brain tumors	1.14 (0.61, 2.13)	per 0.2 μT increase	0.96 (0.86, 1.07)

	Public health organization	Excerpt from statement	Citation
Consensus statements	IARC, 2002 (*13*)	There is limited evidence in humans for the carcinogenicity of extremely low-frequency magnetic fields in relation to childhood leukaemia. […] Extremely low-frequency magnetic fields are possibly carcinogenic to humans (Group 2B).	IARC Monographs on the Evaluation of Carcinogenic Risks to Humans Volume 80. Available from: https://www.ncbi.nlm.nih.gov/books/NBK390731/
	WHO, 2007 (*128*)	Consistent epidemiological evidence suggests that chronic low intensity ELF magnetic field exposure is associated with an increased risk of childhood leukaemia. However, the evidence for a causal relationship is limited, therefore exposure limits based upon epidemiological evidence are not recommended, but some precautionary measures are warranted.	Extremely Low Frequency Fields Environmental Health Criteria Monograph No.238. ISBN**:** 9789241572385. Published March 13, 2007. Available from: https://www.who.int/publications/i/item/9789241572385

	NCI, 2019 (*129*)	No consistent evidence for an association between any source of non-ionizing EMF and cancer [in childhood] has been found.	Electromagnetic Fields and Cancer. Last reviewed on January 3, 2019. Available from: https://www.cancer.gov/about-cancer/causes-prevention/risk/radiation/electromagnetic-fields-fact-sheet#r27

X. Traffic density and brain cancer/leukemia in children

Assumed causal evaluation as presented by Taubes in 1995: Indeterminate
Updated causal evaluation after review in 2021: Indeterminate

	First author, year	Number and designs of primary studies	Exposure	Outcome	Summary estimate (95% CI)	Dose-response assessment	Dose-response summary estimate (95% CI)
Meta-analyses	Boothe, 2014 (*130*)	8 case–control studies	Highest vs. lowest residential traffic exposure (variety of exposure measures were used across studies)	Childhood leukemia	1.39 (1.03, 1.88)		
	Carlos-Wallace, 2016 (*131*)	11 cohort/ case–control studies	Traffic density (highest exposure category)	Childhood leukemia	1.25 (0.96, 1.62)		
	Filippini, 2019 (*132*)	29 studies (26 case–control, 3 cohort studies)	Highest vs. lowest traffic density	Childhood leukemia	1.09 (1.00, 1.20)	Vehicles per day in the street closest to the child's residence<br><br>Benzene (µg/m^3)<br><br>NO_2 (µg/m3)	Vehicles per day: "Showed little association except at the highest exposure levels where a small and statistically imprecise excess risk emerged"<br><br>Benzene: linear dose-response for AML<br><br>NO_2: statistically unstable

	Public health organization	Excerpt from statement	Citation
Consensus statements	IARC, 2018 (*133*)	The Working Group noted a consistent association between exposure to benzene and AML for children, and coherence with findings for adult AML and benzene exposure, but could not rule out chance, bias, and confounding as alternative explanations.	IARC Monographs on the Evaluation of Carcinogenic Risks to Humans, No. 120. Available from: https://www.ncbi.nlm.nih.gov/books/NBK550159/

	CDC, 2020 (*14*)	Research suggests that we can take steps today to protect the health of babies and young children, and reduce their chances of getting cancer in the future [such as] reducing exposure to traffic-related air pollution.	Cancer Prevention During Early Life. Last reviewed on July 13, 2020. Available from: https://www.cdc.gov/cancer/dcpc/prevention/childhood.htm

Y. High cholesterol diet and rectal cancer

Assumed causal evaluation as presented by Taubes in 1995: Indeterminate
Updated causal evaluation after review in 2021: Indeterminate

Meta-analyses	First author, year	Number and designs of primary studies	Exposure	Outcome	Summary estimate (95% CI)	Dose-response assessment	Dose-response summary estimate (95% CI)
	Howe, 1997 (*134*)	10 case–control studies	per 437 mg of cholesterol/day	Rectal cancer	1.53 (1.26, 1.87)		
	Liu, 2011 (*135*)	7 prospective cohort studies	Highest vs. lowest level of cholesterol intake	Colorectal cancer	1.10 (0.92, 1.32)		

Consensus statements	Public health organization	Excerpt from statement	Citation
	WCRF/AICR, 2015 (*136*)	Research has not shown a link between dietary cholesterol and cancer risk.	The Cancer-Cholesterol Connection. Last reviewed on February 18, 2015. Available from: https://www.aicr.org/news/the-cancer-cholesterol-connection/
	WCRF/AICR, 2018 (*102*)	Evidence for the following exposures [for the risk of colorectal cancer] have been judged as 'limited – no conclusion': cereals (grains) and their products, potatoes, poultry, shellfish and other seafood, fatty acid composition, cholesterol, dietary n-3 fatty acid from fish, legumes, non-dairy sources of calcium, sugar (sucrose), coffee, tea, caffeine, carbohydrate, total fat, starch, glycaemic load, vitamin A, vitamin B6, methionine, beta-carotene, alpha-carotene, lycopene, retinol, energy intake, meal frequency.	Continuous Update Project Expert Report 2018. Diet, nutrition, physical activity and colorectal cancer. Last updated in 2018. Available from: https://www.wcrf.org/wp-content/uploads/2021/02/Colorectal-cancer-report.pdf

Z. Douching and Cervical Cancer

Assumed causal evaluation as presented by Taubes in 1995: Indeterminate
Updated causal evaluation after review in 2021: Indeterminate

Meta-analyses	First author, year	Number and designs of primary studies	Exposure	Outcome	Summary estimate (95% CI)	Dose-response assessment (n studies)	Dose-response summary estimate (95% CI)
	Zhang, 1997 (*137*)	6 population-based case–control studies	Douching vs. not douching	Cervical cancer	1.25 (0.99, 1.59)	≥1 time/week vs. none (n=5)	1.86 (1.29, 2.68)
						>20 years vs. unknown ref (n=3)	1.47 (0.83, 2.61)

Consensus statements	Public health organization	Excerpt from statement	Citation
	American Public Health Association (*138*)	Studies have shown links between the practice of vaginal douching (intravaginal cleansing with a liquid solution) and several adverse health outcomes. Some of these adverse health outcomes include pelvic inflammatory disease, bacterial vaginosis, cervical cancer [...]... There is some conflict, however, among studies with respect to those adverse health outcomes that have a causal link or an observed association. For example, those women with certain risk factors (i.e., multiple sexual partners, poverty, certain races, and lower educational level) are also at a greater risk of sexually transmitted infection, bacterial vaginosis, and pelvic inflammatory disease. This situation makes it difficult to determine causality, because women may douche as a result of infection-related symptoms rather than as a part of their normal hygienic practice.	Vaginal Douching and Adverse Health Outcomes. Nov 6, 2007. Policy number: 20074<br><br>https://www.apha.org/policies-and-advocacy/public-health-policy-statements/policy-database/2014/07/29/13/06/vaginal-douching-and-adverse-health-outcomes
	Office on Women's Health, U.S. Department of Health and Human Services (*139*)	Doctors recommend that you do not douche. Douching can lead to many health problems, including problems getting pregnant. Douching is also linked to vaginal infections and sexually transmitted infections (STIs).	Douching. Last reviewed: April 1, 2019. https://www.womenshealth.gov/a-z-topics/douching#14

AA. Occupational stress and colorectal cancer

Assumed causal evaluation as presented by Taubes in 1995: Indeterminate
Updated causal evaluation after review in 2021: Indeterminate

Meta-analyses	First author, year	Number and designs of primary studies	Exposure	Outcome	Summary estimate (95% CI)	Dose-response assessment	Dose-response summary estimate (95% CI)
	Heikkila, 2013 (*140*)	12 prospective cohort studies	High demand and low control vs. no strain (all other categories combined)	Colorectal cancer	1.16 (0.90, 1.48)	Job strain quadrants:<br>1. High strain (high demand, low control),<br>2. Active job (high demands, high control)<br>3. Passive job (low demands, low controls)<br>4. Low strain job (ref; low demands, high control)	0.96 (0.73, 1.27)<br>0.84 (0.67, 1.06)<br>0.75 (0.58, 0.97)<br>1.0 (ref)
	Yang, 2019 (*141*)	14 studies (12 cohort, 2 case–control)	High work stress vs. no strain group	Colorectal cancer	1.36 (1.16, 1.59)		

Consensus statements	Public health organization	Excerpt from statement	Citation
	NCI, 2012 (*142*)	Although stress can cause a number of physical health problems, the evidence that it can cause cancer is weak.	Psychological Stress and Cancer. Last reviewed on December 10, 2012. Available from: https://www.cancer.gov/about-cancer/coping/feelings/stress-fact-sheet

BB. Smoking and fatal breast cancer

Assumed causal evaluation as presented by Taubes in 1995: Indeterminate
Updated causal evaluation after review in 2021: Indeterminate

Meta-analyses	First author, year	Number and designs of primary studies	Exposure	Outcome	Summary estimate (95% CI)	Dose-response assessment	Dose-response summary estimate (95% CI)
	Bérubé, 2014 (*143*)	10 cohort studies	Smokers at diagnosis vs. never smokers	Breast cancer-specific mortality	1.33 (1.12, 1.58)		
	Wang, 2016 (*144*)	11 prospective cohort studies	Per 10 cigarettes/day	Breast cancer-specific mortality	1.10 (1.04, 1.16)	Per 10 cigarettes/day<br>Per 10 pack-years,<br>Per 10 years smoking increase	1.10 (1.04, 1.16)<br>1.09 (1.06, 1.12)<br>1.10 (1.06, 1.14)
	Duan, 2017 (*145*)	12 prospective cohort studies	Current smoking within 1 year before or after breast cancer diagnosis vs. never smokers	Breast cancer-specific mortality	1.30 (1.16, 1.45)	Never smoker<br>Former smoker<br>Current smoker	1.0 (ref)<br>0.95 (0.90, 1.02)<br>1.30 (1.16, 1.45)

Consensus statements	Public health organization	Excerpt from statement	Citation
	Susan G. Komen (*146*)	There's growing evidence smoking decreases survival for women diagnosed with breast cancer.	Healthy Lifestyle for Breast Cancer Survivors. Last reviewed: unknown. Available from: https://www.komen.org/breast-cancer/survivorship/healthy-lifestyle/#Not-smoking

CC. Hair dyes and myeloma

Assumed causal evaluation as presented by Taubes in 1995: Indeterminate
Updated causal evaluation after review in 2021: Indeterminate

Meta-analyses	First author, year	Number and designs of primary studies	Exposure	Outcome	Summary estimate (95% CI)	Dose-response assessment	Dose-response summary estimate (95% CI)
	Takkouche, 2005 (*147*)	6 studies (4 case–control, 2 cohort)	Any dye use vs. none	Multiple myeloma	1.14 (0.86, 1.52)	Permanent dye use	1.10 (0.62, 1.95)

Consensus statements	Public health organization	Excerpt from statement	Citation
	IARC, 2010 (*148*)	There is inadequate evidence in humans for the carcinogenicity of personal use of hair colourants. […] Personal use of hair colourants is not classifiable as to its carcinogenicity to humans (Group 3).	IARC Monographs on the Evaluation of Carcinogenic Risks to Humans, No. 99. Available from: https://www.ncbi.nlm.nih.gov/books/NBK385419/
	American Cancer Society, 2020 (*149*)	Studies looking at a possible link between personal hair dye use and the risk of blood-related cancers such as leukemia and lymphoma have had mixed results. For example, some studies have found an increased risk of certain types of non-Hodgkin lymphoma (but not others) in women who use hair dyes, especially if they began use before 1980 and/or use darker colors. The same types of results have been found in some studies of leukemia risk. However, other studies have not found an increased risk. If there is an effect of hair dye use on blood-related cancers, it is likely to be small.	Hair Dyes. Last reviewed on September 8, 2020. Available from: https://www.cancer.org/cancer/cancer-causes/hair-dyes.html

DD. Chlorinated tap water and bladder cancer

Assumed causal evaluation as presented by Taubes in 1995: Indeterminate
Updated causal evaluation after review in 2021: Indeterminate

Meta-analyses	First author, year	Number and designs of primary studies	Exposure	Outcome	Summary estimate (95% CI)	Dose-response assessment	Dose-response summary estimate (95% CI)
	Villanueva, 2003 (*150*)	8 studies (6 case–control studies and 2 cohort studies)	Ever consumption of chlorinated drinking water vs. low/no exposure	Bladder cancer	1.2 (1.1, 1.3)	Years of exposure:<br>20<br>40<br>60	 1.13 (1.08, 1.20)<br>1.27 (1.17, 1.43)<br>1.43 (1.27, 1.72)
	Costet, 2011 (*151*)	6 case–control studies	Total trihalomethane level (µg/l):<br>>5 vs. 0-5 (ref)	Bladder cancer	1.31 (1.16, 1.49)[a]	Total trihalomethane level (µg/l):<br>0-5<br>>5-25<br>>25-50<br>>50	 1 (ref)[a]<br>1.25 (1.06, 1.47)[a]<br>1.35 (1.09, 1.66)[a]<br>1.51 (1.26, 1.82)[a]
	Bai, 2014 (*152*)	21 studies (17 case–control, 4 cohort studies)	Highest vs. lowest total fluid consumption	Bladder cancer	1.06 (0.88, 1.27)	Number of beverages:<br>≥5<br><5	 1.19 (0.97, 1.46)<br>0.60 (0.34, 1.06)

[a]Results for men only; results for men and women combined were not presented

Consensus statements	Public health organization	Excerpt from statement	Citation
	EPA, 2000 (*153*)	Several human studies have investigated the relationship between exposure to chlorinated drinking water and cancer. These studies were not designed to assess whether chlorine itself causes cancer, but whether trihalomethanes or other organic compounds occurring in drinking water as a result of chlorination are associated with an increased risk of cancer. These studies show an association between bladder and rectal cancer and chlorination byproducts in drinking water. […] EPA has not classified chlorine for carcinogenicity.	Chlorine. Last reviewed in January 2000. Available from: https://www.epa.gov/sites/production/files/2016-09/documents/chlorine.pdf
	IARC, 2013 (*154*)	By-products of chlorination, and specifically trihalomethanes, were first detected in the early 1970s. Four trihalomethanes (chloroform [IARC Group 2B], bromodichloromethane [IARC Group 2B], dibromochloromethane [IARC Group 3] and bromoform [IARC Group 3]), together with nine bromine- and chlorine-based haloacetic acids, are the main by-products of chlorination on a weight basis. The chlorine-bromine speciation depends on the bromine content of the raw water. Trihalomethanes and haloacetic acids are regulated in the European Union, the USA and other countries.	IARC Monographs on the Evaluation of Carcinogenic Risks to Humans, No. 101. Available from: https://www.ncbi.nlm.nih.gov/books/NBK373192/

EE. Eating yogurt and ovarian cancer

Assumed causal evaluation as presented by Taubes in 1995: Indeterminate
Updated causal evaluation after review in 2021: Indeterminate

Meta-analyses	First author, year	Number and designs of primary studies	Exposure	Outcome	Summary estimate (95% CI)	Dose-response assessment	Dose-response summary estimate (95% CI)
	Larsson, 2006 (*155*)	7 case–control studies, 2 prospective cohorts	Highest vs. lowest categories of yogurt consumption	Ovarian Cancer	1.13 (0.96, 1.33)		
	Genkinger, 2006 (*156*)	12 prospective cohort studies	≥114 g/day of yogurt vs. 0 g/day	Ovarian Cancer	1.04 (0.86, 1.24)	227 g/day increment	0.91 (0.77, 1.07)
	Liu, 2015 (*157*)	10 case–control studies	High vs. low yogurt consumption	Ovarian Cancer	1.12 (0.86, 1.45)		

Consensus statements	Public health organization	Excerpt from statement	Citation
	WCRF/AICR, 2018 (*158*)	Evidence for the following exposures previously judged as 'limited-no conclusion' in the SER, remain unchanged after updated the analyses with new data identified in the Ovarian Cancer SLR 2013: fruits; poultry; fish; eggs; milk and dairy products; coffee; tea; dietary fibre; lactose; total fat; alcohol; folate; vitamin A; vitamin C; vitamin E; abdominal fatness and physical activity.	Continuous Update Project Expert Report 2018. Diet, nutrition, physical activity and ovarian cancer. Last updated in 2018. Available from: https://www.wcrf.org/wp-content/uploads/2021/02/ovarian-cancer-report.pdf

FF. Hair dyes and lymphoma

Assumed causal evaluation as presented by Taubes in 1995: Indeterminate
Updated causal evaluation after review in 2021: Indeterminate

	First author, year	Number and designs of primary studies	Exposure	Outcome	Summary estimate (95% CI)	Dose-response assessment	Dose-response summary estimate (95% CI)
Meta-analyses	Takkouche, 2005 (*147*)	14 studies (2 cohort, 9 population-based case–control, 3 hospital-based case–control)	Any dye use vs. none	Non-Hodgkin's Lymphoma	1.23 (1.07, 1.42)	Permanent dye use<br><br>Intensive exposure (>200 lifetime exposures to hair dyes)	1.13 (1.01, 1.26)<br><br>1.07 (0.90, 1.28)
	Zhang, 2008 (*159*)	4 case–control studies	Any use of hair dye (ever vs. never)	Non-Hodgkin Lymphoma	1.0 (0.9, 1.2)	Duration of use (never, <8, 8-19, 20+ years) for FL and CLL/SLL<br><br>No. of applications/year (never, <5, 5-8,9+) for FL and CLL/SLL<br><br>Total no. of applications (never, <31, 31-138, 139+) for FL and CLL/SLL	FL: 1.0 (ref), 2.3 (0.9, 1.6), 1.3 (1.0, 1.7), 1.5 (1.1, 1.9), p-trend=0.01<br>CLL/SLL: 1.0 (ref), 1.2 (0.8, 1.6), 1.3 (0.9, 1.8), 1.3 (1.0, 1.8), p-trend=0.07<br><br>FL: 1.0 (ref), 1.3 (1.0, 1.7), 1.2 (0.9, 1.6), 1.3 (1.0, 1.8), p-trend=0.05<br>CLL/SLL: 1.0 (ref), 1.1 (0.7, 1.5), 1.2 (0.8, 1.), 1.6 (1.1, 2.1), p-trend <0.01<br><br>FL: 1.0 (ref), 1.2 (0.9, 1.6), 1.2 (0.9, 1.6), 1.4 (1.1, 1.9), p-trend=0.02<br>CLL/SLL: 1.0 (ref), 1.1 (0.8, 1.6), 1.2 (0.8, 1.7). 1.5 (1.1, 2.1), p-trend=0.01
	Qin, 2019 (*160*)	16 studies (3 cohort, 13 case–control)	Hair colorant users vs. nonusers	Non-Hodgkin's Lymphoma	1.14 (1.01, 1.29)	Duration of use<br>Non-use<br><10 years<br>10-20 years<br>20+ years	 1.0 (ref)<br>1.19 (0.90, 1.88)<br>1.20 (1.02, 1.95)<br>1.34 (1.04, 1.92)

FL=follicular lymphoma; CLL/SLL=chronic lymphocyte leukemia/small lymphocytic lymphoma

	Public health organization	Excerpt from statement	Citation
Consensus statements	IARC, 2010 (*148*)	There is inadequate evidence in humans for the carcinogenicity of personal use of hair colourants. […] Personal use of hair colourants is not classifiable as to its carcinogenicity to humans (Group 3).	IARC Monographs on the Evaluation of Carcinogenic Risks to Humans, No. 99. Available from: https://www.ncbi.nlm.nih.gov/books/NBK385419/
	American Cancer Society, 2020 (*149*)	Studies looking at a possible link between personal hair dye use and the risk of blood-related cancers such as leukemia and lymphoma have had mixed results. For example, some studies have found an increased risk of certain types of non-Hodgkin lymphoma (but not others) in women who use hair dyes, especially if they began use before 1980 and/or use darker colors. The same types of results have been found in some studies of leukemia risk. However, other studies have not found an increased risk. If there is an effect of hair dye use on blood-related cancers, it is likely to be small.	Hair Dyes. Last reviewed on September 8, 2020. Available from: https://www.cancer.org/cancer/cancer-causes/hair-dyes.html
	NCI, 2016 (*161*)	A number of studies have investigated the relationship between the personal use of hair dyes and the risk of NHL, with conflicting results.	Hair Dyes and Cancer Risk. Last reviewed on August 18, 2016. Available from https://www.cancer.gov/about-cancer/causes-prevention/risk/myths/hair-dyes-fact-sheet

GG. Electromagnetic fields (EMF) and brain cancer

Assumed causal evaluation as presented by Taubes in 1995: Indeterminate
Updated causal evaluation after review in 2021: Indeterminate

	First author, year	Number and designs of primary studies	Exposure	Outcome	Summary estimate (95% CI)	Dose-response assessment	Dose-response summary estimate (95% CI)
Meta-analyses	Mezei, 2008 (*162*)	5 studies for distance to power lines (1 hospital-based case–control, 2 nested case–control, 1 case–control, 1 population-based case–control)	<50 m vs. 100+ m residential distance to power lines	Childhood brain cancer	0.88 (0.57, 1.37)		
	Kheifets, 2008 (*163*)	47 studies (mix of cohort and case–control, unknown total # of each)	Highest vs. lowest occupational EMF exposure	Adult brain cancer	1.14 (1.07, 1.22)	<25 ("background")<br>25-75 ("low")<br>75-90 ("medium")<br>90+ ("high") percentiles	No dose-response

	Public health organization	Excerpt from statement	Citation
Consensus statements	IARC, 2002 (*13*)	There is limited evidence in humans for the carcinogenicity of extremely low-frequency magnetic fields in relation to childhood leukaemia. There is inadequate evidence in humans for the carcinogenicity of extremely low-frequency magnetic fields in relation to all other cancers. There is inadequate evidence in humans for the carcinogenicity of static electric or magnetic fields and extremely low-frequency electric fields. […] Extremely low-frequency magnetic fields are possibly carcinogenic to humans (Group 2B). Static electric and magnetic fields and extremely low-frequency electric fields are not classifiable as to their carcinogenicity to humans (Group 3).	IARC Monographs on the Evaluation of Carcinogenic Risks to Humans Volume 80. Available from: https://www.ncbi.nlm.nih.gov/books/NBK390731/
	WHO, 2007 (*128*)	In the case of adult brain cancer and leukaemia, the new studies published after the IARC monograph do not change the conclusion that the overall evidence for an association between ELF and the risk of these diseases remains inadequate.	Extremely Low Frequency Fields Environmental Health Criteria Monograph No.238. ISBN**:** 9789241572385. Published March 13, 2007. Available from: https://www.who.int/publications/i/item/9789241572385
	NCI, 2019 (*129*)	Several studies conducted in the 1980s and early 1990s reported that people who worked in some electrical occupations that exposed them to ELF radiation (such as power station	Electromagnetic Fields and Cancer. Last reviewed on January 3, 2019. Available from:

		operators and telephone line workers) had higher-than-expected rates of some types of cancer, particularly leukemia, brain tumors, and male breast cancer. Most of the results were based on participants’ job titles and not on actual measurements of their exposures. More recent studies, including some that considered exposure measurements as well as job titles, have generally not shown an increasing risk of leukemia, brain tumors, or female breast cancer with increasing exposure to magnetic fields at work.	https://www.cancer.gov/about-cancer/causes-prevention/risk/radiation/electromagnetic-fields-fact-sheet#r27

HH. Hair dyes and leukemia

Assumed causal evaluation as presented by Taubes in 1995: Indeterminate
Updated causal evaluation after review in 2021: Indeterminate

Meta-analyses	First author, year	Number and designs of primary studies	Exposure	Outcome	Summary estimate (95% CI)	Dose-response assessment	Dose-response summary estimate (95% CI)
	Takkouche, 2005 (*147*)	16 studies (13 case–control, 3 cohort)	Any dye use vs. none	All leukemia types	1.12 (0.94, 1.34)	Permanent dye use	1.13 (0.97, 1.31)
	Towle, 2017 (*164*)	20 studies (16 case–control, 4 cohort)	Ever use of personal hair dye vs. no use	All leukemia types	1.09 (0.97, 1.22)	Years of hair dye use:<br>Nonusers<br>≤10 years<br>≥15 years	 1.0 (ref)<br>0.94 (0.80, 1.11)<br>1.35 (1.13, 1.62)

Consensus statements	Public health organization	Excerpt from statement	Citation
	IARC, 2010 (*148*)	There is inadequate evidence in humans for the carcinogenicity of personal use of hair colourants. […] Personal use of hair colourants is not classifiable as to its carcinogenicity to humans (Group 3).	IARC Monographs on the Evaluation of Carcinogenic Risks to Humans, No. 99. Available from: https://www.ncbi.nlm.nih.gov/books/NBK385419/
	American Cancer Society, 2020 (*149*)	Studies looking at a possible link between personal hair dye use and the risk of blood-related cancers such as leukemia and lymphoma have had mixed results. For example, some studies have found an increased risk of certain types of non-Hodgkin lymphoma (but not others) in women who use hair dyes, especially if they began use before 1980 and/or use darker colors. The same types of results have been found in some studies of leukemia risk. However, other studies have not found an increased risk. If there is an effect of hair dye use on blood-related cancers, it is likely to be small.	Hair Dyes. Last reviewed on September 8, 2020. Available from: https://www.cancer.org/cancer/cancer-causes/hair-dyes.html
	NCI, 2016 (*161*)	Studies of the association between personal hair dye use and the risk of leukemia have had conflicting results.	Hair Dyes and Cancer Risk. Last reviewed on August 18, 2016. Available from https://www.cancer.gov/about-cancer/causes-prevention/risk/myths

II. Smoking and breast cancer

Assumed causal evaluation as presented by Taubes in 1995: Indeterminate
Updated causal evaluation after review in 2021: Indeterminate

Meta-analyses	First author, year	Number and designs of primary studies	Exposure	Outcome	Summary estimate (95% CI)	Dose-response assessment	Dose-response summary estimate (95% CI)
	Ambrosone, 2008 (*165*)	13 studies (9 population-based case–control, 4 nested case–control)	Ever active smoking vs. never active smoking	Breast cancer	1.17 (1.07, 1.27)	Never<br>Low pack-years<br>High pack-years[a]	1.0 (ref)<br>1.14 (1.03, 1.25)<br>1.26 (1.12, 1.43)
	Gaudet, 2013 (*166*)	15 cohorts	Current vs. never smokers	Breast cancer	1.12 (1.08, 1.16)	Never smoker<br>Former smoker<br>Current smoker	1.0 (ref)<br>1.09 (1.04, 1.15)<br>1.12 (1.08, 1.16)
	Macacu, 2015 (*167*)	27 prospective studies	Current active vs. never smokers	Breast cancer	1.13 (1.09, 1.17)	Never<br>Ever passive<br>Ever active<br>Former active<br>Current active	1.0 (ref)<br>1.07 (1.02, 1.13)<br>1.10 (1.09, 1.12)<br>1.09 (1.06, 1.12)<br>1.13 (1.09, 1.17)

[a]The cut-point between low and high pack-years was 20 pack-years for 12 studies and 15 pack-years for 1 study

Consensus statements	Public health organization	Excerpt from statement	Citation
	IARC, 2012 (*60*)	A positive association has been observed between tobacco smoking and cancer of the female breast. […] Observed associations are weaker and less consistent for breast cancer than for other tobacco-related cancers. Furthermore, several methodological considerations could either obscure a small increase in risk caused by tobacco smoking, or alternatively introduce a spurious association where no causal relationship exists.	IARC Monographs on the Evaluation of Carcinogenic Risks to Humans Volume 100 E. Available from: https://www.ncbi.nlm.nih.gov/books/NBK304391/
	American Cancer Society, 2020 (*168*)	Some studies have found that heavy smoking over a long time might be linked to a slightly higher risk of breast cancer. In some studies, the risk has been highest in certain groups, such as women who started smoking before they had their first child. The 2014 US Surgeon General's report on smoking concluded that there is "suggestive but not sufficient" evidence that smoking increases the risk of breast cancer.	Factors with Unclear Effects on Breast Cancer Risk. Last reviewed on June 9, 2020. Available from: https://www.cancer.org/cancer/breast-cancer/risk-and-prevention/factors-with-unclear-effects-on-breast-cancer-risk.html

JJ. Diet high in saturated fat and lung cancer (among non-smokers)

Assumed causal evaluation as presented by Taubes in 1995: Indeterminate
Updated causal evaluation after review in 2021: Indeterminate

	First author, year	Number and designs of primary studies	Exposure	Outcome	Summary estimate (95% CI)	Dose-response assessment	Dose-response summary estimate (95% CI)
Meta-analyses	Yang, 2017 (*169*)	Pooled analysis of 10 prospective cohort studies	Highest vs. lowest quintile of saturated fat intake (among never smokers)	Lung cancer	1.03 (0.84, 1.26)	Quintiles of saturated fat intake (Q1-Q5)	All:<br>1.0 (ref)<br>1.02 (0.97, 1.08)<br>1.05 (0.98, 1.11)<br>1.08 (1.01, 1.16)<br>1.14 (1.07, 1.22)<br>Never smokers:<br>1.0 (ref)<br>1.08 (0.93, 1.25)<br>0.96 (0.81, 1.14)<br>0.95 (0.70, 1.28)<br>1.03 (0.84, 1.26)<br>Ever smokers:<br>1.0 (ref)<br>1.01 (0.95, 1.07)<br>1.06 (0.99, 1.13)<br>1.09 (1.02, 1.17)<br>1.15 (1.07, 1.24)

	Public health organization	Excerpt from statement	Citation
Consensus statements	WCRF/AICR, 2018 (*170*)	Evidence for the following exposures [for the risk of lung cancer] have been judged as 'limited – no conclusion': cereals (grains) and their products, starchy tubers; vegetables (never smokers); fruits (never smokers);dietary fibre; pulses (legumes); citrus fruits; poultry; fish; eggs; milk and dairy products; total meat; total fat; animal fats; plant oils; soft drinks; coffee; tea; carbohydrate; protein; vitamin A; thiamin; riboflavin; niacin; vitamin B6; folate; foods containing vitamin C (former and never smokers); vitamin E; selenium; calcium; copper; iron; zinc; beta-carotene supplements (never and former smokers); alpha-carotene; lycopene; beta-cryptoxanthin, lutein, and zeaxanthin; foods containing isoflavones (current and	Continuous Update Project Expert Report 2018. Diet, nutrition, physical activity and lung cancer. Last updated in 2018. Available from: https://www.wcrf.org/wp-content/uploads/2021/02/lung-cancer-report.pdf

	former smokers); plasma hydroxyvitamin D; vitamin C supplements; retinol supplements; multivitamin supplements; patterns of diet; body fatness; energy intake; height.

KK. Electromagnetic fields (EMF) and leukemia

Assumed causal evaluation as presented by Taubes in 1995: Indeterminate
Updated causal evaluation after review in 2021: Indeterminate

Meta-analyses	First author, year	Number and designs of primary studies	Exposure	Outcome	Summary estimate (95% CI)	Dose-response assessment	Dose-response summary estimate (95% CI)
	Kheifets, 2008 (*163*)	56 studies (mix of cohort and case–control, unknown total # of each)	Highest vs. lowest occupational EMF exposure	Adult leukemia	1.16 (1.11, 1.22)	<25 ("background")<br>25-75 ("low")<br>75-90 ("medium")<br>90+ ("high") percentiles	No dose-response

Consensus statements	Public health organization	Excerpt from statement	Citation
	IARC, 2002 (*13*)	There is limited evidence in humans for the carcinogenicity of extremely low-frequency magnetic fields in relation to childhood leukaemia. There is inadequate evidence in humans for the carcinogenicity of extremely low-frequency magnetic fields in relation to all other cancers. There is inadequate evidence in humans for the carcinogenicity of static electric or magnetic fields and extremely low-frequency electric fields. […] Extremely low-frequency magnetic fields are possibly carcinogenic to humans (Group 2B). Static electric and magnetic fields and extremely low-frequency electric fields are not classifiable as to their carcinogenicity to humans (Group 3).	IARC Monographs on the Evaluation of Carcinogenic Risks to Humans Volume 80. Available from: https://www.ncbi.nlm.nih.gov/books/NBK390731/
	WHO, 2007 (*128*)	In the case of adult brain cancer and leukaemia, the new studies published after the IARC monograph do not change the conclusion that the overall evidence for an association between ELF and the risk of these diseases remains inadequate.	Extremely Low Frequency Fields Environmental Health Criteria Monograph No.238. ISBN**:** 9789241572385. Published March 13, 2007. Available from: https://www.who.int/publications/i/item/9789241572385
	NCI, 2019 (*129*)	Several studies conducted in the 1980s and early 1990s reported that people who worked in some electrical occupations that exposed them to ELF radiation (such as power station operators and telephone line workers) had higher-than-expected rates of some types of cancer, particularly leukemia, brain tumors, and male breast cancer. Most of the results were based on participants' job titles and not on actual measurements of their exposures. More recent studies, including some that considered exposure measurements as well as job titles, have generally not shown an increasing risk of leukemia, brain tumors, or female breast cancer with increasing exposure to magnetic fields at work.	Electromagnetic Fields and Cancer. Last reviewed on January 3, 2019. Available from: https://www.cancer.gov/about-cancer/causes-prevention/risk/radiation/electromagnetic-fields-fact-sheet#r27

LL. Fat intake and breast cancer

Assumed causal evaluation as presented by Taubes in 1995: Indeterminate
Updated causal evaluation after review in 2021: Indeterminate

	First author, year	Number and designs of primary studies	Exposure	Outcome	Summary estimate (95% CI)	Dose-response assessment	Dose-response summary estimate (95% CI)
Meta-analyses	Turner, 2011 (*171*)	52 studies (25 cohort, 27 case–control)	Highest vs. lowest quartile of total fat	Breast cancer	1.01 (0.99, 1.03)		
	Yang, 2014 (*172*)	6 prospective nested case–control, 5 cohort studies	Highest vs. lowest quartile of ratio of n-3/n-6 PUFAs	Breast cancer	0.90 (0.82, 0.99)	per 1/10 increment of ratio in diet	0.94 (0.90, 0.99); p for linear trend=0.012
	Cao, 2016 (*173*)	24 prospective studies	Highest vs. lowest category of dietary total fat intake	Breast cancer	1.10 (1.02, 1.19)	increment of 10 g/day of dietary total fat	1.03 (1.01, 1.05)

PUFAs=Polyunsaturated fatty acids

	Public health organization	Excerpt from statement	Citation
Consensus statements	WCRF/AICR, 2018 (*83*)	The following exposures, for which evidence also was previously too limited to draw conclusions in the Second Expert Report and not updated as part of the CUP, remain ‘limited-no conclusion’: cereal grains and their products, potatoes, pulses (legumes), eggs, fats and oils, vegetable fat, fatty acid composition, trans fatty acids, cholesterol, sugar (sucrose), other sugars, sugary foods and drinks, starch, protein, vitamin A, riboflavin, vitamin B6, vitamin B12, vitamin C, vitamin E, iron, selenium, dichlorodiphenyldichloroethylene (DDE), dichlorodiphenyltrichloroethane (DDT), dieldrin, hexachlorobenzene, hexachlorocyclohexane, trans-nonachlor, polychlorinated biphenyls, culturally defined diets, birth length, being breastfed. In addition, evidence for the following exposures, for which no judgement was made in the Second Expert Report, is too limited to draw any conclusions: acrylamide, glycaemic load, saturated fatty acids, monounsaturated fatty acids, polyunsaturated fatty acids, calcium supplements, phytoestrogens, sedentary behavior.	Continuous Update Project Expert Report 2018. Diet, nutrition, physical activity and breast cancer. Last updated in 2018. Available from: https://www.wcrf.org/wp-content/uploads/2021/02/Breast-cancer-report.pdf
	American Cancer Society, 2020 (*168*)	Studies of women in the United States have not found a consistent link between high-fat diets and getting breast cancer	Factors with Unclear Effects on Breast Cancer Risk. Last revised on June 9, 2020. Available from: https://www.cancer.org/cancer/breast-

cancer/risk-and-prevention/factors-with-unclear-effects-on-breast-cancer-risk.html

MM. Maternal smoking and brain cancer/leukemia in children

Assumed causal evaluation as presented by Taubes in 1995: Indeterminate
Updated causal evaluation after review in 2021: Indeterminate

	First author, year	Number and designs of primary studies	Exposure	Outcome	Summary estimate (95% CI)	Dose-response assessment	Dose-response summary estimate (95% CI)
Meta-analyses	Zhou, 2014 (*174*)	18 studies (17 case–control, 1 cohort)	Maternal smoking during pregnancy	Childhood Acute Lymphoblastic Leukemia	0.99 (0.93, 1.06)		
	Huang, 2014 (*175*)	16 studies (14 case–control, 2 cohort)	Maternal smoking during pregnancy	Childhood Brain Tumors	0.96 (0.86, 1.07)	Amount of maternal smoking during pregnancy (increase in 10 cigarettes per day)<br><br>Amount of maternal smoking before pregnancy (increase in 10 cigarettes per day)	0.98 (0.92, 1.04) p=0.506 for linear trend<br><br>0.95 (0.89, 1.02) p=0.179 for linear trend
	Metayer, 2016 (*176*)	11 case–control studies	Ever maternal smoking vs. never	Childhood Acute Myeloid Leukemia	1.02 (0.88, 1.18)	Prenatal maternal smoking (per 5 cigarettes/day)	0.99 (0.98, 1.00)

	Public health organization	Excerpt from statement	Citation
Consensus statements	IARC, 2012 (*60*)	The body of evidence suggests a consistent association of leukaemia (and lymphoma) with paternal smoking preconception and with combined parental smoking, with risk ratios ranging from 1.5 to 4.0. Maternal tobacco smoking during pregnancy generally showed modest increases in risk, or null or inverse relationships. The combined effects of preconception and post-conception exposures to tobacco smoke were highly significant. […] Overall these studies do not show an association with either paternal smoking, largely preconception, or maternal smoking prior, during or after pregnancy, or by CNS types, gliomas and primitive neuroectodermal tumours.	IARC Monographs on the Evaluation of Carcinogenic Risks to Humans Volume 100 E. Available from: https://www.ncbi.nlm.nih.gov/books/NBK304391/

	IARC, 2021 (*177*)	Limited evidence in humans for tobacco smoking and childhood leukaemia (in smokers' children)	List of classifications by cancers sites with sufficient or limited evidence in humans, IARC Monographs Volumes 1–129. Last updated on March 26, 2021. Available from: https://monographs.iarc.who.int/wp-content/uploads/2019/07/Classifications_by_cancer_site.pdf

NN. Eating red meat and breast cancer

Assumed causal evaluation as presented by Taubes in 1995: Indeterminate
Updated causal evaluation after review in 2021: Indeterminate

Meta-analyses	First author, year	Number and designs of primary studies	Exposure	Outcome	Summary estimate (95% CI)	Dose-response assessment	Dose-response summary estimate (95% CI)
	Wu, 2016 (*178*)	8 cohort studies	Highest vs. lowest total red meat intake	Breast cancer	1.05 (0.95, 1.16)	per 120 g of total meat intake/day	1.07 (1.01, 1.14)
	Anderson, 2018 (*179*)	10 cohort studies	High red meat intake	Breast cancer	1.03 (0.99, 1.08)		
	Farvid, 2018 (*180*)	13 cohort, 3 nested case–control, 2 clinical trials	Highest vs. lowest total red meat intake	Breast cancer	1.06 (0.99, 1.14)		

Consensus statements	Public health organization	Excerpt from statement	Citation
	IARC, 2018 (*101*)	About 10 cohort studies (with a total of about 20 000 cases of cancer of the breast), and a consortium of eight prospective cohort studies (> 7000 cases of cancer of the breast), assessed risk of cancer of the breast in relation to consumption of red meat (which may or may not have included processed meat) in North America and Europe. Four of these cohort studies found a statistically significant positive association between risk of cancer of the breast and consumption of red meat or red and processed meat combined.	IARC Monographs on the Evaluation of Carcinogenic Risks to Humans Volume 114. Available from: https://www.ncbi.nlm.nih.gov/books/NBK507971/
	WCRF/AICR, 2018 (*83*)	Evidence considered 'limited-no conclusion' for red and processed meats for premenopausal and postmenopausal breast cancer.	Continuous Update Project Expert Report 2018. Diet, nutrition, physical activity and breast cancer. Last updated in 2018. Available from: https://www.wcrf.org/wp-content/uploads/2021/02/Breast-cancer-report.pdf

OO. Electromagnetic Fields (EMF) and breast cancer

Assumed causal evaluation as presented by Taubes in 1995: Indeterminate
Updated causal evaluation after review in 2021: Indeterminate

	First author, year	Number and designs of primary studies	Exposure	Outcome	Summary estimate (95% CI)	Dose-response assessment	Dose-response summary estimate (95% CI)
Meta-analyses	Chen, 2010 (*181*)	15 case–control studies	ELF-EMF exposure (cut-points closest to 0.2 μT were most common)	Breast cancer (female)	0.99 (0.90, 1.09)		
	Chen, 2013 (*182*)	23 case–control studies	ELF-EMF exposure	Breast cancer (female)	1.07 (1.02, 1.13)		
	Zhang, 2016 (*183*)	23 case–control studies	ELF-EMF exposure	Breast cancer	All: 1.07 (1.00, 1.15) Premenopausal: 1.57 (0.95, 2.59) Postmenopausal: 1.00 (0.88, 1.14)		

Abbreviations: ELF-EMF, Extremely low-frequency electromagnetic fields

	Public health organization	Excerpt from statement	Citation
Consensus statements	IARC, 2002 (*13*)	There is limited evidence in humans for the carcinogenicity of extremely low-frequency magnetic fields in relation to childhood leukaemia. There is inadequate evidence in humans for the carcinogenicity of extremely low-frequency magnetic fields in relation to all other cancers. There is inadequate evidence in humans for the carcinogenicity of static electric or magnetic fields and extremely low-frequency electric fields. […] Extremely low-frequency magnetic fields are possibly carcinogenic to humans (Group 2B). Static electric and magnetic fields and extremely low-frequency electric fields are not classifiable as to their carcinogenicity to humans (Group 3).	IARC Monographs on the Evaluation of Carcinogenic Risks to Humans Volume 80. Available from: https://www.ncbi.nlm.nih.gov/books/NBK390731/
	WHO, 2007 (*128*)	Subsequent to the IARC monograph a number of reports have been published concerning the risk of female breast cancer in adults associated with ELF magnetic field exposure. These studies are larger than the previous ones and less susceptible to bias, and overall are negative. With these studies, the evidence for an association between ELF exposure and the risk of breast cancer is weakened considerably and does not support an association of this kind.	Extremely Low Frequency Fields Environmental Health Criteria Monograph No.238. ISBN**:** 9789241572385. Published March 13, 2007. Available from: https://www.who.int/publications/i/item/9789241572385
	NCI, 2019 (*129*)	Several studies conducted in the 1980s and early 1990s reported that people who worked in some electrical occupations that exposed them to ELF radiation (such as power station	Electromagnetic Fields and Cancer. Last reviewed on January 3, 2019. Available from:

		operators and telephone line workers) had higher-than-expected rates of some types of cancer, particularly leukemia, brain tumors, and male breast cancer. Most of the results were based on participants' job titles and not on actual measurements of their exposures. More recent studies, including some that considered exposure measurements as well as job titles, have generally not shown an increasing risk of leukemia, brain tumors, or female breast cancer with increasing exposure to magnetic fields at work.	https://www.cancer.gov/about-cancer/causes-prevention/risk/radiation/electromagnetic-fields-fact-sheet#r27
	Susan G. Komen, 2019 (*184*)	Regular exposure to EMF does not appear to increase the risk of breast cancer.	Electromagnetic Fields and Breast Cancer Risk. Updated Sept. 19, 2019. Available from: https://ww5.komen.org/BreastCancer/Table26Electromagneticfieldsandbreastcancerrisk.html

PP. Coffee and heart disease

Assumed causal evaluation as presented by Taubes in 1995: Indeterminate
Updated causal evaluation after review in 2021: Indeterminate

	First author, year	Number and designs of primary studies	Exposure	Outcome	Summary estimate (95% CI)	Dose-response assessment	Dose-response summary estimate (95% CI)
Meta-analyses	Ding, 2014 (*185*)	36 prospective studies (34 cohort, 1 case–cohort, 1 nested case–control)	3rd highest (median: 1.5 cups/day) vs. lowest (median: 0 cups/day)	Cardiovascular disease	0.89 (0.84, 0.94)	RRs estimated from cubic spline model:<br>None<br>1 cup/day<br>2 cups/day<br>3 cups/day<br>4 cups/day<br>5 cups/day<br>6 cups/day<br>7 cups/day	 <br>1.0 (ref)<br>0.95 (0.93, 0.97)<br>0.92 (0.88, 0.95)<br>0.89 (0.85, 0.93)<br>0.88 (0.83, 0.93)<br>0.89 (0.83, 0.95)<br>0.91 (0.84, 0.99)<br>0.93 (0.85, 1.03)
	Larsson, 2015 (*186*)	6 prospective studies	Highest vs. lowest category of coffee intake	Atrial fibrillation	0.96 (0.84, 1.08)	2 cups/day increment	0.99 (0.94, 1.03)
	Caldeira, 2013 (*187*)	7 studies (6 cohort, 1 case–control)	Exposure to caffeine vs. non-consumers/lowest quintile of intake	Atrial fibrillation	0.92 (0.82, 1.04)	Caffeine exposure:<br>Low<br>Moderate<br>High	 0.85 (0.78, 0.92)<br>0.97 (0.81, 1.16)<br>0.97 (0.82, 1.13)

	Public health organization	Excerpt from statement	Citation
Consensus statements	American Heart Association (*188*)	Many studies have been done to see if there's a direct link between caffeine, coffee drinking and coronary heart disease. The results are conflicting. This may be due to the way the studies were done and confounding dietary factors. However, moderate coffee drinking (1–2 cups per day) doesn't seem to be harmful.	Caffeine and Heart Disease. Last reviewed: April 17, 2014 http://www.heart.org/en/healthy-living/healthy-eating/eat-smart/nutrition-basics/caffeine-and-heart-disease
	2015 Dietary Guidelines Advisory Committee to the Secretaries of the U.S. Departments of	Strong and consistent evidence shows that consumption of coffee within the moderate range (3 to 5 cups per day or up to 400 mg/d caffeine) is not associated with increased risk of major chronic diseases, such as cardiovascular disease (CVD) and cancer and premature death in healthy adults. DGAC Grade: Strong	Dietary Guidelines Advisory Committee. 2015. Scientific Report of the 2015 Dietary Guidelines Advisory Committee: Advisory Report to the Secretary of Health and Human Services and the Secretary of Agriculture.

	Health and Human Services and Agriculture (*189*)	Consistent observational evidence indicates that moderate coffee consumption is associated with reduced risk of type 2 diabetes and cardiovascular disease in healthy adults. [...] DGAC Grade: Moderate	U.S. Department of Agriculture, Agricultural Research Service, Washington, DC. https://health.gov/our-work/nutrition-physical-activity/dietary-guidelines/previous-dietary-guidelines/2015/advisory-report

QQ. Olive oil and breast cancer

Assumed causal evaluation as presented by Taubes in 1995: Indeterminate
Updated causal evaluation after review in 2021: Indeterminate

Meta-analyses	First author, year	Number and designs of primary studies	Exposure	Outcome	Summary estimate (95% CI)	Dose-response assessment	Dose-response summary estimate (95% CI)
	Pelucchi, 2011 (*190*)	7 studies (6 case–control, 1 cohort)	Highest vs. lowest level of olive oil consumption	Breast cancer	0.62 (0.44, 0.88)		
	Xin, 2015 (*191*)	3 prospective cohorts, 9 retrospective case–control studies	Highest vs. lowest level of olive oil consumption	Breast cancer	0.74 (0.60, 0.92)		

Consensus statements	Public health organization	Excerpt from statement	Citation
	WCRF/AICR, 2018 (*83*)	The following exposures, for which evidence also was previously too limited to draw conclusions in the Second Expert Report and not updated as part of the CUP, remain ‘limited-no conclusion’: cereal grains and their products, potatoes, pulses (legumes), eggs, fats and oils, vegetable fat, fatty acid composition, trans fatty acids, cholesterol, sugar (sucrose), other sugars, sugary foods and drinks, starch, protein, vitamin A, riboflavin, vitamin B6, vitamin B12, vitamin C, vitamin E, iron, selenium, dichlorodiphenyldichloroethylene (DDE), dichlorodiphenyltrichloroethane (DDT), dieldrin, hexachlorobenzene, hexachlorocyclohexane, trans-nonachlor, polychlorinated biphenyls, culturally defined diets, birth length, being breastfed. In addition, evidence for the following exposures, for which no judgement was made in the Second Expert Report, is too limited to draw any conclusions: acrylamide, glycaemic load, saturated fatty acids, monounsaturated fatty acids, polyunsaturated fatty acids, calcium supplements, phytoestrogens, sedentary behavior.	Continuous Update Project Expert Report 2018. Diet, nutrition, physical activity and breast cancer. Last updated in 2018. Available from: https://www.wcrf.org/wp-content/uploads/2021/02/Breast-cancer-report.pdf

RR. Coffee and pancreatic cancer

Assumed causal evaluation as presented by Taubes in 1995: Indeterminate
Updated causal evaluation after review in 2021: Non-causal

Meta-analyses	First author, year	Number and designs of primary studies	Exposure	Outcome	Summary estimate (95% CI)	Dose-response assessment	Dose-response summary estimate (95% CI)
	Yu, 2011 (*192*)	14 cohort studies	High vs. none/lowest coffee consumption	Pancreatic Cancer	0.82 (0.69, 0.95)	1 cup/day increase	0.96 (0.90, 1.02)
	Turati, 2012 (*193*)	17 cohort and 37 case–control	High vs. lowest coffee consumption	Pancreatic Cancer	1.13 (0.99, 1.29)	1 cup/day increase	1.03 (0.99, 1.06)
	Nie, 2016 (*194*)	21 prospective cohort studies	High vs. lowest coffee consumption	Pancreatic Cancer	0.99 (0.81, 1.21)	1 cup/day increase	1.01 (unknown)

Consensus statements	Public health organization	Excerpt from statement	Citation
	IARC, 2018 (*195*)	There is evidence suggesting lack of carcinogenicity of drinking coffee in humans for cancers of the pancreas, liver, female breast, uterine endometrium, and prostate. […] Drinking coffee is not classifiable as to its carcinogenicity to humans (Group 3).	IARC Monographs on the Evaluation of Carcinogenic Risks to Humans Volume 116. Available from: https://pubmed.ncbi.nlm.nih.gov/31310458/
	WCRF/AICR, 2018 (*196*)	For coffee and risk of pancreatic cancer, the evidence for an association was considered to be limited and no conclusion was possible.	Continuous Update Project Expert Report 2018. Diet, nutrition, physical activity and pancreatic cancer. Last updated in 2018. Available from: https://www.wcrf.org/wp-content/uploads/2021/02/pancreatic-cancer-report.pdf

SS. Vasectomy and prostate cancer

Assumed causal evaluation as presented by Taubes in 1995: Indeterminate
Updated causal evaluation after review in 2021: Non-causal

	First author, year	Number and designs of primary studies	Exposure	Outcome	Summary estimate (95% CI)	Dose-response assessment	Dose-response summary estimate (95% CI)
Meta-analyses	Shang, 2015 (*197*)	10 cohort studies	Vasectomy vs. no vasectomy	Prostate cancer	1.11 (0.98, 1.27)		
	Bhindi, 2017 (*198*)	7 cohort studies at low risk of bias	Vasectomy vs. no vasectomy	Prostate cancer	1.05 (1.02, 1.09)		
	Wu, 2018 (*199*)	14 cohort studies	Vasectomy vs. no vasectomy	Prostate cancer	1.07 (0.99, 1.16)		

	Public health organization	Excerpt from statement	Citation
Consensus statements	American Urological Association, 2015 (*200*)	Clinicians do not need to routinely discuss prostate cancer, coronary heart disease, stroke, hypertension, dementia or testicular cancer in pre-vasectomy counseling of patients because vasectomy is not a risk factor for these conditions. Standard (Evidence Strength: Grade B[a])	Vasectomy Guideline (2015). Sharlip ID, Belker AM, Honig S et al: Vasectomy: AUA guideline. J Urol 2012; 188: 2482. Available from: https://www.auanet.org//guidelines/guidelines/vasectomy-guideline
	American Cancer Society, 2020 (*201*)	Some studies have suggested that men who have had a vasectomy (minor surgery to make men infertile) have a slightly increased risk for prostate cancer, but other studies have not found this. Research on this possible link is still under way.	Prostate Cancer Risk Factors. Last reviewed on June 9, 2020. Available from: https://www.cancer.org/cancer/prostate-cancer/causes-risks-prevention/risk-factors.html

[a]The AUA categorizes body of evidence strength (ES) as Grade A (well conducted RCTs or exceptionally strong observational studies), Grade B (RCTs with some weaknesses of procedure or generalizability or generally strong observational studies) or Grade C (observational studies that are inconsistent, have small sample sizes, or have other problems that potentially confound interpretation of data).

TT. Breast self-examination (BSE) and breast cancer mortality

Assumed causal evaluation as presented by Taubes in 1995: Indeterminate
Updated causal evaluation after review in 2021: Non-causal

Meta-analyses	First author, year	Number and designs of primary studies	Exposure	Outcome	Summary estimate (95% CI)	Dose-response assessment	Dose-response summary estimate (95% CI)
	Hackshaw, 2003 (*202*)	3 clinical trials (1 non-randomized, 2 randomized)	Women taught BSE vs. not	Breast cancer mortality	1.01 (0.92, 1.12)		
	Kösters, 2003 (*203*)	2 population-based randomized trials	BSE vs. no BSE	Breast cancer mortality	1.05 (0.90, 1.24)		

Consensus statements	Public health organization	Excerpt from statement	Citation
	American College of Obstetricians and Gynecologists, 2019 (*204*)	Breast self-examination is not recommended in average-risk women because there is a risk of harm from false-positive test results and a lack of evidence of benefit.	Breast Cancer Risk Assessment and Screening in Average-Risk Women. Last reaffirmed in 2019. Available from: https://www.acog.org/clinical/clinical-guidance/practice-bulletin/articles/2017/07/breast-cancer-risk-assessment-and-screening-in-average-risk-women
	American Cancer Society, 2021 (*205*)	Research has not shown a clear benefit of regular physical breast exams done by either a health professional (clinical breast exams) or by women themselves (breast self-exams).	American Cancer Society Recommendations for the Early Detection of Breast Cancer. Last revised on April 22, 2021. Available from: https://www.cancer.org/cancer/breast-cancer/screening-tests-and-early-detection/american-cancer-society-recommendations-for-the-early-detection-of-breast-cancer.html
	NCI, 2021 (*206*)	Breast self-exam has been shown to have no mortality benefit.	Breast Cancer Screening (PDQ®)–Health Professional Version. Last updated on May 7, 2021. Available from: https://www.cancer.gov/types/breast/hp/breast-screening-pdq

UU. Abortion and breast cancer

Assumed causal evaluation as presented by Taubes in 1995: Indeterminate
Updated causal evaluation after review in 2021: Non-causal

	First author, year	Number and designs of primary studies	Exposure	Outcome	Summary estimate (95% CI)	Dose-response assessment	Dose-response summary estimate (95% CI)
Meta-analyses	Collaborative Group on Hormonal Factors in Breast Cancer, 2004 (*207*)	13 prospective studies	Women with a prospective record of having had one or more pregnancies that ended as an induced abortion vs. women with no such record	Breast cancer	0.93 (0.89, 0.96)	≥2 abortions vs. 1	0.96 (SE: 0.046)
	Huang, 2014 (*208*)	2 cohort studies	History of ≥1 induced abortions vs. not	Breast cancer	1.00 (0.80, 1.21)		
	Guo, 2015 (*209*)	14 studies (10 cohort, 4 registry-linkage studies)	Ever vs. never induced abortion	Breast cancer	1.00 (0.94, 1.05)	History of 1 induced abortion<br>History of ≥2 induced abortions	1.0 (0.91, 1.10)<br>0.99 (0.75, 1.24)

	Public health organization	Excerpt from statement	Citation
Consensus statements	Susan G. Komen Foundation, 2020 (*210*)	Research clearly shows abortion (also called induced abortion) does not increase the risk of breast cancer.	Abortion and breast cancer risk. Last updated on December 28, 2020. Available from: https://www.komen.org/breast-cancer/facts-statistics/research-studies/topics/abortion-and-breast-cancer-risk/
	American College of Obstetricians and Gynecologists, 2019 (*211*)	More rigorous recent studies demonstrate no causal relationship between induced abortion and a subsequent increase in breast cancer risk.	Induced Abortion and Breast Cancer Risk. Reaffirmed 2019. Available from: https://www.acog.org/clinical/clinical-guidance/committee-opinion/articles/2009/06/induced-abortion-and-breast-cancer-risk

VV. Dichlorodiphenyltrichloroethane (DDT) and breast cancer

Assumed causal evaluation as presented by Taubes in 1995: Indeterminate
Updated causal evaluation after review in 2021: Non-causal

Meta-analyses	First author, year	Number and designs of primary studies	Exposure	Outcome	Summary estimate (95% CI)	Dose-response assessment	Dose-response summary estimate (95% CI)
	López-Cervantes, 2004 (*212*)	22 studies (9 prospective nested case–control, 6 population-based case–control, 7 hospital-based)	Body burden levels of DDE (ng/g serum lipid bases using midpoint levels for each category)	Breast cancer	0.97 (0.87, 1.09)	Midpoint levels for each category	0.97 (0.87, 1.09)
	Ingber, 2013 (*213*)	38 studies for DDE (27 case–control, 11 nested case–control)<br>18 studies for DDT (12 case–control, 6 nested case–control)	Highest vs. lowest DDT levels	Breast cancer	1.02 (0.92, 1.13)		
	Park, 2014 (*214*)	35 studies (10 prospective nested case-contol studies, 16 hospital-based case-contol studies, and 11 population-based case-contol studies)	DDE exposure	Breast cancer	1.03 (0.95, 1.12)		

Consensus statements	Public health organization	Excerpt from statement	Citation
	IARC, 2018 (*215*)	No association overall was found between *p′*,p-DDE or *p,p′*-DDT levels and breast cancer. Stratification by hormone-receptor status of the breast tumour, or menopausal status, did not modify the results. Several meta-analyses on *p,p′*-DDE exposure was that the available studies supported the view that DDE is not associated with an increased risk of breast cancer in humans. However, the potential influence of age at exposure to DDT in relation to risk of breast cancer remains of interest, as suggested by two studies that reported an increased risk of breast cancer in women highly exposed to DDT early in life.	IARC Monographs on the Evaluation of Carcinogenic Risks to Humans Volume 113. Available from: https://www.ncbi.nlm.nih.gov/books/NBK507424/
	EPA, 2013 (*216*)	There is considerable interest in this outcome because of animal studies and the estrogenic activities of pesticides such as DDT, DDE, endosulfan and atrazine. Though atrazine is not a direct mimicker of estrogen, in some models it induces aromatase formation, which converts testosterone to estradiol. This effect is not consistent in all cell	Recognition and management of pesticide poisonings. Sixth edition. Available from: https://www.epa.gov/sites/production/files/2015-01/documents/rmpp_6thed_final_lowresopt.pdf

lines or animal models. Despite the evidence that estrogen is a promoter of breast cancer, the role of these pesticides in breast cancer remains unclear at this time.

WW. Saccharine and bladder cancer

Assumed causal evaluation as presented by Taubes in 1995: Non-causal
Updated causal evaluation after review in 2021: Non-causal

Meta-analyses	First author, year	Number and designs of primary studies	Exposure	Outcome	Summary estimate (95% CI)	Dose-response assessment	Dose-response summary estimate (95% CI)
	Elcock, 1993 (*217*)	14 case–control studies	Artificial sweeteners	Bladder cancer	0.98 (0.92, 1.04)		

Consensus statements	Public health organization	Excerpt from statement	Citation
	IARC, 1999 (*218*)	In making its evaluation, the Working Group concluded that sodium saccharin produces urothelial bladder tumours in rats by a non-DNA-reactive mechanism that involves the formation of a urinary calcium phosphate-containing precipitate, cytotoxicity and enhanced cell proliferation. This mechanism is not relevant to humans because of critical interspecies differences in urine composition. Saccharin and its salts are not classifiable as to their carcinogenicity to humans (Group 3).	Saccharin and Its Salts (Group 3). Last updated on September 30, 1999. Available from: http://www.inchem.org/documents/iarc/vol73/73-19.html
	NCI, 2016 (*219*)	Questions about artificial sweeteners and cancer arose when early studies showed that cyclamate in combination with saccharin caused bladder cancer in laboratory animals. However, results from subsequent carcinogenicity studies (studies that examine whether a substance can cause cancer) of these sweeteners have not provided clear evidence of an association with cancer in humans. Similarly, studies of other FDA-approved sweeteners have not demonstrated clear evidence of an association with cancer in humans.	Artificial Sweeteners and Cancer. Last reviewed on August 10, 2016. Available from: https://www.cancer.gov/about-cancer/causes-prevention/risk/diet/artificial-sweeteners-fact-sheet#is-there-an-association-between-artificial-sweeteners-and-cancer

REFERENCES AND NOTES

1. G. Taubes, Epidemiology faces its limits. *Science* **269**, 164–169 (1995).

2. W. Willett, S. Greenland, B. MacMahon, D. Trichopoulos, K. Rothman, D. Thomas, M. Thun, N. Weiss, The discipline of epidemiology. *Science* **269**, 1325–1326 (1995).

3. D. Trichopoulos, The discipline of epidemiology. *Science* **269**, 1326 (1995).

4. D. Trichopoulos, The future of epidemiology. *BMJ* **313**, 436–437 (1996).

5. A. J. Saah, The discipline of epidemiology. *Science* **269**, 1327 (1995).

6. A. H. Smith, Depicting epidemiology. *Science* **270**, 1743–1744 (1995).

7. E. L. Wynder, Invited commentary: Response to science article, "Epidemiology faces its limits". *Am. J. Epidemiol.* **143**, 747–748 (1996).

8. A. W. Harzing, "Publish or perish" (2016); https://harzing.com/resources/publish-or-perish.

9. A. Hubbard, J. Trostle, I. Cangemi, J. N. S. Eisenberg, Countering the curse of dimensionality: Exploring data-generating mechanisms through participant observation and mechanistic modeling. *Epidemiology* **30**, 609–614 (2019).

10. S. Jukola, On the evidentiary standards for nutrition advice. *Stud. Hist. Philos. Biol. Biomed. Sci.* **73**, 1–9 (2019).

11. T. E. Hardwicke, J. P. A. Ioannidis, Petitions in scientific argumentation: Dissecting the request to retire statistical significance. *Eur. J. Clin. Invest.* **49**, e13162 (2019).

12. E. J. Calabrese, LNT and cancer risk assessment: Its flawed foundations part 1: Radiation and leukemia: Where LNT began. *Environ. Res.* **197**, 111025 (2021).

13. IARC Working Group on the Evaluation of Carcinogenic Risks to Humans, *Non-Ionizing Radiation, Part 1: Static and Extremely Low-Frequency (ELF) Electric and Magnetic Fields* (International Agency for Research on Cancer, 2002), vol. 80.

14. "Cancer prevention during early life" (CDC, 2021); www.cdc.gov/cancer/dcpc/prevention/childhood.htm.

15. "Oral health care drug products for over-the-counter human use; antigingivitis/antiplaque drug products; establishment of a monograph" (Federal Register, 2003); www.federalregister.gov/documents/2003/05/29/03-12783/oral-health-care-drug-products-for-over-the-counter-human-use-antigingivitisantiplaque-drug-products.

16. J. L. Martino, S. H. Vermund, Vaginal douching: Evidence for risks or benefits to women's health. *Epidemiol. Rev.* **24**, 109–124 (2002).

17. L. B. Alexandrov, J. Kim, N. J. Haradhvala, M. N. Huang, A. W. Tian Ng, Y. Wu, A. Boot, K. R. Covington, D. A. Gordenin, E. N. Bergstrom, S. M. A. Islam, N. Lopez-Bigas, L. J. Klimczak, J. R. McPherson, S. Morganella, R. Sabarinathan, D. A. Wheeler, V. Mustonen, G. Getz, S. G. Rozen, M.

R. Stratton; PCAWG Consortium, The repertoire of mutational signatures in human cancer. *Nature* **578**, 94–101 (2020).

18. M. T. Landi, N. C. Synnott, J. Rosenbaum, T. Zhang, B. Zhu, J. Shi, W. Zhao, M. Kebede, J. Sang, J. Choi, L. Mendoza, M. Pacheco, B. Hicks, N. E. Caporaso, M. Abubakar, D. A. Gordenin, D. C. Wedge, L. B. Alexandrov, N. Rothman, Q. Lan, M. Garcia-Closas, S. J. Chanock, Tracing lung cancer risk factors through mutational signatures in never-smokers. *Am. J. Epidemiol.* **190**, 962–976 (2020).

19. T. L. Lash, M. P. Fox, A. K. Fink, *Applying Quantitative Bias Analysis to Epidemiologic Data* (Springer, 2009), vol. 533.

20. J. K. Litton, H. J. Burstein, N. C. Turner, Molecular testing in breast cancer. *Am. Soc. Clin. Oncol. Educ. Book* **39**, e1–e7 (2019).

21. X. R. Yang, J. Chang-Claude, E. L. Goode, F. J. Couch, H. Nevanlinna, R. L. Milne, M. Gaudet, M. K. Schmidt, A. Broeks, A. Cox, P. A. Fasching, R. Hein, A. B. Spurdle, F. Blows, K. Driver, D. Flesch-Janys, J. Heinz, P. Sinn, A. Vrieling, T. Heikkinen, K. Aittomäki, P. Heikkilä, C. Blomqvist, J. Lissowska, B. Peplonska, S. Chanock, J. Figueroa, L. Brinton, P. Hall, K. Czene, K. Humphreys, H. Darabi, J. Liu, L. J. Van ‘t Veer, F. E. van Leeuwen, I. L. Andrulis, G. Glendon, J. A. Knight, A. M. Mulligan, F. P. O’Malley, N. Weerasooriya, E. M. John, M. W. Beckmann, A. Hartmann, S. B. Weihbrecht, D. L. Wachter, S. M. Jud, C. R. Loehberg, L. Baglietto, D. R. English, G. G. Giles, C. A. McLean, G. Severi, D. Lambrechts, T. Vandorpe, C. Weltens, R. Paridaens, A. Smeets, P. Neven, H. Wildiers, X. Wang, J. E. Olson, V. Cafourek, Z. Fredericksen, M. Kosel, C. Vachon, H. E. Cramp, D. Connley, S. S. Cross, S. P. Balasubramanian, M. W. R. Reed, T. Dörk, M. Bremer, A. Meyer, J. H. Karstens, A. Ay, T.-W. Park-Simon, P. Hillemanns, J. I. Arias Pérez, P. M. Rodríguez, P. Zamora, J. Benítez, Y.-D. Ko, H.-P. Fischer, U. Hamann, B. Pesch, T. Brüning, C. Justenhoven, H. Brauch, D. M. Eccles, W. J. Tapper, S. M. Gerty, E. J. Sawyer, I. P. Tomlinson, A. Jones, M. Kerin, N. Miller, N. McInerney, H. Anton-Culver, A. Ziogas, C.-Y. Shen, C.-N. Hsiung, P.-E. Wu, S.-L. Yang, J.-C. Yu, S.-T. Chen, G.-C. Hsu, C. A. Haiman, B. E. Henderson, L. Le Marchand, L. N. Kolonel, A. Lindblom, S. Margolin, A. Jakubowska, J. Lubiński, T. Huzarski, T. Byrski, B. Górski, J. Gronwald, M. J. Hooning, A. Hollestelle, A. M. W. van den Ouweland, A. Jager, M. Kriege, M. M. A. Tilanus-Linthorst, M. Collée, S. Wang-Gohrke, K. Pylkäs, A. Jukkola-Vuorinen, K. Mononen, M. Grip, P. Hirvikoski, R. Winqvist, A. Mannermaa, V.-M. Kosma, J. Kauppinen, V. Kataja, P. Auvinen, Y. Soini, R. Sironen, S. E. Bojesen, D. Dynnes Ørsted, D. Kaur-Knudsen, H. Flyger, B. G. Nordestgaard, H. Holland, G. Chenevix-Trench, S. Manoukian, M. Barile, P. Radice, S. E. Hankinson, D. J. Hunter, R. Tamimi, S. Sangrajrang, P. Brennan, J. McKay, F. Odefrey, V. Gaborieau, P. Devilee, P. E. A. Huijts, RAEM. Tollenaar, C. Seynaeve, G. S. Dite, C. Apicella, J. L. Hopper, F. Hammet, H. Tsimiklis, L. D. Smith, M. C. Southey, M. K. Humphreys, D. Easton, P. Pharoah, M. E. Sherman, M. Garcia-Closas, Associations of breast cancer risk factors with tumor subtypes: A pooled analysis from the breast cancer association consortium studies. *J. Natl. Cancer Inst.* **103**, 250–263 (2011).

22. N. Krieger, G. Davey Smith, The tale wagged by the DAG: Broadening the scope of causal inference and explanation for epidemiology. *Int. J. Epidemiol.* **45**, 1787–1808 (2016).

23. S. Greenland, J. Pearl, J. M. Robins, Causal diagrams for epidemiologic research. *Epidemiology* **10**, 37–48 (1999).

24. K. M. Keyes, G. Davey Smith, E. Susser, Commentary: Smoking in pregnancy and offspring health: Early insights into family-based and ‘negative control’ studies? *Int. J. Epidemiol.* **43**, 1381–1388 (2014).

25. M. Lipsitch, E. Tchetgen Tchetgen, T. Cohen, Negative controls: A tool for detecting confounding and bias in observational studies. *Epidemiology* **21**, 383–388 (2010).

26. G. D. Smith, Negative control exposures in epidemiologic studies. *Epidemiology* **23**, 351–352 (2012).

27. W. D. Flanders, M. Klein, L. A. Darrow, M. J. Strickland, S. E. Sarnat, J. A. Sarnat, L. A. Waller, A. Winquist, P. E. Tolbert, A method for detection of residual confounding in time-series and other observational studies. *Epidemiology* **22**, 59–67 (2011).

28. V. Didelez, N. Sheehan, Mendelian randomization as an instrumental variable approach to causal inference. *Stat. Methods Med. Res.* **16**, 309–330 (2007).

29. G. D. Smith, S. Ebrahim, Mendelian randomization: Prospects, potentials, and limitations. *Int. J. Epidemiol.* **33**, 30–42 (2004).

30. B. D. Meyer, Natural and quasi-experiments in economics. *J. Bus. Econ. Stat.* **13**, 151–161 (1995).

31. M. Lechner, “The estimation of causal effects by difference-in-difference methods,” University of St. Gallen Department of Economics working paper series 2010 (2010–28), Department of Economics, University of St. Gallen, October 2011; https://ideas.repec.org/p/usg/dp2010/2010-28.html.

32. A. Abadie, A. Diamond, J. Hainmueller, Synthetic control methods for comparative case studies: Estimating the effect of California’s tobacco control program. *J. Am. Stat. Assoc.* **105**, 493–505 (2010).

33. C. E. Oldenburg, E. Moscoe, T. Bärnighausen, Regression discontinuity for causal effect estimation in epidemiology. *Curr. Epidemiol. Rep.* **3**, 233–241 (2016).

34. T. D. Cook, D. T. Campbell, W. Shadish, *Experimental and Quasi-Experimental Designs for Generalized Causal Inference* (Houghton Mifflin, 2002).

35. B. L. Pierce, P. Kraft, C. Zhang, Mendelian randomization studies of cancer risk: A literature review. *Curr. Epidemiol. Rep.* **5**, 184–196 (2018).

36. S. J. Lewis, G. D. Smith, Alcohol, ALDH2, and esophageal cancer: A meta-analysis which illustrates the potentials and limitations of a Mendelian randomization approach. *Cancer Epidemiol. Biomarkers Prev.* **14**, 1967–1971 (2005).

37. S. Boccia, M. Hashibe, P. Gallì, E. De Feo, T. Asakage, T. Hashimoto, A. Hiraki, T. Katoh, T. Nomura, A. Yokoyama, C. M. van Duijn, G. Ricciardi, P. Boffetta, Aldehyde dehydrogenase 2 and

head and neck cancer: A meta-analysis implementing a Mendelian randomization approach. *Cancer Epidemiol. Biomarkers Prev.* **18**, 248–254 (2009).

38. J.-S. Ong, E. M. Derks, M. Eriksson, J. An, L.-D. Hwang, D. F. Easton, P. P. Pharoah, A. Berchuck, L. E. Kelemen, K. Matsuo, G. Chenevix-Trench, P. Hall, S. E. Bojesen, P. M. Webb, S. MacGregor, Evaluating the role of alcohol consumption in breast and ovarian cancer susceptibility using population-based cohort studies and two-sample Mendelian randomization analyses. *Int. J. Cancer* **148**, 1338–1350 (2021).

39. J. Zhu, X. Jiang, Z. Niu, Alcohol consumption and risk of breast and ovarian cancer: A Mendelian randomization study. *Cancer Genet.* **245**, 35–41 (2020).

40. S. C. Larsson, P. Carter, S. Kar, M. Vithayathil, A. M. Mason, K. Michaëlsson, S. Burgess, Smoking, alcohol consumption, and cancer: A Mendelian randomisation study in UK Biobank and international genetic consortia participants. *PLOS Med.* **17**, e1003178 (2020).

41. S. A. Swanson, H. Tiemeier, M. A. Ikram, M. A. Hernán, Nature as a trialist?: Deconstructing the analogy between Mendelian randomization and randomized trials *Epidemiology* **28**, 653–659 (2017).

42. M. M. Glymour, E. J. Tchetgen Tchetgen, J. M. Robins, Credible Mendelian randomization studies: Approaches for evaluating the instrumental variable assumptions. *Am. J. Epidemiol.* **175**, 332–339 (2012).

43. W. Gunn, Mendeley: Enabling and understanding scientific collaboration. *Inf. Serv. Use* **34**, 99–102 (2014).

44. J. Koshiol, L. Lindsay, J. M. Pimenta, C. Poole, D. Jenkins, J. S. Smith, Persistent human papillomavirus infection and cervical neoplasia: A systematic review and meta-analysis. *Am. J. Epidemiol.* **168**, 123–137 (2008).

45. R. Saulle, L. Semyonov, A. Mannocci, A. Careri, F. Saburri, L. Ottolenghi, F. Guerra, G. La Torre, Human papillomavirus and cancerous diseases of the head and neck: A systematic review and meta-analysis. *Oral Dis.* **21**, 417–431 (2015).

46. IARC Working Group on the Evaluation of Carcinogenic Risks to Humans, *Human Papillomaviruses* (International Agency for Research on Cancer, 2007), vol. 90.

47. "Cancers associated with human papillomavirus (HPV)" (CDC, 2021); www.cdc.gov/cancer/hpv/basic_info/cancers.htm.

48. E. Ron, J. H. Lubin, R. E. Shore, K. Mabuchi, B. Modan, L. M. Pottern, A. B. Schneider, M. A. Tucker, J. D. Boice Jr., Thyroid cancer after exposure to external radiation: A pooled analysis of seven studies. *Radiat. Res.* **141**, 259–277 (1995).

49. E. Cardis, M. Vrijheid, M. Blettner, E. Gilbert, M. Hakama, C. Hill, G. Howe, J. Kaldor, C. R. Muirhead, M. Schubauer-Berigan, T. Yoshimura, F. Bermann, G. Cowper, J. Fix, C. Hacker, B. Heinmiller, M. Marshall, I. Thierry-Chef, D. Utterback, Y.-O. Ahn, E. Amoros, P. Ashmore, A. Auvinen, J.-M. Bae, J. Bernar, A. Biau, E. Combalot, P. Deboodt, A. Diez Sacristan, M. Eklöf, H. Engels, G. Engholm, G. Gulis, R. R. Habib, K. Holan, H. Hyvonen, A. Kerekes, J. Kurtinaitis, H.

Malker, M. Martuzzi, A. Mastauskas, A. Monnet, M. Moser, M. S. Pearce, D. B. Richardson, F. Rodriguez-Artalejo, A. Rogel, H. Tardy, M. Telle-Lamberton, I. Turai, M. Usel, K. Veress, The 15-country collaborative study of cancer risk among radiation workers in the nuclear industry: Estimates of radiation-related cancer risks. *Radiat. Res.* **167**, 396–416 (2007).

50. R. D. Daniels, M. K. Schubauer-Berigan, A meta-analysis of leukaemia risk from protracted exposure to low-dose gamma radiation. *Occup. Environ. Med.* **68**, 457–464 (2011).

51. IARC Working Group on the Evaluation of Carcinogenic Risks to Humans, *Ionizing Radiation, Part 1: X-and Gamma (γ)-Radiation, and Neutrons* (International Agency for Research on Cancer, 2000), vol. 75.

52. L. Y. Cho, J. J. Yang, K.-P. Ko, B. Park, A. Shin, M. K. Lim, J.-K. Oh, S. Park, Y. J. Kim, H.-R. Shin, K.-Y. Yoo, S. K. Park, Coinfection of hepatitis B and C viruses and risk of hepatocellular carcinoma: Systematic review and meta-analysis. *Int. J. Cancer* **128**, 176–184 (2011).

53. X. Chen, F. Wu, Y. Liu, J. Lou, B. Zhu, L. Zou, W. Chen, J. Gong, Y. Wang, R. Zhong, The contribution of serum hepatitis B virus load in the carcinogenesis and prognosis of hepatocellular carcinoma: Evidence from two meta-analyses. *Oncotarget* **7**, 49299–49309 (2016).

54. M. Li, Y. Gan, C. Fan, H. Yuan, X. Zhang, Y. Shen, Q. Wang, Z. Meng, D. Xu, H. Tu, Hepatitis B virus and risk of non-Hodgkin lymphoma: An updated meta-analysis of 58 studies. *J. Viral Hepat.* **25**, 894–903 (2018).

55. A. B. Ryerson, C. R. Eheman, S. F. Altekruse, J. W. Ward, A. Jemal, R. L. Sherman, S. J. Henley, D. Holtzman, A. Lake, A.-M. Noone, R. N. Anderson, J. Ma, K. N. Ly, K. A. Cronin, L. Penberthy, B. A. Kohler, Annual Report to the Nation on the Status of Cancer, 1975-2012, featuring the increasing incidence of liver cancer. *Cancer* **122**, 1312–1337 (2016).

56. IARC Working Group on the Evaluation of Carcinogenic Risks to Humans, *Biological Agents* (International Agency for Research on Cancer, 2012), vol. 100B; www.ncbi.nlm.nih.gov/books/NBK304348/.

57. S. Gandini, E. Botteri, S. Iodice, M. Boniol, A. B. Lowenfels, P. Maisonneuve, P. Boyle, Tobacco smoking and cancer: A meta-analysis. *Int. J. Cancer* **122**, 155–164 (2008).

58. P. N. Lee, B. A. Forey, K. J. Coombs, Systematic review with meta-analysis of the epidemiological evidence in the 1900s relating smoking to lung cancer. *BMC Cancer* **12**, 385 (2012).

59. J. M. Ordóñez-Mena, B. Schöttker, U. Mons, M. Jenab, H. Freisling, B. Bueno-de-Mesquita, M. G. O'Doherty, A. Scott, F. Kee, B. H. Stricker, A. Hofman, C. E. de Keyser, R. Ruiter, S. Söderberg, P. Jousilahti, K. Kuulasmaa, N. D. Freedman, T. Wilsgaard, L. C. de Groot, E. Kampman, N. Håkansson, N. Orsini, A. Wolk, L. M. Nilsson, A. Tjønneland, A. Pająk, S. Malyutina, R. Kubínová, A. Tamosiunas, M. Bobak, M. Katsoulis, P. Orfanos, P. Boffetta, A. Trichopoulou, H. Brenner, Consortium on Health and Ageing: Network of Cohorts in Europe and the United States (CHANCES), Quantification of the smoking-associated cancer risk with rate advancement periods: Meta-analysis of individual participant data from cohorts of the CHANCES consortium. *BMC Med.* **14**, 62 (2016).

60. IARC Working Group on the Evaluation of Carcinogenic Risks to Humans, *Personal Habits and Indoor Combustions* (International Agency for Research on Cancer, 2012), vol. 100E; www.ncbi.nlm.nih.gov/books/NBK304391/.

61. S. Gandini, F. Sera, M. S. Cattaruzza, P. Pasquini, O. Picconi, P. Boyle, C. F. Melchi, Meta-analysis of risk factors for cutaneous melanoma: II. Sun exposure. *Eur. J. Cancer* **41**, 45–60 (2005).

62. L. K. Dennis, M. J. Vanbeek, L. E. Beane Freeman, B. J. Smith, D. V. Dawson, J. A. Coughlin, Sunburns and risk of cutaneous melanoma: Does age matter? A comprehensive meta-analysis. *Ann. Epidemiol.* **18**, 614–627 (2008).

63. C. M. Olsen, M. S. Zens, A. C. Green, T. A. Stukel, C. D. J. Holman, T. Mack, J. M. Elwood, E. A. Holly, C. Sacerdote, R. Gallagher, A. J. Swerdlow, B. K. Armstrong, S. Rosso, C. Kirkpatrick, R. Zanetti, J. N. Bishop, V. Bataille, Y.-M. Chang, R. Mackie, A. Østerlind, M. Berwick, M. R. Karagas, D. C. Whiteman, Biologic markers of sun exposure and melanoma risk in women: Pooled case-control analysis. *Int. J. Cancer* **129**, 713–723 (2011).

64. "Risk factors: Sunlight" (National Cancer Institute, 2015); www.cancer.gov/about-cancer/causes-prevention/risk/sunlight.

65. IARC Working Group on the Evaluation of Carcinogenic Risks to Humans, *Radiation* (International Agency for Research on Cancer, 2012), vol. 100D; https://publications.iarc.fr/Book-And-Report-Series/Iarc-Monographs-On-The-Identification-Of-Carcinogenic-Hazards-To-Humans/Radiation-2012.

66. V. Bagnardi, M. Rota, E. Botteri, I. Tramacere, F. Islami, V. Fedirko, L. Scotti, M. Jenab, F. Turati, E. Pasquali, C. Pelucchi, C. Galeone, R. Bellocco, E. Negri, G. Corrao, P. Boffetta, C. La Vecchia, Alcohol consumption and site-specific cancer risk: A comprehensive dose-response meta-analysis. *Br. J. Cancer* **112**, 580–593 (2015).

67. H. Jayasekara, R. J. MacInnis, R. Room, D. R. English, Long-term alcohol consumption and breast, upper aero-digestive tract and colorectal cancer risk: A systematic review and meta-analysis. *Alcohol Alcohol.* **51**, 315–330 (2016).

68. Y.-J. Choi, S.-K. Myung, J.-H. Lee, Light alcohol drinking and risk of cancer: A meta-analysis of cohort studies. *Cancer Res. Treat.* **50**, 474–487 (2018).

69. "Alcohol and cancer" (CDC, 2022); www.cdc.gov/cancer/alcohol/index.htm.

70. M. C. Camargo, L. T. Stayner, K. Straif, M. Reina, U. Al-Alem, P. A. Demers, P. J. Landrigan, Occupational exposure to asbestos and ovarian cancer: A meta-analysis. *Environ. Health Perspect.* **119**, 1211–1217 (2011).

71. V. Lenters, R. Vermeulen, S. Dogger, L. Stayner, L. Portengen, A. Burdorf, D. Heederik, A Meta-analysis of asbestos and lung cancer: Is better quality exposure assessment associated with steeper slopes of the exposure–response relationships? *Environ. Health Perspect.* **119**, 1547–1555 (2011).

72. Y. Ngamwong, W. Tangamornsuksan, O. Lohitnavy, N. Chaiyakunapruk, C. N. Scholfield, B. Reisfeld, M. Lohitnavy, Additive synergism between asbestos and smoking in lung cancer risk: A systematic review and meta-analysis. *PLOS ONE* **10**, e0135798 (2015).

73. IARC Working Group on the Evaluation of Carcinogenic Risks to Humans, *Arsenic, Metals, Fibres and Dusts* (International Agency for Research on Cancer, 2012), vol. 100C; www.ncbi.nlm.nih.gov/books/NBK304375/.

74. U.S. Environmental Protection Agency, Asbestos CASRN 1332-21-4 | DTXSID4023888; https://cfpub.epa.gov/ncea/iris2/chemicallanding.cfm?substance_nmbr=371.

75. B. Armstrong, E. Hutchinson, J. Unwin, T. Fletcher, Lung cancer risk after exposure to polycyclic aromatic hydrocarbons: A review and meta-analysis. *Environ. Health Perspect.* **112**, 970–978 (2004).

76. A. Singh, R. Kamal, I. Ahamed, M. Wagh, V. Bihari, B. Sathian, C. N. Kesavachandran, PAH exposure-associated lung cancer: An updated meta-analysis. *Occup. Med.* **68**, 255–261 (2018).

77. U.S. Environmental Protection Agency, Coke oven emissions CASRN NA | DTXSID7023984; https://cfpub.epa.gov/ncea/iris2/chemicalLanding.cfm?substance_nmbr=395.

78. IARC Working Group on the Evaluation of Carcinogenic Risks to Humans, *Chemical Agents and Related Occupations* (International Agency for Research on Cancer, 2012), vol. 100F; www.ncbi.nlm.nih.gov/books/NBK304416/.

79. H. Ma, L. Bernstein, M. C. Pike, G. Ursin, Reproductive factors and breast cancer risk according to joint estrogen and progesterone receptor status: A meta-analysis of epidemiological studies. *Breast Cancer Res.* **8**, R43 (2006).

80. G. K. Reeves, K. Pirie, J. Green, D. Bull, V. Beral, Reproductive factors and specific histological types of breast cancer: Prospective study and meta-analysis, *Br. J. Cancer*. **100**, 538–544 (2009).

81. M. Lambertini, L. Santoro, L. Del Mastro, B. Nguyen, L. Livraghi, D. Ugolini, F. A. Peccatori, H. A. Azim Jr., Reproductive behaviors and risk of developing breast cancer according to tumor subtype: A systematic review and meta-analysis of epidemiological studies. *Cancer Treat. Rev.* **49**, 65–76 (2016).

82. "Reproductive history and cancer risk (National Cancer Institute, 2016); www.cancer.gov/about-cancer/causes-prevention/risk/hormones/reproductive-history-fact-sheet.

83. *Diet, Nutrition, Physical Activity and Breast Cancer* (World Cancer Research Fund/American Institute for Cancer Research, 2017), 124p.

84. C. Hoyo, M. B. Cook, F. Kamangar, N. D. Freedman, D. C. Whiteman, L. Bernstein, L. M. Brown, H. A. Risch, W. Ye, L. Sharp, A. H. Wu, M. H. Ward, A. G. Casson, L. J. Murray, D. A. Corley, O. Nyrén, N. Pandeya, T. L. Vaughan, W.-H. Chow, M. D. Gammon, Body mass index in relation to oesophageal and oesophagogastric junction adenocarcinomas: A pooled analysis from the International BEACON Consortium. *Int. J. Epidemiol.* **41**, 1706–1718 (2012).

85. F. Turati, I. Tramacere, C. La Vecchia, E. Negri, A meta-analysis of body mass index and esophageal and gastric cardia adenocarcinoma. *Ann. Oncol.* **24**, 609–617 (2013).

86. *Diet, Nutrition, Physical Activity and Oesophageal Cancer* (World Cancer Research Fund/American Institute for Cancer Research, 2016), 62p.

87. IARC Working Group on the Evaluation of Carcinogenic Risks to Humans, *Absence of Excess Body Fatness*, vol. 16 of *IARC Handbooks of Cancer Prevention* (2018); https://publications.iarc.fr/Book-And-Report-Series/Iarc-Handbooks-Of-Cancer-Prevention/Absence-Of-Excess-Body-Fatness-2018.

88. S. M. Lynch, A. Vrieling, J. H. Lubin, P. Kraft, J. B. Mendelsohn, P. Hartge, F. Canzian, E. Steplowski, A. A. Arslan, M. Gross, K. Helzlsouer, E. J. Jacobs, A. LaCroix, G. Petersen, W. Zheng, D. Albanes, L. Amundadottir, S. A. Bingham, P. Boffetta, M.-C. Boutron-Ruault, S. J. Chanock, S. Clipp, R. N. Hoover, K. Jacobs, K. C. Johnson, C. Kooperberg, J. Luo, C. Messina, D. Palli, A. V. Patel, E. Riboli, X.-O. Shu, L. Rodriguez Suarez, G. Thomas, A. Tjønneland, G. S. Tobias, E. Tong, D. Trichopoulos, J. Virtamo, W. Ye, K. Yu, A. Zeleniuch-Jacquette, H. B. Bueno-de-Mesquita, R. Z. Stolzenberg-Solomon, Cigarette smoking and pancreatic cancer: A pooled analysis from the pancreatic cancer cohort consortium. *Am. J. Epidemiol.* **170**, 403–413 (2009).

89. G. La Torre, C. de Waure, M. L. Specchia, N. Nicolotti, S. Capizzi, A. Bilotta, G. Clemente, W. Ricciardi, Does quality of observational studies affect the results of a meta-analysis?: The case of cigarette smoking and pancreatic cancer. *Pancreas* **38**, 241–247 (2009).

90. L. Zou, R. Zhong, N. Shen, W. Chen, B. Zhu, J. Ke, X. Lu, T. Zhang, J. Lou, Z. Wang, L. Liu, L. Qi, X. Miao, Non-linear dose-response relationship between cigarette smoking and pancreatic cancer risk: Evidence from a meta-analysis of 42 observational studies. *Eur. J. Cancer* **50**, 193–203 (2014).

91. L. Leng, X. Chen, C.-P. Li, X.-Y. Luo, N.-J. Tang, 2,3,7,8-Tetrachlorodibezo-p-dioxin exposure and prostate cancer: A meta-analysis of cohort studies. *Public Health* **128**, 207–213 (2014).

92. J. Xu, Y. Ye, F. Huang, H. Chen, H. Wu, J. Huang, J. Hu, D. Xia, Y. Wu, Association between dioxin and cancer incidence and mortality: A meta-analysis. *Sci. Rep.* **6**, 38012 (2016).

93. “Lifestyle-related breast cancer risk factors” (American Cancer Society, 2021); www.cancer.org/cancer/breast-cancer/risk-and-prevention/lifestyle-related-breast-cancer-risk-factors.html.

94. Z.-L. Zhang, J. Sun, J.-Y. Dong, H.-L. Tian, L. Xue, L.-Q. Qin, J. Tong, Residential radon and lung cancer risk: An updated meta- analysis of case-control studies. *Asian Pac. J. Cancer Prev.* **13**, 2459–2465 (2012).

95. P. Duan, C. Quan, C. Hu, J. Zhang, F. Xie, X. Hu, Z. Yu, B. Gao, Z. Liu, H. Zheng, C. Liu, C. Wang, T. Yu, S. Qi, W. Fu, A. Kourouma, K. Yang, Nonlinear dose-response relationship between radon exposure and the risk of lung cancer: Evidence from a meta-analysis of published observational studies. *Eur. J. Cancer Prev.* **24**, 267–277 (2015).

96. C. Garzillo, M. Pugliese, F. Loffredo, M. Quarto, Indoor radon exposure and lung cancer risk: A meta-analysis of case-control studies. *Transl. Cancer Res.* **6**, 1–10 (2017).

97. U.S. Environmental Protection Agency, “A citizen’s guide to Radon: The guide to protecting yourself and your family from Radon” (2012); www.epa.gov/sites/production/files/2016-02/documents/2012_a_citizens_guide_to_radon.pdf.

98. T. Norat, A. Lukanova, P. Ferrari, E. Riboli, Meat consumption and colorectal cancer risk: Dose-response meta-analysis of epidemiological studies. *Int. J. Cancer* **98**, 241–256 (2002).

99. N. M. Pham, T. Mizoue, K. Tanaka, I. Tsuji, A. Tamakoshi, K. Matsuo, K. Wakai, C. Nagata, M. Inoue, S. Tsugane, S. Sasazuki; Research Group for the Development and Evaluation of Cancer Prevention Strategies in Japan, Meat consumption and colorectal cancer risk: An evaluation based on a systematic review of epidemiologic evidence among the Japanese population. *Jpn. J. Clin. Oncol.* **44**, 641–650 (2014).

100. A. R. Vieira, L. Abar, D. S. M. Chan, S. Vingeliene, E. Polemiti, C. Stevens, D. Greenwood, T. Norat, Foods and beverages and colorectal cancer risk: A systematic review and meta-analysis of cohort studies, an update of the evidence of the WCRF-AICR Continuous Update Project. *Ann. Oncol.* **28**, 1788–1802 (2017).

101. IARC Working Group on the Evaluation of Carcinogenic Risks to Humans, *Red Meat and Processed Meat* (International Agency for Research on Cancer, 2018), vol. 114; www.ncbi.nlm.nih.gov/books/NBK507971/.

102. *Diet, Nutrition, Physical Activity and Colorectal Cancer* (Continuous Update Project Expert Report 2018, World Cancer Research Fund/American Institute for Cancer Research, 2018); www.wcrf.org/wp-content/uploads/2021/02/Colorectal-cancer-report.pdf.

103. S. K. Park, D. Kang, K. A. McGlynn, M. Garcia-Closas, Y. Kim, K. Y. Yoo, L. A. Brinton, Intrauterine environments and breast cancer risk: Meta-analysis and systematic review. *Breast Cancer Res.* **10**, R8 (2008).

104. X. Xu, A. B. Dailey, M. Peoples-Sheps, E. O. Talbott, N. Li, J. Roth, Birth weight as a risk factor for breast cancer: A meta-analysis of 18 epidemiological studies. *J. Womens Health* **18**, 1169–1178 (2009).

105. “Birthweight and breast cancer risk” (Susan G Komen, 2021); www.komen.org/breast-cancer/facts-statistics/research-studies/topics/birthweight-and-breast-cancer-risk/.

106. J. M. Gierisch, R. R. Coeytaux, R. P. Urrutia, L. J. Havrilesky, P. G. Moorman, W. J. Lowery, M. Dinan, A. J. McBroom, V. Hasselblad, G. D. Sanders, E. R. Myers, Oral contraceptive use and risk of breast, cervical, colorectal, and endometrial cancers: A systematic review. *Cancer Epidemiol. Biomarkers Prev.* **22**, 1931–1943 (2013).

107. L. Li, Y. Zhong, H. Zhang, H. Yu, Y. Huang, Z. Li, G. Chen, X. Hua, Association between oral contraceptive use as a risk factor and triple-negative breast cancer: A systematic review and meta-analysis. *Mol. Clin. Oncol.* **7**, 76–80 (2017).

108. IARC Working Group on the Evaluation of Carcinogenic Risks to Humans, *Pharmaceuticals* (International Agency for Research on Cancer, 2012), vol. 100A; www.ncbi.nlm.nih.gov/books/NBK304334/.

109. "Oral contraceptives (birth control pills) and cancer risk" (National Cancer Institute, 2018); www.cancer.gov/about-cancer/causes-prevention/risk/hormones/oral-contraceptives-fact-sheet.

110. International Agency for Research on Cancer Working Group on artificial ultraviolet (UV) light and skin cancer, The association of use of sunbeds with cutaneous malignant melanoma and other skin cancers: A systematic review. *Int. J. Cancer* **120**, 1116–1122 (2007).

111. M. Boniol, P. Autier, P. Boyle, S. Gandini, Cutaneous melanoma attributable to sunbed use: Systematic review and meta-analysis. *BMJ* **345**, e4757 (2012).

112. S. Colantonio, M. B. Bracken, J. Beecker, The association of indoor tanning and melanoma in adults: Systematic review and meta-analysis. *J. Am. Acad. Dermatol.* **70**, 847–857.e1–18 (2014).

113. IARC Working Group on the Evaluation of Carcinogenic Risks to Humans, *Radiation* (International Agency for Research on Cancer, 2012), vol. 100D; www.ncbi.nlm.nih.gov/books/NBK304366/.

114. D. S. M. Chan, R. Lau, D. Aune, R. Vieira, D. C. Greenwood, E. Kampman, T. Norat, Red and processed meat and colorectal cancer incidence: Meta-analysis of prospective studies. *PlOS ONE* **6**, e20456 (2011).

115. M. Chiavarini, G. Bertarelli, L. Minelli, R. Fabiani, Dietary intake of meat cooking-related mutagens (HCAs) and risk of colorectal adenoma and cancer: A systematic review and meta-analysis. *Nutrients* **9**, 514 (2017).

116. Z. Zhao, Q. Feng, Z. Yin, J. Shuang, B. Bai, P. Yu, M. Guo, Q. Zhao, Red and processed meat consumption and colorectal cancer risk: A systematic review and meta-analysis. *Oncotarget* **8**, 83306–83314 (2017).

117. M. L. Kwan, P. A. Buffler, B. Abrams, V. A. Kiley, Breastfeeding and the risk of childhood leukemia: A meta-analysis. *Public Health Rep.* **119**, 521–535 (2004).

118. R. M. Martin, D. Gunnell, C. G. Owen, G. D. Smith, Breast-feeding and childhood cancer: A systematic review with metaanalysis. *Int. J. Cancer* **117**, 1020–1031 (2005).

119. E. L. Amitay, L. Keinan-Boker, Breastfeeding and childhood leukemia incidence: A meta-analysis and systematic review. *JAMA Pediatr.* **169**, e151025 (2015).

120. "Cancer in children and adolescents" (National Cancer Institute, 2021); www.cancer.gov/types/childhood-cancers/child-adolescent-cancers-fact-sheet.

121. U.S. Department of Health and Human Services, "Making the decision to breastfeed" (Office on Women's Health, 2021); www.womenshealth.gov/breastfeeding/making-decision-breastfeed.

122. S. Gandini, E. Negri, P. Boffetta, C. La Vecchia, P. Boyle, Mouthwash and oral cancer risk quantitative meta-analysis of epidemiologic studies. *Ann. Agric. Environ. Med.* **19**, 173–180 (2012).

123. P. Boffetta, R. B. Hayes, S. Sartori, Y.-C. A. Lee, J. Muscat, A. Olshan, D. M. Winn, X. Castellsagué, Z.-F. Zhang, H. Morgenstern, C. Chen, S. M. Schwartz, T. L. Vaughan, V. Wunsch-Filho, M. Purdue, S. Koifman, M. P. Curado, M. Vilensky, M. Gillison, L. Fernandez, A. Menezes, A. W. Daudt, S. Schantz, G. Yu, G. D'Souza, R. I. Haddad, C. La Vecchia, M. Hashibe, Mouthwash use and cancer of the head and neck: A pooled analysis from the International Head and Neck Cancer Epidemiology Consortium. *Eur. J. Cancer Prev.* **25**, 344–348 (2016).

124. "Risk factors for oral cavity and oropharyngeal cancers" (American Cancer Society, 2021); www.cancer.org/cancer/oral-cavity-and-oropharyngeal-cancer/causes-risks-prevention/risk-factors.html.

125. D. Wartenberg, Residential EMF exposure and childhood leukemia: Meta-analysis and population attributable risk. *Bioelectromagnetics* (Suppl. 5), S86–S104 (2001).

126. L. Kheifets, A. Ahlbom, C. M. Crespi, G. Draper, J. Hagihara, R. M. Lowenthal, G. Mezei, S. Oksuzyan, J. Schüz, J. Swanson, A. Tittarelli, M. Vinceti, V. Wunsch Filho, Pooled analysis of recent studies on magnetic fields and childhood leukaemia. *Br. J. Cancer* **103**, 1128–1135 (2010).

127. L. Kheifets, A. Ahlbom, C. M. Crespi, M. Feychting, C. Johansen, J. Monroe, M. F. G. Murphy, S. Oksuzyan, S. Preston-Martin, E. Roman, T. Saito, D. Savitz, J. Schüz, J. Simpson, J. Swanson, T. Tynes, P. Verkasalo, G. Mezei, A pooled analysis of extremely low-frequency magnetic fields and childhood brain tumors. *Am. J. Epidemiol.* **172**, 752–761 (2010).

128. *Extremely Low Frequency Fields* (Environmental Health Criteria Monograph 238, World Health Organization, 2007); www.who.int/publications-detail-redirect/9789241572385.

129. "Electromagnetic fields and cancer" (National Cancer Institute, 2019); www.cancer.gov/about-cancer/causes-prevention/risk/radiation/electromagnetic-fields-fact-sheet.

130. V. L. Boothe, T. K. Boehmer, A. M. Wendel, F. Y. Yip, Residential traffic exposure and childhood leukemia: A systematic review and meta-analysis. *Am. J. Prev. Med.* **46**, 413–422 (2014).

131. F. M. Carlos-Wallace, L. Zhang, M. T. Smith, G. Rader, C. Steinmaus, Parental, in utero, and early-life exposure to benzene and the risk of childhood leukemia: A meta-analysis. *Am. J. Epidemiol.* **183**, 1–14 (2016).

132. T. Filippini, E. E. Hatch, K. J. Rothman, J. E. Heck, A. S. Park, A. Crippa, N. Orsini, M. Vinceti, association between outdoor air pollution and childhood leukemia: A systematic review and dose-response meta-analysis. *Environ. Health Perspect.* **127**, 46002 (2019).

133. IARC Working Group on the Evaluation of Carcinogenic Risks to Humans, *Benzene* (International Agency for Research on Cancer, 2018), vol. 120; www.ncbi.nlm.nih.gov/books/NBK550157/.

134. G. R. Howe, K. J. Aronson, E. Benito, R. Castelleto, J. Cornée, S. Duffy, R. P. Gallagher, J. M. Iscovich, J. Deng-ao, R. Kaaks, G. A. Kune, S. Kune, H. P. Lee, M. Lee, A. B. Miller, R. K. Peters, J. D. Potter, E. Riboli, M. L. Slattery, D. Trichopoulos, A. Tuyns, A. Tzonou, L. F. Watson, A. S. Whittemore, Z. Shu, The relationship between dietary fat intake and risk of colorectal cancer:

Evidence from the combined analysis of 13 case-control studies. *Cancer Causes Control* **8**, 215–228 (1997).

135. L. Liu, W. Zhuang, R.-Q. Wang, R. Mukherjee, S.-M. Xiao, Z. Chen, X.-T. Wu, Y. Zhou, H.-Y. Zhang, Is dietary fat associated with the risk of colorectal cancer? A meta-analysis of 13 prospective cohort studies. *Eur. J. Nutr.* **50**, 173–184 (2011).

136. "The cancer-cholesterol connection" (American Institute for Cancer Research, 2015); www.aicr.org/news/the-cancer-cholesterol-connection/.

137. J. Zhang, A. G. Thomas, E. Leybovich, Vaginal douching and adverse health effects: A meta-analysis. *Am. J. Public Health* **87**, 1207–1211 (1997).

138. "Vaginal douching and adverse health outcomes" (American Public Health Association, 2007); www.apha.org/policies-and-advocacy/public-health-policy-statements/policy-database/2014/07/29/13/06/vaginal-douching-and-adverse-health-outcomes.

139. U.S. Department of Health and Human Services, "Douching" (Office on Women's Health, 2021); www.womenshealth.gov/a-z-topics/douching#14.

140. K. Heikkilä, S. T. Nyberg, T. Theorell, E. I. Fransson, L. Alfredsson, J. B. Bjorner, S. Bonenfant, M. Borritz, K. Bouillon, H. Burr, N. Dragano, G. A. Geuskens, M. Goldberg, M. Hamer, W. E. Hooftman, I. L. Houtman, M. Joensuu, A. Knutsson, M. Koskenvuo, A. Koskinen, A. Kouvonen, I. E. H. Madsen, L. L. Magnusson Hanson, M. G. Marmot, M. L. Nielsen, M. Nordin, T. Oksanen, J. Pentti, P. Salo, R. Rugulies, A. Steptoe, S. Suominen, J. Vahtera, M. Virtanen, A. Väänänen, P. Westerholm, H. Westerlund, M. Zins, J. E. Ferrie, A. Singh-Manoux, G. D. Batty, M. Kivimäki, IPD-Work Consortium, Work stress and risk of cancer: Meta-analysis of 5700 incident cancer events in 116,000 European men and women. *BMJ* **346**, f165 (2013).

141. T. Yang, Y. Qiao, S. Xiang, W. Li, Y. Gan, Y. Chen, Work stress and the risk of cancer: A meta-analysis of observational studies. *Int. J. Cancer* **144**, 2390–2400 (2019).

142. "Psychological stress and cancer" (National Cancer Institute, 2012); www.cancer.gov/about-cancer/coping/feelings/stress-fact-sheet.

143. S. Bérubé, J. Lemieux, L. Moore, E. Maunsell, J. Brisson, Smoking at time of diagnosis and breast cancer-specific survival: New findings and systematic review with meta-analysis. *Breast Cancer Res.* **16**, R42 (2014).

144. K. Wang, F. Li, X. Zhang, Z. Li, H. Li, Smoking increases risks of all-cause and breast cancer specific mortality in breast cancer individuals: A dose-response meta-analysis of prospective cohort studies involving 39725 breast cancer cases. *Oncotarget* **7**, 83134–83147 (2016).

145. W. Duan, S. Li, X. Meng, Y. Sun, C. Jia, Smoking and survival of breast cancer patients: A meta-analysis of cohort studies. *Breast* **33**, 117–124 (2017).

146. "Healthy lifestyle for breast cancer survivors" (Susan G Komen, 2021); www.komen.org/breast-cancer/survivorship/healthy-lifestyle/.

147. B. Takkouche, M. Etminan, A. Montes-Martínez, Personal use of hair dyes and risk of cancer: A meta-analysis. *JAMA* **293**, 2516–2525 (2005).

148. IARC Working Group on the Evaluation of Carcinogenic Risks to Humans, *Some Aromatic Amines, Organic Dyes, and Related Exposures* (International Agency for Research on Cancer, 2010), vol. 99; www.ncbi.nlm.nih.gov/books/NBK385419/.

149. "Hair dyes and cancer risk" (American Cancer Society, 2020); www.cancer.org/cancer/cancer-causes/hair-dyes.html.

150. C. M. Villanueva, F. Fernández, N. Malats, J. O. Grimalt, M. Kogevinas, Meta-analysis of studies on individual consumption of chlorinated drinking water and bladder cancer. *J. Epidemiol. Community Health* **57**, 166–173 (2003).

151. N. Costet, C. M. Villanueva, J. J. K. Jaakkola, M. Kogevinas, K. P. Cantor, W. D. King, C. F. Lynch, M. J. Nieuwenhuijsen, S. Cordier, Water disinfection by-products and bladder cancer: Is there a European specificity? A pooled and meta-analysis of European case-control studies *Occup. Environ. Med.* **68**, 379–385 (2011).

152. Y. Bai, H. Yuan, J. Li, Y. Tang, C. Pu, P. Han, Relationship between bladder cancer and total fluid intake: A meta-analysis of epidemiological evidence. *World J. Surg. Oncol.* **12**, 223 (2014).

153. U.S. Environmental Protection Agency, Chlorine CASRN 7782-50-5; https://cfpub.epa.gov/ncea/iris2/chemicalLanding.cfm?substance_nmbr=405.

154. IARC Working Group on the Evaluation of Carcinogenic Risks to Humans, *Some Chemicals Present in Industrial and Consumer Products, Food and Drinking-Water* (International Agency for Research on Cancer, 2013); www.ncbi.nlm.nih.gov/books/NBK373192/.

155. S. C. Larsson, N. Orsini, A. Wolk, Milk, milk products and lactose intake and ovarian cancer risk: A meta-analysis of epidemiological studies. *Int. J. Cancer* **118**, 431–441 (2006).

156. J. M. Genkinger, D. J. Hunter, D. Spiegelman, K. E. Anderson, A. Arslan, W. L. Beeson, J. E. Buring, G. E. Fraser, J. L. Freudenheim, R. A. Goldbohm, S. E. Hankinson, D. R. Jacobs, A. Koushik, J. V. Lacey, S. C. Larsson, M. Leitzmann, M. L. McCullough, A. B. Miller, C. Rodriguez, T. E. Rohan, L. J. Schouten, R. Shore, E. Smit, A. Wolk, S. M. Zhang, S. A. Smith-Warner, Dairy products and ovarian cancer: A pooled analysis of 12 cohort studies. *Cancer Epidemiol. Biomark. Prev.* **15**, 364–372 (2006).

157. J. Liu, W. Tang, L. Sang, X. Dai, D. Wei, Y. Luo, J. Zhang, Milk, yogurt, and lactose intake and ovarian cancer risk: A meta-analysis. *Nutr. Cancer* **67**, 68–72 (2015).

158. *Diet, Nutrition, Physical Activity and Ovarian Cancer* (Continuous Update Project Expert Report 2018, World Cancer Research Fund/American Institute for Cancer Research); www.wcrf.org/wp-content/uploads/2021/02/ovarian-cancer-report.pdf.

159. Y. Zhang, S. D. Sanjose, P. M. Bracci, L. M. Morton, R. Wang, P. Brennan, P. Hartge, P. Boffetta, N. Becker, M. Maynadie, L. Foretova, P. Cocco, A. Staines, T. Holford, E. A. Holly, A. Nieters, Y.

Benavente, L. Bernstein, S. H. Zahm, T. Zheng, Personal use of hair dye and the risk of certain subtypes of non-Hodgkin lymphoma. *Am. J. Epidemiol.* **167**, 1321–1331 (2008).

160. L. Qin, H.-Y. Deng, S.-J. Chen, W. Wei, A meta-analysis on the relationship between hair dye and the incidence of non-Hodgkin's lymphoma. *Med. Princ. Pract.* **28**, 222–230 (2019).

161. "Hair dyes and cancer risk" (National Cancer Institute, 2017); www.cancer.gov/about-cancer/causes-prevention/risk/myths/hair-dyes-fact-sheet.

162. G. Mezei, M. Gadallah, L. Kheifets, Residential magnetic field exposure and childhood brain cancer: A meta-analysis. *Epidemiology* **19**, 424–430 (2008).

163. L. Kheifets, J. Monroe, X. Vergara, G. Mezei, A. A. Afifi, Occupational electromagnetic fields and leukemia and brain cancer: An update to two meta-analyses. *J. Occup. Environ. Med.* **50**, 677–688 (2008).

164. K. M. Towle, M. E. Grespin, A. D. Monnot, Personal use of hair dyes and risk of leukemia: A systematic literature review and meta-analysis. *Cancer Med.* **6**, 2471–2486 (2017).

165. C. B. Ambrosone, S. Kropp, J. Yang, S. Yao, P. G. Shields, J. Chang-Claude, Cigarette smoking, *N-acetyltransferase* 2 genotypes, and breast cancer risk: Pooled analysis and meta-analysis. *Cancer Epidemiol. Biomarkers Prev.* **17**, 15–26 (2008).

166. M. M. Gaudet, S. M. Gapstur, J. Sun, W. R. Diver, L. M. Hannan, M. J. Thun, Active smoking and breast cancer risk: Original cohort data and meta-analysis. *J. Natl. Cancer Inst.* **105**, 515–525 (2013).

167. A. Macacu, P. Autier, M. Boniol, P. Boyle, Active and passive smoking and risk of breast cancer: A meta-analysis. *Breast Cancer Res. Treat.* **154**, 213–224 (2015).

168. "Breast cancer: Potential risk factors" (American Cancer Society, 2021); www.cancer.org/cancer/breast-cancer/risk-and-prevention/factors-with-unclear-effects-on-breast-cancer-risk.html.

169. J. J. Yang, D. Yu, Y. Takata, S. A. Smith-Warner, W. Blot, E. White, K. Robien, Y. Park, Y.-B. Xiang, R. Sinha, D. Lazovich, M. Stampfer, R. Tumino, D. Aune, K. Overvad, L. Liao, X. Zhang, Y.-T. Gao, M. Johansson, W. Willett, W. Zheng, X.-O. Shu, Dietary fat intake and lung cancer risk: A pooled analysis. *J. Clin. Oncol. Off. J. Am. Soc. Clin. Oncol.* **35**, 3055–3064 (2017).

170. *Diet, Nutrition, Physical Activity and Lung Cancer* (Continuous Update Project Expert Report 2018, World Cancer Research Fund/American Institute for Cancer Research); www.wcrf.org/wp-content/uploads/2021/02/lung-cancer-report.pdf.

171. L. B. Turner, A meta-analysis of fat intake, reproduction, and breast cancer risk: An evolutionary perspective. *Am. J. Hum. Biol.* **23**, 601–608 (2011).

172. B. Yang, X.-L. Ren, Y.-Q. Fu, J.-L. Gao, D. Li, Ratio of n-3/n-6 PUFAs and risk of breast cancer: A meta-analysis of 274135 adult females from 11 independent prospective studies. *BMC Cancer* **14**, 105 (2014).

173. Y. Cao, L. Hou, W. Wang, Dietary total fat and fatty acids intake, serum fatty acids and risk of breast cancer: A meta-analysis of prospective cohort studies. *Int. J. Cancer* **138**, 1894–1904 (2016).

174. Y. Zhou, S. Zhang, Z. Li, J. Zhu, Y. Bi, Y. Bai, H. Wang, Maternal benzene exposure during pregnancy and risk of childhood acute lymphoblastic leukemia: A meta-analysis of epidemiologic studies. *PLOS ONE* **9**, e110466 (2014).

175. Y. Huang, J. Huang, H. Lan, G. Zhao, C. Huang, A meta-analysis of parental smoking and the risk of childhood brain tumors. *PLOS ONE* **9**, e102910 (2014).

176. C. Metayer, E. Petridou, J. M. M. Aranguré, E. Roman, J. Schüz, C. Magnani, A. M. Mora, B. A. Mueller, M. S. P. de Oliveira, J. D. Dockerty, K. McCauley, T. Lightfoot, E. Hatzipantelis, J. Rudant, J. Flores-Lujano, P. Kaatsch, L. Miligi, C. Wesseling, D. R. Doody, M. Moschovi; MIGICCL Group, L. Orsi, S. Mattioli, S. Selvin, A. Y. Kang, J. Clavel, Parental tobacco smoking and acute myeloid leukemia: The Childhood Leukemia International Consortium. *Am. J. Epidemiol.* **184**, 261–273 (2016).

177. IARC, "Agents classified by the IARC monographs, volumes 1–13"1" (IARC Monographs on the Identification of Carcinogenic Hazards to Humans, 2022); https://monographs.iarc.who.int/agents-classified-by-the-iarc/.

178. J. Wu, R. Zeng, J. Huang, X. Li, J. Zhang, J. C.-M. Ho, Y. Zheng, Dietary protein sources and incidence of breast cancer: A dose-response meta-analysis of prospective studies. *Nutrients* **8**, 730 (2016).

179. J. J. Anderson, N. D. M. Darwis, D. F. Mackay, C. A. Celis-Morales, D. M. Lyall, N. Sattar, J. M. R. Gill, J. P. Pell, Red and processed meat consumption and breast cancer: UK Biobank cohort study and meta-analysis. *Eur. J. Cancer* **90**, 73–82 (2018).

180. M. S. Farvid, M. C. Stern, T. Norat, S. Sasazuki, P. Vineis, M. P. Weijenberg, A. Wolk, K. Wu, B. W. Stewart, E. Cho, Consumption of red and processed meat and breast cancer incidence: A systematic review and meta-analysis of prospective studies. *Int. J. Cancer* **143**, 2787–2799 (2018).

181. C. Chen, X. Ma, M. Zhong, Z. Yu, Extremely low-frequency electromagnetic fields exposure and female breast cancer risk: A meta-analysis based on 24,338 cases and 60,628 controls. *Breast Cancer Res. Treat.* **123**, 569–576 (2010).

182. Q. Chen, L. Lang, W. Wu, G. Xu, X. Zhang, T. Li, H. Huang, A meta-analysis on the relationship between exposure to ELF-EMFs and the risk of female breast cancer. *PLOS ONE* **8**, e69272 (2013).

183. Y. Zhang, J. Lai, G. Ruan, C. Chen, D. W. Wang, Meta-analysis of extremely low frequency electromagnetic fields and cancer risk: A pooled analysis of epidemiologic studies. *Environ. Int.* **88**, 36–43 (2016).

184. "Electromagnetic fields (EMF) and breast cancer risk" (Susan G Komen, 2021); www.komen.org/breast-cancer/facts-statistics/research-studies/topics/electromagnetic-fields-and-breast-cancer-risk/.

185. M. Ding, S. N. Bhupathiraju, A. Satija, R. M. van Dam, F. B. Hu, Long-term coffee consumption and risk of cardiovascular disease: A systematic review and a dose-response meta-analysis of prospective cohort studies. *Circulation* **129**, 643–659 (2014).

186. S. C. Larsson, N. Drca, M. Jensen-Urstad, A. Wolk, Coffee consumption is not associated with increased risk of atrial fibrillation: Results from two prospective cohorts and a meta-analysis. *BMC Med.* **13**, 207 (2015).

187. D. Caldeira, C. Martins, L. B. Alves, H. Pereira, J. J. Ferreira, J. Costa, Caffeine does not increase the risk of atrial fibrillation: A systematic review and meta-analysis of observational studies. *Heart* **99**, 1383–1389 (2013).

188. "Caffeine and heart disease" (American Heart Association, 2014); www.heart.org/en/healthy-living/healthy-eating/eat-smart/nutrition-basics/caffeine-and-heart-disease.

189. Dietary Guidelines Advisory Committee, *Scientific Report of the 2015 Dietary Guidelines Advisory Committee: Advisory Report to the Secretary of Health and Human Services and the Secretary of Agriculture* (U.S. Department of Agriculture, Agricultural Research Service, 2015).

190. C. Pelucchi, C. Bosetti, E. Negri, L. Lipworth, C. La Vecchia, Olive oil and cancer risk: An update of epidemiological findings through 2010. *Curr. Pharm. Des.* **17**, 805–812 (2011).

191. Y. Xin, X.-Y. Li, S.-R. Sun, L.-X. Wang, T. Huang, Vegetable oil intake and breast cancer risk: A meta-analysis. *Asian Pac. J. Cancer Prev.* **16**, 5125–5135 (2015).

192. X. Yu, Z. Bao, J. Zou, J. Dong, Coffee consumption and risk of cancers: A meta-analysis of cohort studies. *BMC Cancer* **11**, 96 (2011).

193. F. Turati, C. Galeone, V. Edefonti, M. Ferraroni, P. Lagiou, C. La Vecchia, A. Tavani, A meta-analysis of coffee consumption and pancreatic cancer. *Ann. Oncol.* **23**, 311–318 (2012).

194. K. Nie, Z. Xing, W. Huang, W. Wang, W. Liu, Coffee intake and risk of pancreatic cancer: An updated meta-analysis of prospective studies. *Minerva Med.* **107**, 270–278 (2016).

195. IARC Working Group on the Evaluation of Carcinogenic Risks to Humans, *Drinking Coffee, Mate, and Very Hot Beverages* (International Agency for Research on Cancer, 2018), vol. 116; www.ncbi.nlm.nih.gov/books/NBK543953/.

196. *Diet, Nutrition, Physical Activity and Pancreatic Cancer* (Continuous Update Project Expert Report 2018, World Cancer Research Fund/American Institute for Cancer Research, 2018); www.wcrf.org/wp-content/uploads/2021/02/pancreatic-cancer-report.pdf.

197. Y. Shang, G. Han, J. Li, J. Zhao, D. Cui, C. Liu, S. Yi, Vasectomy and prostate cancer risk: A meta-analysis of cohort studies. *Sci. Rep.* **5**, 9920 (2015).

198. B. Bhindi, C. J. D. Wallis, M. Nayan, A. M. Farrell, L. W. Trost, R. J. Hamilton, G. S. Kulkarni, A. Finelli, N. E. Fleshner, S. A. Boorjian, R. J. Karnes, The association between vasectomy and prostate cancer: A systematic review and meta-analysis. *JAMA Intern. Med.* **177**, 1273–1286 (2017).

199. T. Wu, X. Duan, S. Chen, C. Hu, R. Yu, S. Cui, Does vasectomy increase prostate cancer risk? An updated meta-analysis and systematic review of cohort studies *Int. J. Clin. Exp. Med.* **11**, 2978–2987 (2018).

200. I. D. Sharlip, A. M. Belker, S. Honig, M. Labrecque, J. L. Marmar, L. S. Ross, J. I. Sandlow, D. C. Sokal, Vasectomy: AUA guideline. *J. Urol.* **188**, 2482–2491 (2012).

201. “Prostate cancer risk factors” (American Cancer Society, 2020); www.cancer.org/cancer/prostate-cancer/causes-risks-prevention/risk-factors.html.

202. A. K. Hackshaw, E. A. Paul, Breast self-examination and death from breast cancer: A meta-analysis. *Br. J. Cancer* **88**, 1047–1053 (2003).

203. J. P. Kösters, P. C. Gøtzsche, Regular self-examination or clinical examination for early detection of breast cancer. *Cochrane Database Syst. Rev.*, CD003373 (2003).

204. “Breast cancer risk assessment and screening in average-risk women” (Practice Bulletin 179, American College of Obstetricians and Gynecologists, 2017); www.acog.org/en/clinical/clinical-guidance/practice-bulletin/articles/2017/07/breast-cancer-risk-assessment-and-screening-in-average-risk-women.

205. “ACS breast cancer screening guidelines” (American Cancer Society, 2022); www.cancer.org/cancer/breast-cancer/screening-tests-and-early-detection/american-cancer-society-recommendations-for-the-early-detection-of-breast-cancer.html.

206. “Breast cancer screening (PDQ®)–Health professional version” (National Cancer Institute, 2022); www.cancer.gov/types/breast/hp/breast-screening-pdq.

207. V. Beral, D. Bull, R. Doll, R. Peto, G. Reeves, Collaborative Group on Hormonal Factors in Breast Cancer, Breast cancer and abortion: Collaborative reanalysis of data from 53 epidemiological studies, including 83?000 women with breast cancer from 16 countries. *Lancet* **363**, 1007–1016 (2004).

208. Y. Huang, X. Zhang, W. Li, F. Song, H. Dai, J. Wang, Y. Gao, X. Liu, C. Chen, Y. Yan, Y. Wang, K. Chen, A meta-analysis of the association between induced abortion and breast cancer risk among Chinese females. *Cancer Causes Control* **25**, 227–236 (2014).

209. J. Guo, Y. Huang, L. Yang, Z. Xie, S. Song, J. Yin, L. Kuang, W. Qin, Association between abortion and breast cancer: An updated systematic review and meta-analysis based on prospective studies. *Cancer Causes Control* **26**, 811–819 (2015).

210. “Abortion and breast cancer risk” (Susan G Komen, 2021), www.komen.org/breast-cancer/facts-statistics/research-studies/topics/abortion-and-breast-cancer-risk/.

211. “Induced abortion and breast cancer risk” (ACOG Committee Opinion 434, American College of Obstetricians and Gynecologists, 2009); www.acog.org/en/clinical/clinical-guidance/committee-opinion/articles/2009/06/induced-abortion-and-breast-cancer-risk.

212. M. López-Cervantes, L. Torres-Sánchez, A. Tobías, L. López-Carrillo, Dichlorodiphenyldichloroethane burden and breast cancer risk: A meta-analysis of the epidemiologic evidence. *Environ. Health Perspect.* **112**, 207–214 (2004).

213. S. Z. Ingber, M. C. Buser, H. R. Pohl, H. G. Abadin, H. E. Murray, F. Scinicariello, DDT/DDE and breast cancer: A meta-analysis. *Regul. Toxicol. Pharmacol.* **67**, 421–433 (2013).

214. J.-H. Park, E. S. Cha, Y. Ko, M.-S. Hwang, J.-H. Hong, W. J. Lee, Exposure to dichlorodiphenyltrichloroethane and the risk of breast cancer: A systematic review and meta-analysis. *Osong. Public Health Res. Perspect.* **5**, 77–84 (2014).

215. IARC Working Group on the Evaluation of Carcinogenic Risks to Humans, *DDT, Lindane, and 2,4-D* (International Agency for Research on Cancer, 2018), vol. 113; www.ncbi.nlm.nih.gov/books/NBK507424/.

216. J. R. Roberts, J. R. Reigart, *Recognition and Management of Pesticide Poisonings* (U.S. Environmental Protection Agency, ed. 6, 2013); www.epa.gov/pesticide-worker-safety/recognition-and-management-pesticide-poisonings.

217. M. Elcock, R. W. Morgan, Update on artificial sweeteners and bladder cancer. *Regul. Toxicol. Pharmacol.* **17**, 35–43 (1993).

218. IARC Working Group on the Evaluation of Carcinogenic Risks to Humans, *Some Chemicals that Cause Tumours of the Kidney or Urinary Bladder in Rodents and Some Other Substances* (International Agency for Research on Cancer, 1999), vol. 73; www.ncbi.nlm.nih.gov/books/NBK402050/.

219. “Artificial sweeteners and cancer” (National Cancer Institute, 2005); www.cancer.gov/about-cancer/causes-prevention/risk/diet/artificial-sweeteners-fact-sheet).
